# Two phases for centripetal migration of *Drosophila melanogaster* follicle cells: initial ingression followed by epithelial migration

**DOI:** 10.1242/dev.200492

**Published:** 2023-03-30

**Authors:** Travis T. Parsons, Sheila Mosallaei, Laurel A. Raftery

**Affiliations:** School of Life Sciences, University of Nevada, 4505 S. Maryland Parkway, Las Vegas, NV 89154-4004, USA

**Keywords:** Collective cell migration, Ingression, Epithelial organization, Oogenesis, Centripetal migration, Cadherin adhesion

## Abstract

During *Drosophila* oogenesis, somatic follicle cells (FCs) differentiate to secrete components of the eggshell. Before secretion, the epithelium reorganizes to shape eggshell specializations, including border FC collective cell migration and later dorsal formation. These FC movements provide valuable insights into collective cell migration. However, little is known about centripetal migration, which encloses the oocyte after secretion has begun. Centripetal migration begins with apical extension of a few FCs that move away from the basement membrane to invade between germ cells. We define a timeline of reproducible milestones, using time-lapse imaging of egg chamber explants. Inward migration occurs in two phases. First, leading centripetal FCs ingress, extending apically over the anterior oocyte, and constricting basally. Second, following FCs move collectively toward the anterior, then around the corner to move inward with minimal change in aspect ratio. E-cadherin was required in leading centripetal FCs for their normal ingression, assessed with homozygous *shotgun* mutant or RNAi knockdown clones; ingression was influenced non-autonomously by mutant following FCs. This work establishes centripetal migration as an accessible model for biphasic E-cadherin-adhesion-mediated collective migration.

## Teaser

**Summary:** Secretory somatic cells enclose the *Drosophila* oocyte by invasive ingression between germ cells, followed by collective migration of three more cells, reorganizing to build eggshell over the anterior oocyte.

## INTRODUCTION

Cell migration *in vivo* has marked differences to the two-dimensional migration studied in cell culture (Campbell and Casanova, 2016; Friedl and Gilmour, 2009; Friedl and Mayor, 2017; SenGupta et al., 2021). Notwithstanding, the best understood migratory mechanisms share similarities with traditional cell culture systems, involving epithelial-mesenchymal transition and/or integrin-mediated migration over a basement membrane extracellular matrix. For collective cell migration, lateral cell-cell adhesion coordinates migrating cells (Friedl and Gilmour, 2009). Cadherin-based adhesion is relatively passive in such collectives, whereas Integrin-extracellular matrix adhesion gives traction. Here, we use real-time imaging to examine E-cadherin-dependent invasive migration.

Cadherin-based migration is an emerging mechanism observed *in vivo*; best studied in zebrafish primordial germ cell migration and *Drosophila* ovarian border cell migration (Cai et al., 2014; Kardash et al., 2010). Evidence for cadherin-mediated migration was reported for *Drosophila* ovarian centripetal migration, a later somatic cell migration into the germ cell cluster (Oda et al., 1997; Tepass et al., 1996). We have discriminated between proposed invagination and delamination mechanisms for centripetal migration, and finely mapped E-cadherin requirements for successful migration.

**Fig. 1.**
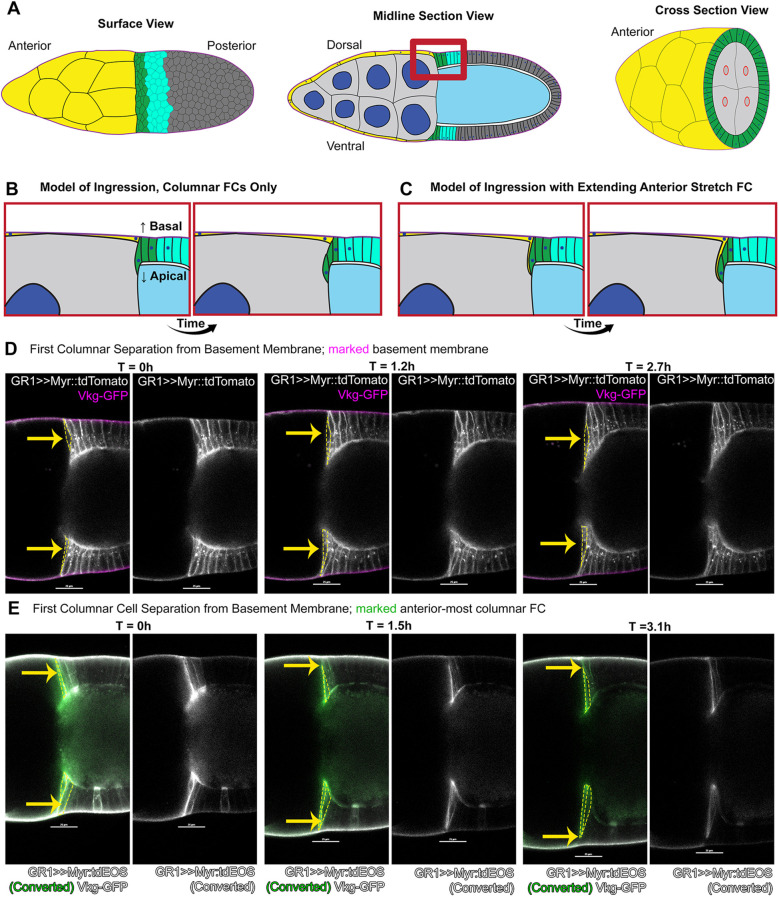
**Centripetally migrating follicle cells reduce contact with the basement membrane.** (A) Diagrams of stage 10A/10B egg chambers. Anterior to left. Leading centripetal follicle cells (FCs) in green, following centripetal FCs in turquoise, non-migratory mainbody FCs in dark gray. Stretch FCs (yellow) surround nurse cells, which are light gray with dark blue nuclei in midline and cross-section views. Oocyte is light blue. Red box corresponds to models in B and C. Cross-section shows nurse cells at the interface with oocyte; ring canals (red) connect nurse cells to oocyte. Centripetally migrating FCs move inward to cover this surface. (B) Leading FCs elongate inward between nurse cell and oocyte, and successively move inward. (C) Leading centripetal FCs extend inward in tandem with stretch FCs. The leading centripetal FCs detach from basement membrane, but the extending stretch FC retains contact. (D) Leading centripetal FCs thin basal contact with the basement membrane progressively in selected time points of early migration. FC membranes marked with Myr::tdTomato (white); basement membrane with GFP-tagged Collagen IV (magenta). Merged fluorescence on left, Myr::tdTomato alone on right. Yellow arrows mark leading centripetal FCs, which are outlined by yellow dashed lines in left images. (E) Basal thinning of leading FCs highlighted by photoconversion of tdEOS (green) in the first few FCs, with unconverted tdEOS (white) in more-posterior FCs, and GFP::Collagen IV in basement membrane (white). Note gap between green FC tdEOS and white basement membrane GFP at 3.1 h. Scale bars: 25 µm. For this and all subsequent figures, genotypes are listed in Table S2.

Centripetal migration occurs in *Drosophila* ovarian egg chambers, a genetically tractable system (Duhart et al., 2017; Horne-Badovinac and Bilder, 2005). Each egg chamber includes a cluster of 16 germ cells surrounded by a somatic epithelium of follicle cells (FCs) (Fig. 1A). The most posterior germ cell becomes an oocyte; the remaining 15 become nurse cells, which contribute cytoplasmic contents to the oocyte. Numerous egg chambers are readily accessible, because females lay eggs continuously (King, 1970; Spradling, 1993).

FCs reorganize extensively before secreting eggshell components (Margaritis, 1986; Waring, 2000). Until mid-oogenesis, the FC epithelium migrates circumferentially, strengthening the basement membrane to constrain egg shape (Gutzeit et al., 1991; Isabella and Horne-Badovinac, 2015a,b). Epithelial morphogenesis begins in stage 9, when the border cell cluster delaminates to migrate through nurse cells to the anterior oocyte (Montell et al., 1992). Simultaneously, anterior FCs flatten to form stretch cells, covering nurse cells (Brigaud et al., 2015; King and Vanoucek, 1960). Remaining FCs form a columnar secretory epithelium covering the oocyte, except for the anterior face that contacts nurse cells (King and Vanoucek, 1960). FCs begin secreting vitelline membrane components during late stage 9 (King, 1960; Logan and Wensink, 1990). To complete the anterior eggshell, FCs migrate inward, or centripetally, to enclose the oocyte, separating it from nurse cells (Fig. 1A-C). When FCs fail to cover the anterior oocyte, an ‘open’ cup-like eggshell forms (Schüpbach and Wieschaus, 1991).

Centripetal migration is a rare example of epithelial plasticity after differentiation (Gustafson and Wolpert, 1963; Pilot and Lecuit, 2005; Shook and Keller, 2003). Inward migration begins after onset of secretion, as extracellular membrane ‘bodies’ accumulate over the oocyte (Andrenacci et al., 2001; Cavaliere et al., 2008; King, 1960). How secretory FCs retain plasticity for centripetal migration is unknown. A systematic description of the migration would help make centripetal migration a tractable system to interrogate retained plasticity for migration in differentiated epithelial cells.

Gene regulatory networks are defined for anterior FCs, including centripetal FCs (Fauré et al., 2014; Fregoso Lomas et al., 2016; Pyrowolakis et al., 2017; Revaitis et al., 2017; Yakoby et al., 2008a). Specific gene expression is detected in two or three rings of anterior columnar FCs about 6 h before they begin inward movement. A Notch-C/EBP-Cux homeodomain gene regulatory network activates apical elongation of the first centripetally migrating FCs (Dobens et al., 2005; Levine et al., 2010, 2007). Upregulation of E-cadherin and associated adherens junction components accompanies initial apical elongation (Edwards and Kiehart, 1996; Niewiadomska et al., 1999).

The mechanism for centripetal migration has been obscure, mentioned as either delamination or invagination with stretch FCs (Levine et al., 2010; Niewiadomska et al., 1999; Tran and Berg, 2003). To identify temporal dynamics and resolve individual cells participating in centripetal migration, we adapted methods for time-lapse imaging of *ex vivo* egg chambers to stages 10 and 11. Our data indicate that centripetal migration occurs in two phases. First, the traditionally-defined centripetally migrating FCs (simplified to centripetal FCs) elongate apically, undergo basal constriction and move inward from the basement membrane; these are the leading centripetal FCs. Second, adjacent rows of columnar FCs follow inward, but retain epithelial organization as they migrate around the junction of outer and anterior oocyte surfaces; these we call the following FCs.

We tested the requirement for E-cadherin, encoded by *shotgun* (*shg*), in each population associated with migration. Leading centripetal FCs autonomously required E-cadherin for both apical extension and basal thinning to ingress. E-cadherin may be dispensable in following FCs, as long as leading FCs migrated normally. E-cadherin in germ cells was required for their normal organization, which influenced morphology for leading FC ingression; misplaced germ cells could stall migration. Altogether, this work establishes centripetal migration as a model to investigate apically-directed ingression by cadherin-mediated adhesion, providing both live-imaging methodology and a timeline for migration.

## RESULTS

To examine the 6 h of centripetal migration, we adapted the *ex vivo* culture method for stage 9 egg chambers (Prasad et al., 2007) to stages 10 and 11. Although the value of time-lapse imaging to study temporal progression is obvious, limitations exist. Stage 10 egg chambers exhibit germ cell growth, are sensitive to crowding and require delicate handling during preparation for imaging. Imaging conditions must accommodate increasing tissue diameter over 5-6 h. We selected genetically-encoded fluorescent proteins that were robust and bright, to minimize photobleaching and photodamage. These were further minimized by limiting visualization to two colors, and only acquiring 10-13 optical sections every 8-10 min. We used live-imaging to establish a timeline with milestones, and for *in vivo* RNA interference (RNAi) experiments.

In contrast, immunofluorescent staining uses a rainbow of optimized fluorophores, permitting four color imaging. Fixed tissues have a longer life, so that more samples can be imaged from one preparation. We chose immuno­fluorescence for three-color imaging, and to analyze mosaic tissues generated by mitotic recombination.

For both methods, imaging system availability affects experimental design, including fluorophore selection and resolution in time and space.

### Centripetal migrating columnar FCs reduce contact with the basement membrane

Centripetal migration has been called either invagination or delamination. Invagination was suggested by a report of stretch FC inward movement alongside columnar FCs (Tran and Berg, 2003), centering at the junction between these cell types (Levine et al., 2010). However, the elongated morphology of centripetal columnar FCs suggested that traditional epithelial invagination was unlikely (Pilot and Lecuit, 2005; Gilmour et al., 2017; Keller and Shook, 2011; Osterfield et al., 2013; Sawyer et al., 2010).

We first examined morphology of columnar centripetal FCs. These FCs move in the apical direction, distinct from basally-directed epithelial-mesenchymal transition (Nieto et al., 2016). Extrusion is an apically-directed delamination, where the basal protrusions of neighboring cells separate the delaminating cell from the epithelium (Mori et al., 2022). Alternatively, FC delamination could occur by ingression, with basal constriction of centripetal FCs as they move inward (Fig. 1B,C).

We focused on columnar FCs that lead migration by elongating first (Fig. 1B; Movie 1). Before migration, columnar FCs adhere to the basement membrane (Delon and Brown, 2009). For live-imaging, we marked the basement membrane with GFP-tagged collagen IV (GFP::Collagen) and examined the basal side of centripetal FCs, with membranes marked by myristoylated-tdTomato (Myr::tdTomato) (Fig. 1D). Cell movements were imaged for up to 6 h.

Leading centripetal FC basal regions near the basement membrane progressively thinned to undetectable (33 of *n*=33). To confirm basal thinning, we marked the leading one to two centripetal FCs by induced fluorescence switch of a ubiquitous membrane tag, myristoylated-tdEOS (Myr::tdEOS), expressed throughout FCs (Fig. 1E; Movie 1). For samples switched early enough, time-lapse data confirmed a progressive decrease in contact with the basement membrane (8 of *n*=12). Each photo-switched leading centripetal FC elongated apically and thinned basally, successively adopting a spindle-like shape (Fig. 1D,E). This morphology appeared to be consistent with ingression of these leading centripetal FCs, and inconsistent with infolding or extrusion.

### Initial phase: leading FC ingression

**Fig. 2.**
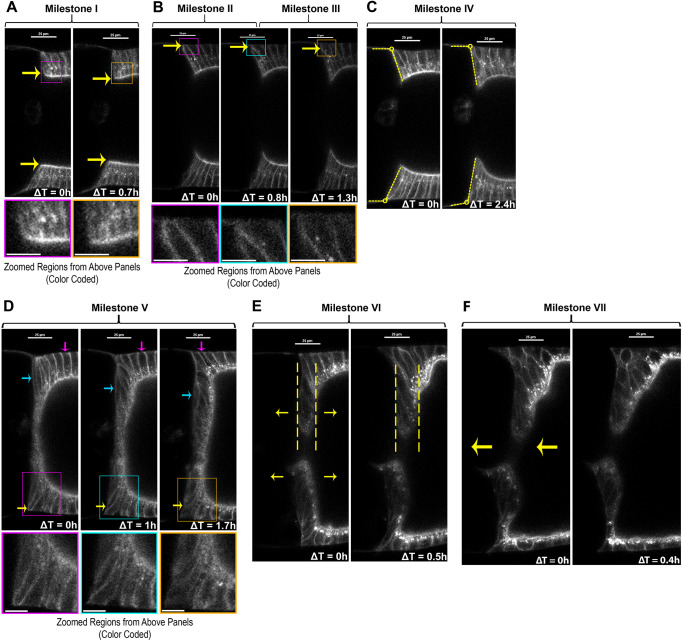
**Representative images of centripetal migration milestones.** (A-F) Changing morphology for each milestone shown with time points from time-lapse sequences, single midline section optical sections; FCs labeled with Myr::tdTomato (white). Each image set selected from a different egg chamber, colored box in top panel indicates region enlarged in bottom panel. Diameter of oocyte increased throughout stage 10, possibly from lipid uptake (compare early and later time points in Milestones I-VI). (A) Milestone I: anterior-most FCs started at uniform columnar height (left), then initiated apical elongation (yellow arrows), extending beyond adjacent FCs (right). (B) Milestone II: first leading FC initiated basal thinning at basement membrane (left to middle, yellow arrows). Milestone III: basal separation of lateral sides became undetectable for first leading FC (middle to right), sometimes with detectable inward movement of basal tip (yellow arrows). (C) Milestone IV: interface between anterior-most columnar FC and adjacent nurse cell was angled during stage 10A (left, yellow dashed lines), then shifted to nearly vertical with variable timing between Milestone I and Milestone V (yellow dashed lines, right). (D) Milestone V: after the first three leading centripetal FCs had undetectable contact with the basement membrane, the morphology of following FCs followed a distinct progression, from outer epithelium (magenta arrow), around corner (yellow arrow), to anterior inner epithelium (blue arrow). In this example, the corner FC had a more pentagonal shape (yellow arrow), then resumed a more quadrilateral shape in anterior epithelium. Dorsal following FCs exhibited more extensive movement and reorganization than ventral. (E) Milestone VI: FCs lining the anterior oocyte lengthened their FC:FC interfaces slightly, so the epithelium appeared to thicken just before onset of nurse cell dumping (note greater distance between dashed lines in left versus right). (F) Milestone VII: when nurse cell dumping began, the oocyte rapidly enlarged, causing an anterior shift of FCs covering the anterior oocyte surface (arrows, left). Scale bars: 25 µm; ∼10 µm in magnifications.

To follow progression of centripetal migration, we assessed centripetal columnar FCs for reproducible changes in morphology or behavior. Seven milestones were defined from analysis of 33 egg chambers (Fig. 2). Milestone I marked onset of migration: anterior-most columnar FCs lengthened apically, extending beyond their posterior neighbors (mean length increase=10 µm, s.d.=1 µm, *n*=7; Fig. 2A; Fig. S1A). Milestone II marked onset of basal thinning in elongating FCs, starting from a median width of 3.7 µm (Fig. 2B, left; Fig. S1B). Milestone III marked loss of detectable contact between the first leading centripetal FC and the basement membrane (Fig. 2B, right), the timing showed no significant correlation with initial width (Fig. S1C,D). We tested the actual median Milestone II width against a theoretical median of 0.3 µm (median limit of detection at Milestone III), and found they significantly differed (*P*<0.0001; two-tailed Wilcoxon signed rank sum test). Additional leading centripetal FCs reduced basal contact during the period between Milestone III and Milestone V.

Inward movement of leading centripetal FCs was reflected by positions of their nuclei. Columnar FC nuclei were basal and remained basal in posterior ‘mainbody’ FCs. In leading centripetal FCs, nuclei shifted towards the leading edge as elongation and ingression progressed. Thus, nuclear position provided a clue to leading FC position during Milestones I-III (Fig. S2; Mosallaei, 2021), but did not include a nuclear marker in the experiment to establish morphological milestones.

**Fig. 3.**
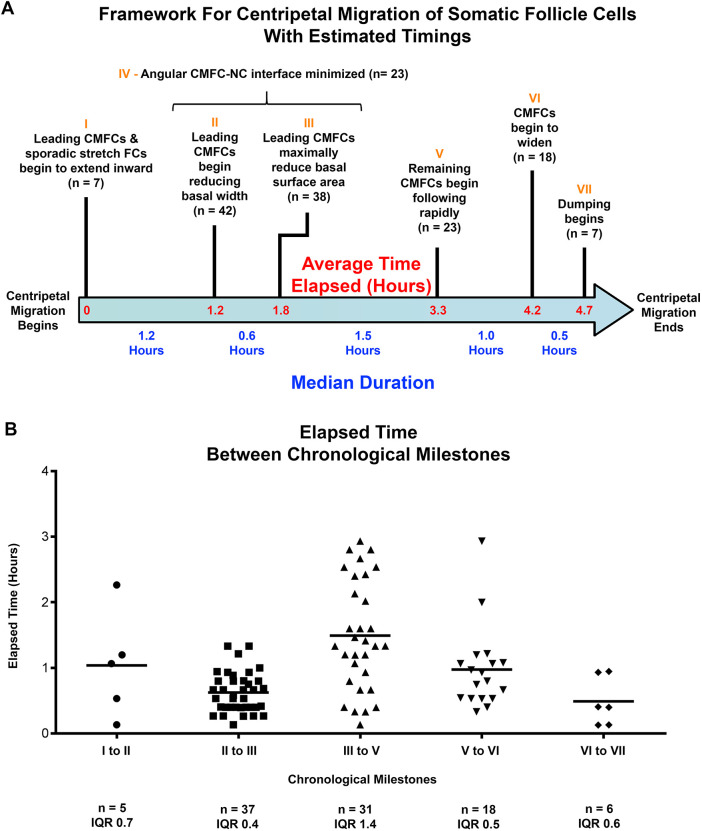
**Timeline of milestones for centripetal migration.** (A) Milestones for centripetal migration shown along timeline (blue arrow), with average total elapsed time (red). Milestones I-VII (orange) are above the arrow, with a short description and instances observed (*n*). Timing of Milestone IV varies over the period of Milestones II-III. For other milestones smaller variations in onset were observed. Median duration between milestones below the arrow (blue text) was determined from time lapse sequences of 33 samples, each including at least two milestones. Average time elapsed was calculated as the sum of average durations from the same timestamp data (red on blue arrow). (B) Scatter plots of elapsed time between sequential milestones (labels I-VII correspond to timeline above). The number of intervals measured (*n*) and the interquartile range (IQR) are listed below each. Median shown in blue on timeline in A.

During this period, appearance of the FC:nurse cell interface changed. Prior to Milestone IV, the anterior nurse cell edge formed an obtuse-angled interface with the adjacent FC epithelium (Fig. 2C, left). At Milestone IV, this angle had significantly reduced to approach a right angle (Fig. 2C; Fig. S1E). Timing of Milestone IV varied, occurring between Milestone I and Milestone V (Fig. 3A). Milestone IV and later milestones are influenced extrinsically by germ cells and other factors (discussed below).

### Second phase: following FC inward migration

After three leading centripetal FCs ingressed with a spindle shape, migration of more posterior following FCs changed, marking Milestone V. During the Milestone III-V transition, the anterior oocyte surface became more linear, with tighter ‘corners’ (compare Fig. 2D left with Fig. 2D right). Following FCs moved along the outer surface to the anterior with a rounded, quadrilateral shape. They briefly adopted a roughly pentagonal shape as they moved around the oocyte corner, and usually resumed a roughly quadrilateral shape as they moved further inward (Fig. 2D). Individual following FCs showed no significant difference in aspect ratio of FC:FC interface height to FC width in both outside and inside anterior epithelia (Fig. 2D; Fig. S1F). This change in migration morphology appeared to be coincident with outer mainbody FC widening over the enlarging oocyte.

Differences in migration morphology between leading centripetal FCs and following centripetal FCs indicated two phases for centripetal migration. In the first phase, the leading three centripetal FCs elongated into a spindle shape as they moved away from the basement membrane, then shortened to join the inner anterior epithelium. In the second phase, following centripetal FCs minimally changed shape as they moved over the junction of outer to inner epithelium, and maintained the same side at the FC:oocyte surface.

In Milestone VI, the FC epithelium thickened significantly over the anterior oocyte. Individual FC outlines were faint at this stage, so that reorganization of the anterior epithelium was difficult to discern (Fig. 2E). However, the epithelial anterior and posterior edges were detectable, so distance between these edges was measured to assess thickening, with a significant difference in thickness after 1 h (width in Fig. S1G). By Milestone VI, we noticed dense accumulation of puncta with strong Myr::tdTomato fluorescence in outer FCs and some anterior FCs (Fig. 2D-F). This might indication a resumption of secretory activity, but we did not investigate further.

Shortly before nurse cell dumping, opposing leading edges of centripetal FCs averaged 18 µm apart (s.d.=6 µm, *n*=20). The four ring canals, connecting oocyte to nurse cells, were clustered here (Fig. 1A; Fig. S3).

Milestone VII marked onset of nurse cell dumping, when rapidly increasing oocyte volume pushed the centripetal FC epithelium towards the anterior egg chamber (mean displacement=23 µm, s.d.=12 µm, *n*=7; Fig. 2F; Fig. S1H shows median). This shift disrupted subsequent observations. Annotated time-lapse videos of all milestones are in Movie 2.

We assembled a timeline for centripetal migration using the median duration between each pair of milestones (Fig. 3A). Notably, six of the seven milestones occurred in reproducible chronological order (33 of *n*=33). Milestone IV occurred during the Milestone II-III time period. Total duration from Milestone I to Milestone VII averaged 4.7 h, consistent with previous estimates for stage 10B, defined by centripetal migration (King, 1970; Lin and Spradling, 1993; Mahowald and Kambysellis, 1980). Although the total elapsed time for centripetal migration was consistent, duration of each step between milestones varied (Fig. 3B).

### Centripetal migration sporadically included extension of adjacent stretch FCs

**Fig. 4.**
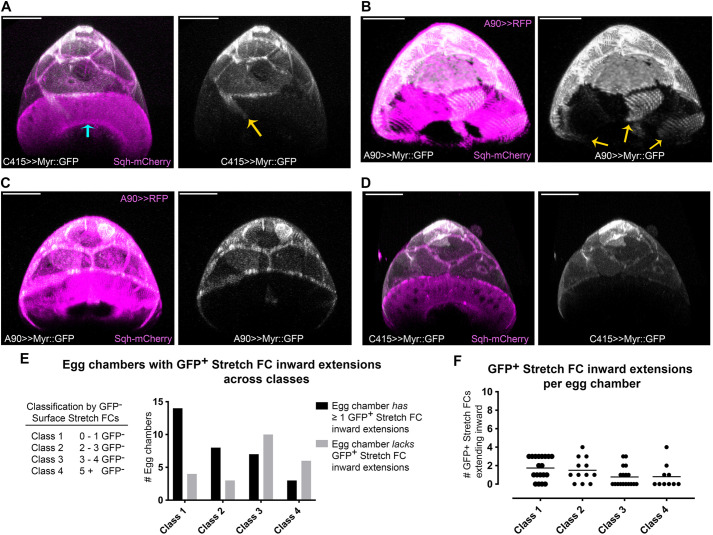
**Stretch FCs sporadically extend inward with leading FCs.** (A-D) Digitally-rendered cross-sections at the stretch FC:centripetal FC interface, showing anterior volumetric projections of half egg chambers at the end of stage 10B. Stretch FC membranes labeled with UAS-Myr::GFP expression (white) using a different stretch FC-specific Gal4 driver in each panel. Columnar FCs express Sqh-mCherry (magenta) from the endogenous *sqh* promoter. Merged fluorescence on left; stretch FC Myr::GFP alone on right (white). (A) C415-Gal4 (Manseau et al., 1997). (B) Similarly, A90-Gal4 (Yeh et al., 1995) revealed sporadic inward extension (yellow arrows indicate three extensions). (C,D) With either A90-Gal4 (C) or C415-Gal4 (D), some egg chambers had no stretch FC extensions detectable. (E) Egg chambers were classified according to number of unlabeled stretch FCs visible (Class legend). Class 1 egg chambers had ≤1 unlabeled stretch FC; class 4 egg chambers had ≥5 unlabeled stretch FCs. Within each class, numbers of egg chambers with or without detectable stretch FC extensions were quantified and summed across these Gal4 drivers (graph). (F) Across all Gal4 drivers, the number of extending stretch FCs in each egg chamber is displayed for each class. Scale bars: 50 μm.

We next sought to assess whether inward extension of stretch FC extensions could be reliably detected during migration. Stretch FCs were marked with a membrane tag (UAS-myristoylated-GFP; Myr::GFP). Because available ‘stretch FC-specific’ Gal4 drivers had different posterior edges, we repeated the experiment with each driver: A90-Gal4, C415-Gal4 and PG150-Gal4 (Fig. S4). All cell cortical cytoskeletons were marked with Sqh::mCherry (myosin type 2 regulatory light chain, C-terminally tagged with mCherry). Time-lapse sequences were collected through the end of stage 10B, and the final internal stretch FC extensions were visualized in *x*-*z* volume projections of half egg chambers (*n*=59; Fig. 4A-D), each encompassing 10-15 stretch FCs.

Every stretch FC Gal4 driver gave variable expression of myristoylated markers at stage 10B (Fig. S4A-D). All samples were grouped in four classes, based on the number of poorly-labeled stretch FCs visible (Fig. 4E,F). Across classes, consistent proportions of stretch FCs extended inward in tandem with columnar FCs. Comparable results came from photoconverting Myr::tdEOS to mark a few stretch FCs (Fig. S5). Ambiguity in the boundary between stretch FCs and centripetally migrating FCs may reflect the gradual distinction between stretch and columnar FCs during stage 9 (Weichselberger et al., 2022).

Overall, we detected ≥1 stretch FC extension in tandem with leading FCs in nearly two-thirds of late stage 10B egg chambers (61%, 36 of *n*=59; Fig. 4E). Among these half-egg chambers, on average 2 stretch FCs extended inward (s.d.=1; Fig. 4F). Cell-to-cell variability precluded analysis of stretch FC behavior.

### Extrinsic factors influence milestones

Some milestones are strongly influenced by extrinsic changes in neighboring cell types, particularly Milestone IV. Angular morphology of the interface between leading columnar FCs and adjacent nurse cells (marked in Fig. 2C), arose during stage 9, as cuboidal anterior FCs flattened into stretch FCs, proceeding from anterior- to posterior-most (Grammont, 2007; Weichselberger et al., 2022). Conversely, columnar FCs form by cuboidal FCs lengthening their FC:FC interfaces, starting in stage 8, and spreading from posterior to anterior during oocyte growth in stage 9. In our samples, the squamous FC:columnar FC interface was apparent before the onset of centripetal migration (Fig. 1D, middle panels). As leader FC apical extension proceeds during Milestones I-III, oocyte volume increases more rapidly than nurse cell volume because of oocyte uptake of yolk protein from the hemolymph, which contributes to substantial enlargement of the oocyte during stages 8 to 10 (Bownes, 1982). During stage 10, both nurse cells and oocyte take up neutral lipids, increasing the volume of all germ cells during the period of centripetal migration (Sieber and Spradling, 2015). Note that fetal bovine serum (FBS) was used in the culture medium, but not *Drosophila* hemolymph. Overall changes to germ cell volume and shape help to bring the elongating leading FCs into vertical alignment over the nurse cell:oocyte interface.

Continued oocyte growth during the Milestone III-V interval is associated with columnar FC widening to accommodate oocyte expansion along the anterior-posterior (A-P) axis, visible by inspection of panels in Fig. 2 (King, 1970; Spradling, 1993; Weichselberger et al., 2022). Movement of following cells in Milestones V-VI might be associated with further changes in posterior mainbody FCs (Fig. 2D; Movie 2).

Rapid oocyte lengthening occurs from the volume increase due to nurse cell dumping. Thus, Milestone VII may be predominantly dependent on nurse cell events. It is unclear whether this milestone is influenced by centripetal FCs after the anterior epithelium is formed.

### FC E-cadherin was required autonomously and non-autonomously for centripetal migration

Migration morphology differed between ingressing leading FCs and following FCs. We speculated that higher E-cadherin accumulation in leading FCs might indicate a differential requirement compared with following centripetal FCs. A requirement for E-cadherin in centripetal FCs was previously observed using fixed mosaic egg chambers containing *shg* homozygous null cells (Niewiadomska et al., 1999; Oda et al., 1997; Tepass et al., 1996). Aberrantly rounded *shg^−/−^* centripetal FCs were clustered at the periphery of 10B and later egg chambers, suggesting failure of migration. In egg chambers with *shg^−/−^* germ cells, *shg^+^* centripetal FCs moved into the germ cell cluster but had abnormal morphology and sometimes abnormal locations. These data led to speculation that centripetal migration was mediated by E-cadherin-adhesion between centripetal FCs and germ cells.

To dissect requirements for E-cadherin with greater spatial and temporal resolution, we used live imaging of mosaic egg chambers containing clones of cells with decreased E-cadherin. ‘Flipout’ expression of Gal4 was used to activate *in vivo* RNAi so that clones occurred randomly at different positions. Flexible RNAi tools were compatible with fluorescent protein markers amenable for time-lapse imaging at stage 10.

RNAi-activated FCs were visualized by co-expressing GFP with double-stranded RNAs (dsRNAs), against either *shg* or luciferase (Perkins et al., 2015; Pignoni and Zipursky, 1997). Control clones engaged microRNA processing machinery, without targeting a known *Drosophila* gene. Egg chambers with GFP^+^, dsRNA-expressing cells in or near centripetal FCs were selected for time-lapse imaging. Two *shg* dsRNA transgenes were used; one gave stronger knockdown (Fig. S6). However, for statistical analyses, data from both were combined due to low recovery of clones from the stronger strain.

We categorized samples as having GFP^+^ in one to three of the leading FCs, but no following FCs (leaders only), in both leading and following FCs, in variable numbers (both leaders and followers), or in following FCs with at most one leading FC, the third FC (followers only). See Materials and Methods for additional information on scoring specific metrics.

**Fig. 5.**
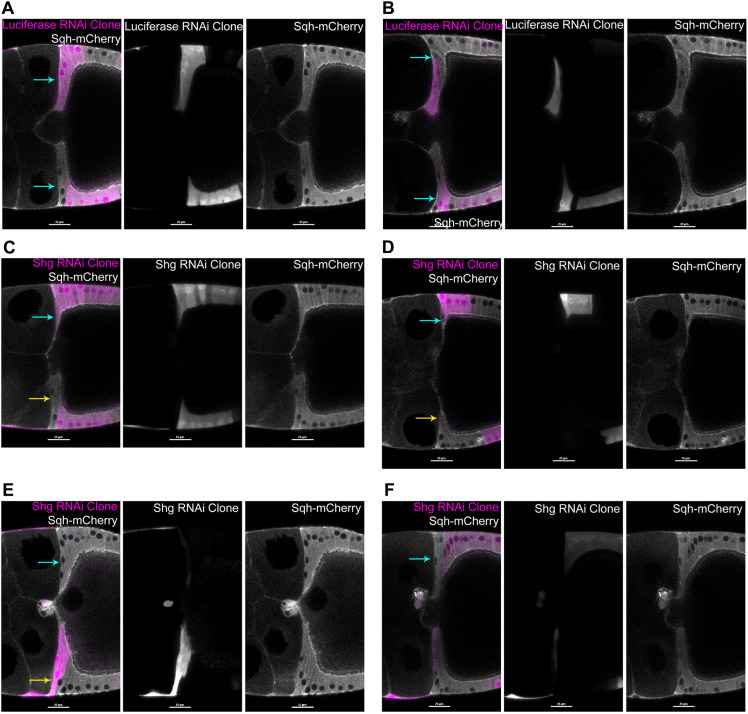
***shg* depletion in follicle cells resulted in abnormal centripetal migration.** (A-F) Double-stranded hairpin RNAs for *luciferase* (A,B) *shg* (C-F) were co-expressed with a GFP reporter (magenta) using the Flipout Gal4 system. Single midline optical sections show clones in or near centripetal FCs. Sqh-mCherry (white) labels all FCs. Left: merged image; center: GFP alone; right: mCherry alone, each white. Anterior is left; dorsal is up except in F. (A,B) Control GFP^+^ clones with untargeted RNAi by *luciferase* dsRNAs showed timely thinning basal contact with the basement membrane (blue arrows in left panels). Three leading FCs exhibited normal organization with ‘vertically stacked nuclei’, regardless of the position and size of the clone. (C) Ingression was severely delayed where both leading centripetal FCs and neighboring posterior FCs were depleted (top of egg chamber, blue arrow on left). Where only more-posterior neighboring FCs were depleted (bottom of egg chamber), the wild-type leading centripetal FCs elongated with normal timing, but extended side-by-side (yellow arrow, left), compared with top wild-type FCs (blue arrow, left). (D) Ingression of a single wild-type FC adjacent to about four GFP^+^ leaders and followers (blue arrow), appears relatively normal, compared with wild-type leaders below (yellow arrow). (E) Thinning basal regions of the top three wild-type FCs was associated with a ‘vertically stacked’ organization of three FC nuclei (blue arrow). At the bottom, the first two GFP^+^ leader FCs have visible GFP^+^ nuclei (yellow arrow, left), but the third nucleus is from basal intermingling of an adjacent GFP^−^ FC. (F) Weakly GFP^+^ leading and following FCs were associated with variably delayed thinning of basal contact for the adjacent two wild-type leading FCs. Wild-type leading FCs exhibited elongation with abnormal nuclear arrangement (blue arrow, left). Second leading FC nucleus was abnormally basal. Third leading FC was GFP^+^, but this weakly-depleted FC showed an apically shifted nucleus. The fourth FC was GFP^+^ and exhibited an intermediate morphology with an apically shifted nucleus, and a curved elongated shape. Scale bars: 25 µm.

Control GFP^+^ clone centripetal FCs exhibited normal morphology and progression of centripetal milestones through Milestone III, and in four cases to Milestone V (*n*=35; Fig. 5A,B). Movement of a single GFP^+^ ingressing leader FC at Milestone III highlighted its spindle-like shape (Fig. S7). In contrast, presence of one or more GFP^+^ E-cadherin knockdown leading FCs resulted in delayed progression from Milestone II to Milestone III (GFP^+^ leaders only: median=273.9 min, *n*=8, *P*=0.0047; both GFP^+^ leaders and followers: median=252.1 min, *n*=7, *P*=0.0074; GFP^+^ followers only: median=220.8 min, *n*=7, *P*=0.7767; Fig. S8). A clone that included both GFP^+^ leading and following centripetal FCs is shown (Fig. 5C; Movie 3).

We assessed basal thinning and apical elongation between Milestones II and III for leading FC1 and leading FC2, by pooling all samples with one or more GFP^+^ leading FCs. For either of the first two leading FCs, basal thinning was significantly reduced (E-cadherin-depleted leader 1 median change=0.94 µm, *n*=22, *P*=0.0002; E-cadherin-depleted leader 2 median change=1.29 µm, *n*=22, *P*=0.0175; Figs S9, S10; Movie 4). They continued apical extension, with leading FC1 showing a significant increase (leader 1 median change=19.6 µm, *n*=13, *P*=0.0188; leader 2 median change=11.5 µm, *n*=22, *P*=0.1775; Fig. 5E; Fig. S11). For both metrics, leader FCs showed no significant difference between *shg* and control RNAi in the GFP^+^ followers only, a smaller dataset comparable with the smaller GFP^+^ leaders only and GFP^+^ leaders and followers datasets (Figs S12, S13). However, GFP^−^ wild-type leading FCs with GFP^+^ followers occasionally showed aberrant nuclear location relative to the opposite wild-type leading FCs (compare wild-type leaders next to GFP^+^ followers, blue arrows in Fig. 5D,F, to opposite leaders in Fig. 5D, yellow arrow, and Fig. 5E, blue arrow).

In sum, E-cadherin-depleted leading centripetal FCs showed delayed progression from Milestone II to Milestone III, reflected by a decreased basal thinning overall, whether or not Milestone III occurred before the end of the time-lapse sequence. In samples with GFP^+^ following FCs, the GFP-leading FCs ingressed, completing Milestone III for that first cell (4 of *n*=4; Fig. 5B). Surprisingly, E-cadherin-depleted leading FCs could extend apically, and continued extending over a prolonged Milestone II to Milestone III interval.

Full transition to Milestone V occurred in only three control samples, an additional sample had started Milestone V (*n*=28). Milestone V occurred in one E-cadherin-depleted sample (*n*=32; Fig. S14). As a proxy for progression to Milestone V, we assessed movement of following FCs toward the anterior by measuring displacement of nuclei from beginning to end of each time-lapse sequence. No significant difference was observed between *shg* and control RNAi for any of the three sample classes (Fig. S15); this analysis was impacted by clone locations above or below the sample midline, and by increasing oocyte volume (see Materials and Methods).

**Fig. 6.**
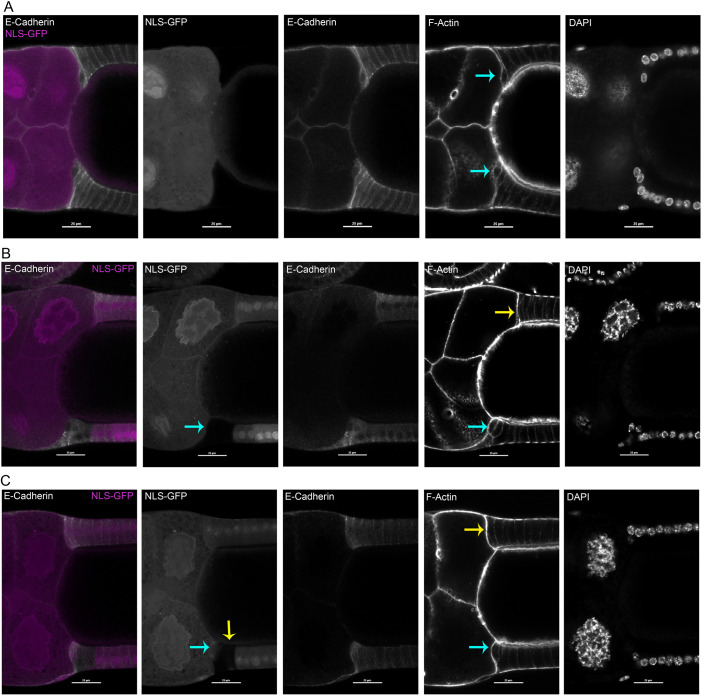
**Homozygous *shg^2^* mutant FCs disrupted early stages of centripetal migration.** (A-C) Fixed egg chambers containing *shg^+^* homozygous control clone (A) or *shg^2^* homozygous mutant clones (B,C) in or near leading FCs, in a background of nuclear GFP^+^ heterozygous FCs. Each row shows separated fluors from the same sample. Homozygous mitotic clones were marked with reduced NLS::GFP levels for both s*hg*^2^ clones or control *shg^+^* clones. Left panels show merge of GFP (magenta, in both nuclei and cytoplasm) with anti-E-cadherin staining (white). Cell morphology was visualized with Phalloidin to detect F-actin; nuclei detected with DAPI (right only). (A) Large control clone, encompassing all FCs at top and bottom, exhibited normal E-cadherin localization and relatively normal leading FCs near the transition to Milestone IV (blue arrows). (B) Abnormally shaped, wild-type leading FCs (blue arrow, bottom) were adjacent to homozygous mutant *shg^2^* following FCs, compared with leading FCs with wild-type posterior neighbors (yellow arrow, top). (C) Three *shg^2^* homozygous leading FCs (yellow arrow, NLS-GFP panel) appeared to bunch together; two have reduced contact with basement membrane prematurely (blue arrow, NLS-GFP and F-Actin panels). Opposite wild-type leading FCs may have started Milestone I, but elongation is minimal (yellow arrow, top in F-Actin panel). This sample suggests that aberrant rounding of leading FCs may initiate before Milestone I. Scale bars: 25 µm.

We next examined phenotypes associated with FCs homozygous for either *shg^1^* or *shg^2^* (Oda et al., 1997; Tearle and Nusslein-Volhard, 1987; Tepass et al., 1996; Uemura et al., 1996). This experiment used Flp-FRT-mediated chromosomal (mitotic) recombination to generate homozygous mutant cells in a heterozygous tissue (Pignoni and Zipursky, 1997; Xu and Rubin, 1993). For this experiment, we examined only fixed immunostained egg chambers, because we lacked suitable fluorescent protein markers to use with FRT-G13. GFP^−^ homozygous mutant cells (*shg^−/−^*^)^ were induced in a GFP^+^ heterozygous background, and GFP^−^ *shg^+^* control clones induced in parallel experiments. Egg chambers with GFP^+^ FCs in or near leading centripetal FCs were examined. Egg chambers with control GFP^+^ *shg^+/+^* clones exhibited normal centripetal FC organization and represented snapshots of multiple milestones (41 of *n*=41; Fig. 6A).

*shg^−/−^* centripetal FCs exhibited both autonomous and non-autonomous phenotypes. Mutant *shg* leading FCs were aberrantly rounded and clustered, suggesting failed ingression (25 of *n*=25; Fig. 6B). Conversely, when *shg^−/−^* FCs were posterior neighbors to two *shg^+^* leading centripetal FCs, the *shg^+^* leading FCs were clustered together with rounded morphology (7 of *n*=7; Fig. 6C). Some egg chambers had multiple *shg^+^* leading and following FCs, with *shg^−/−^* posterior following/mainbody FCs; apparently normal morphologies of Milestones V-VII were seen in those lacking extensive clones elsewhere in the egg chamber (4 of *n*=4).

Rounded, clustered *shg^−/−^* leading FCs in the epithelium of fixed egg chambers may occur more frequently from greater loss of E-cadherin in mutant FCs than in RNAi-depleted FCs. We observed two *shg* knockdown samples with similar clustering of rounded GFP^+^ leading centripetal FC samples with both GFP^+^ leader and follower FCs (*n*=13), and none in that class of control samples (*n*=8). Alternatively, the rounded clustered phenotype could occur more frequently in the presence of reduced E-cadherin levels in adjacent *shg^+/−^* FCs. Lastly, the phenotype could be exacerbated by fixation and processing for immunofluorescence.

Altogether, our data indicate that leading FCs require E-cadherin autonomously to ingress. Basal thinning was more sensitive to decreased levels of E-cadherin than was apical extension. Loss-of-function mitotic clones suggest that apical extension requires some minimum level of E-cadherin, or may be influenced by lower levels of E-cadherin in more posterior FCs.

### Germ cell expression of E-cadherin influences speed and direction of centripetal migration

The requirement for E-cadherin in germ cells could reflect a direct requirement for E-cadherin adhesion between centripetal FCs and germ cells (Niewiadomska et al., 1999; Oda et al., 1997; Tepass et al., 1996). Alternatively, germ cell-germ cell adhesion might indirectly affect centripetal migration, as seen for border cell migration (Cai et al., 2014).

We used the flipout-Gal4 system to both express GFP and activate RNAi in a few germ cells within a mosaic egg chamber (E-cadherin and control depleted germ cell movies compiled in Movie 5). Control clones expressed *luciferase* dsRNA. Wild-type leading FCs progressed normally when control clones included adjacent nurse cells (12 of *n*=12; Fig. S16A). Two *shg* dsRNA transgenes were used. When germ cells had weak *shg* knockdown, mild aberrations were observed (7 of *n*=7; Fig. S16B).

Strongly E-cadherin-depleted nurse cells were associated with altered germ cell organization and with aberrant morphology of centripetal FCs and later anterior epithelium. These knockdown nurse cells were abnormally rounded, with adjacent wild-type centripetal FCs appearing to conform to nurse cell shape (Fig. S16C, yellow arrow). In ∼60% of samples with E-cadherin-depleted nurse cells, the oocyte curved outwards into the nurse cell compartment (11 of *n*=18; Fig. S16C, left); in over half, elongating leading centripetal FCs abutted the bulging oocyte (7 of *n*=11; Fig. S16C, right).

With early, strong germ cell *shg* knockdown, a few egg chambers had a mispositioned oocyte (3 of *n*=34; Fig. S16D), due to the E-cadherin requirement for posterior oocyte position (Godt and Tepass, 1998; González-Reyes and St Johnston, 1998; Niewiadomska et al., 1999). Such oocytes had elongating centripetal FCs on both anterior and posterior sides, but progression to Milestone V was not observed, consistent with previous reports (González-Reyes et al., 1995; González-Reyes and St Johnston, 1998; Peri et al., 1999; Peri and Roth, 2000).

**Fig. 7.**
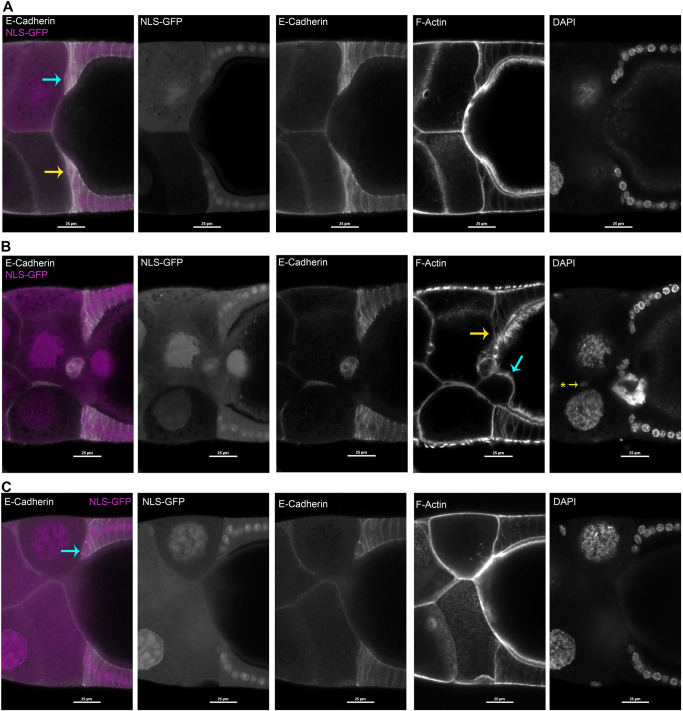
**Abnormal centripetal migration of wild-type FCs adjacent to homozygous s*hg*^1^ mutant nurse cells.** (A-C) Fixed egg chambers containing germ cell clones, either *shg^+^* homozygous control (A) or *shg*^1^ homozygous mutant clones (B,C), in or near centripetal FCs, in a background of GFP^+^ heterozygous FCs. Each row shows separated fluors from same image. Homozygous mitotic clones were marked with reduced GFP levels for s*hg*^1^ clones or control *shg^+^* clones. Left panel: merged GFP (magenta, in both nuclei and cytoplasm) with anti-E-cadherin (white); remaining panels, each alone. Right two panels visualize cell morphology with Phalloidin (F-actin) and DAPI (nuclei). (A) E-cadherin localization and leading FC morphology were similar for leading FCs adjacent to a GFP^−^ control nurse cell (yellow arrow, bottom left) and for leading FCs adjacent to GFP^+^ wild-type nurse cells (blue arrow, top left). (B) In some samples, *shg*^1^ homozygous mutant nurse cells abnormally protruded into the surface of the oocyte (blue arrow, F-actin panel). Leading FCs abutted the displaced nurse cell, with apparently blocked migration (bottom) compared with opposite leading FCs (yellow arrow, F-actin panel). (C) When *shg*^1^ homozygous mutant nurse cells were normally organized, adjacent *shg^+^* leading FCs had moved inward (blue arrow, left; compare with Fig. 2D) but lacked the elongated morphology of Milestone II-III leading FCs on the opposite side (bottom). Scale bars: 25 µm.

To examine organization of *shg^+^* leading centripetal FCs adjacent to a *shg^−/−^* germ cell, we used the same two *shg* alleles, with immunostained fixed egg chambers. Mosaic egg chambers had a mix of homozygous wild-type and homozygous mutant germ cells and FCs. We excluded egg chambers that contained mutant FC clones within or near centripetal FCs. Egg chambers with control *shg*^+/+^ posterior nurse cell clones exhibited normal leader centripetal FC organization, providing snapshots through Milestone III (9 of *n*=9; Fig. 7A). When *shg^−/−^* nurse cells were adjacent to *shg^+^* leading centripetal FCs, we observed two distinct leading FC phenotypes. In some samples, FCs had elongated along the *shg^−/−^* nurse cell, but abutted an interior *shg^+^* nurse cell (7 of *n*=7; Fig. 7B, blue arrow). In other samples, leading centripetal FCs exhibited disordered morphology in Milestone III-V (18 of *n*=18; Fig. 7C). Altogether, *shg^−/−^* nurse cell organization non-autonomously influenced organization of ingressing FCs, and could block migration during Milestones III-V.

Finally, germ cell RNAi samples provided an opportunity to examine centripetal migration with mis-positioned border cells, the migration of which is influenced by nurse cell E-cadherin levels (Cai et al., 2014; Dai et al., 2020). In some samples with E-cadherin-depleted nurse cells, border cells stayed among nurse cells, but wild-type centripetal FCs migrated normally (11 of *n*=34; Fig. S6D). Thus, centripetal migration occurs independently from presence of border cells at the oocyte:nurse cell interface.

## DISCUSSION

Centripetal migration is essential to enclose the *Drosophila* oocyte in an eggshell to protect the externally developing embryo. Other FC migrations have contributed important insights into collective cell migration. However, centripetal migration is distinct from other FC migrations: circumferential migration of the FC epithelium (Haigo and Bilder, 2011; Cetera and Horne-Badovinac, 2015), border cell migration (King, 1970; Montell, 2003; Saadin and Starz-Gaiano, 2016) and tube formation for dorsal appendages (Ward and Berg, 2005). Our work defined a series of reproducible milestones for centripetal migration and distinguished two overall phases.

Centripetal migration appears to vary across insects; for example, lower brachycerans exhibit cellular rearrangements similar to infolding (Garbiec and Kubrakiewicz, 2012; Garbiec et al., 2016; Tworzydlo and Kisiel, 2011). We assessed whether *D. melanogaster* centripetal migration might involve infolding of squamous stretch FCs and columnar FCs, as suggested by Tran and Berg (2003). Alternative mechanisms are epithelial-mesenchymal transition and delamination.

Three rings of anterior columnar centripetal FCs undergo apical elongation and basal thinning, then move inward from the basement membrane. This morphology is unlike infolding, and apically-directed migration is distinct from epithelial-mesenchymal transition (Keller and Shook, 2011; Pearl et al., 2017). Of the two mechanisms for delamination, extrusion and ingression, early centripetal migration appears more like ingression (Niewiadomska et al., 1999; Oda et al., 1997; Pasiliao and Hopyan, 2018).

As an essential step towards elucidating centripetal migration, we defined a timeline of morphological milestones. Overall, the sequence of milestones indicated ingression of three rings of leading columnar FCs (Milestones I-III), which transitioned to epithelial migration for following posterior FCs, which maintain their FC:oocyte and FC:FC interfaces as they round the oocyte corner to move inward, beginning at Milestone V. These distinct migration morphologies suggested two distinct phases of migration: first, ingression of leading centripetal FCs, and second, collective epithelial migration of following centripetal FCs. Consistent with this distinction, leading centripetal FCs show a distinct gene expression profile (Deng and Bownes, 1997; Levine et al., 2007; Yakoby et al., 2008a,b), suggesting a separate gene regulatory network from following centripetal FCs.

Our timeline will help pinpoint functions for genes required in centripetal migration. A handful of such genes are known, but few have been investigated. Most notably, the E-cadherin gene, *shg*, is required in both FCs and germ cells for normal migration. E-cadherin is expressed in all cells of egg chambers; leading FCs accumulate higher E-cadherin levels along their lateral faces (Niewiadomska et al., 1999). We selected this gene to test the utility of our framework. Time-lapse imaging of mosaic egg chambers with clones of E-cadherin-depleted FCs was used to obtain temporal resolution for the requirements in leading FCs, following FCs, and germ cells. Observations were compared with morphologies of fixed egg chambers with clones of homozygous null *shg^−/−^* FCs or germ cells.

We first examined time-lapse sequences for FCs with *in vivo* RNAi. E-cadherin depletion in leading FCs were associated with significant delays in progression from Milestone II to III, accompanied by continued apical extension, even though basal thinning was delayed. Significant delays at Milestone III support the proposal that E-cadherin is important to retract the trailing basal edge of centripetal FCs (Niewiadomska et al., 1999). Wild-type following FCs were not observed to migrate around E-cadherin-depleted leading FCs.

For *shg* mitotic clone analysis, we examined immunostained fixed egg chambers, due to paucity of suitable fluorescent protein tools. One distinction for mitotic clones was *shg^−/−^* leading FCs’ consistently rounded and clustered appearance (Fig. 6C). *shg* knockdown leading FCs were rounded in only three samples. *shg^−/−^* following FCs appeared to non-autonomously impact leading FC ingression. Although fixed tissue images are snap shots at different milestones, observations supported a failure to progress to Milestone V. Mitotic clones were consistent with RNAi results, where leading FCs autonomously required E-cadherin to complete Phase I ingression.

E-cadherin-mediated adhesion requires E-cadherin accumulation on both apposed surfaces of neighboring cells, suggesting that non-autonomous effects are likely. However, our small sample size precluded precision analysis of non-autonomy for single neighboring FCs.

Germ cell knockdown by RNAi was associated with altered germ cell morphology, precluding evaluation of Milestones IV-VII. *shg^+^* leading FCs adjacent to *shg^−/−^* nurse cells exhibited more severe Milestone III defects than those adjacent to E-cadherin knockdown nurse cells. Progression of leading FC migration followed normal timing through Milestone III, but FCs conformed to the rounded surface of an adjacent E-cadherin-depleted nurse cells. In ∼60% of samples, depleted nurse cells’ abnormal morphology was associated with an anterior-bulging oocyte. For about half these samples, wild-type leading FCs elongated to abut the oocyte surface and stalled. In a few samples with mispositioned nurse cells, wild-type centripetal FCs moved inward, but stalled when they abutted an aberrant nurse cell. Overall, these results suggest that leading FCs follow guidance cues associated with adjacent nurse cell morphology, and may require a relatively linear path to continue migration through Milestone V.

Leading centripetal FCs have elevated and extended E-cadherin localization along their lateral faces as they migrate (Fig. 6; Niewiadomska et al., 1999). This might support traction along adjacent FC:FC interfaces for ingression, and/or support increased stiffness of the elongating FCs by organizing cortical actinomyosin cytoskeleton along FC:FC interfaces. Migrating astrocyte clusters exhibit actin-dependent N-cadherin treadmilling at lateral interfaces during collective migration; similar N-cadherin dependence is seen for neuronal growth cone migration (Garcia et al., 2015; Peglion et al., 2014). Leading centripetal FCs provide a distinct epithelial system for investigations of Cadherin adhesion-mediated migration.

The guidance mechanism for centripetal migration has been a lingering question. A chemotactic signal from border cells was one speculated source. We found that centripetal FCs migrated independently of correctly positioned border cells, confirming early observations from laser ablation (Montell et al., 1991). Thus, substrate-dependent guidance becomes an attractive mechanism, supported by phenotypes of germ cells with low or no E-cadherin.

Reduced nurse cell E-cadherin influenced organization of germ cells within egg chambers, and also morphology of migrating *shg^+^* FCs during Milestones I-V. Specific phenotypes appeared to depend on location of affected nurse cells. Leading centripetal FCs could ingress adjacent to a depleted or mutant nurse cell if the path of the FC was unobstructed. However, if germ cell disorganization was associated with an anteriorly bulged oocyte, or a nurse cell pressing into the oocyte, migrating FCs abutted the misplaced germ cell and stalled. These data suggest that E-cadherin in germ cells may ensure an unobstructed path for initial ingression, resembling topotactic guidance (Dai et al., 2020; reviewed by SenGupta et al., 2021).

Whether leading FC ingression is coordinated by a supracellular actomyosin ring at the leading edge (Edwards and Kiehart, 1996) is an open question. This actinomyosin ring may draw leading FCs inward or provide resistance to pressure from surrounding germ cells (Aranjuez et al., 2016). If important, this actinomyosin ring must accommodate disrupted migration of a few FCs within a circumferential ring, because adjacent leading FCs continue inward, as seen for *shg* (Figs 5E and 6B; T.T.P., unpublished observations) and *cut* mutant FCs (Levine et al., 2010).

Altogether, our data support an initial phase of leading FC ingression, which initiates or potentiates collective migration of following FCs. Our milestones provide a sketch of this process, with open questions about finer-scale events. Presumptive centripetal FCs, which coincide with leading FCs, exhibit distinct gene expression (Chen and Schüpbach, 2006; Dobens et al., 2005; Fauré et al., 2014; Fregoso Lomas et al., 2016; Shravage et al., 2007; Xi et al., 2003). Reported expression include responses to BMP and Ecdysone signaling, possibly also JAK-Stat activity (Buszczak et al., 1999; Carney and Bender, 2000; Deng and Bownes, 1997; Dobens et al., 2000; Domanitskaya et al., 2014; Freeman et al., 1999; Hackney et al., 2007; Kozlova and Thummel, 2000; Levine et al., 2010; Romani et al., 2009, 2016; Sieber and Spradling, 2015). Whether these signals regulate ingression is unknown.

Our methodology for live imaging and timeline of milestones make centripetal migration an accessible system to genetically dissect mechanisms for both leading FC ingression and epithelial migration of following FCs. Centripetal FCs undertake migration after secretion has begun, whereas the majority of secretory FCs remain at the surface. Thus, they provide a new system to investigate plasticity of secretory epithelia. We expect that our foundational data will provide a platform to determine how a differentiated secretory epithelium can undergo localized migration, and then restore secretory function.

## MATERIALS AND METHODS

### *Drosophila* strains, genotypes, and mating schemes

Table S1 includes the strains used in this work and their sources. Table S2 provides the genotypes for each figure panel and Movie. GFP-Collagen IV was expressed from *Vkg-GFP[CC00791]* (Buszczak et al., 2007; Medioni and Noselli, 2005). Membrane markers were expressed from transgenes: myristoylated tdTomato (Myr.tdTomato) from *P{w[+mC], 10XUAS-IVS-myr::tdTomato}*, myristoylated-tdEOS (myr::tdEOS) from a *P{y[+t7.7] w[+mC]=10XUAS-IVS-myr::tdEos}* and myristoylated-GFP (myr::GFP) from *P{10XUAS−IVS−myr::GFP}* (Pfeiffer et al., 2010). Double-stranded RNA for RNAi of *shg* was expressed in follicle cells from TRiP HMS00693 or TRiP JF02769, or in germ cells from TRiP GL00646. Control dsRNA to target luciferase was expressed from TRiP JF01355. Clonal expression of UAS-transgenes was achieved using Flipout Gal4 strain, *P{w[+mc}, Act5C>CD2>Gal4} P{w[+mC], UAS-GFP}* (Pignoni and Zipursky, 1997). The Flipout Gal4 and hsFlp transgenes were maintained in separate strains and brought together in matings between males and females carrying combinations of the needed genetic components. For each experiment, females bearing all the necessary genetic components were generated by mating and selected by screening for associated visible markers. Stable fly strains generated for this work are available upon request.

### *Drosophila* culture and conditioning to promote egg production

All flies were reared at 25°C, 60% relative humidity on a cornmeal/soy flour/agar/yeast/corn syrup medium. Unless stated otherwise, mated male and female flies were conditioned for 2 days before dissections in vials containing freshly prepared yeast paste. For experiments involving inactivation of Gal80[TS], flies were reared at 25°C, then shifted to 29°C 2 days before dissection.

For Flp-FRT-mediated chromosomal recombination to generate somatic and germ cell homozygous mutant clones, 2- to 4-day old adult females were heat shocked at 36.7-37°C for ∼2 h twice within 2 days and dissected 2 days after heat shock for FC clones, and 8 days after the first heat shock for germ cell clones, to recover clones.

### RNAi-mediated knockdown in mosaic egg chambers

For RNAi, flipout clones were generated to express a dsRNA transgene (Pignoni and Zipursky, 1997). Transgenes were from the *Drosophila* RNAi Screening Center (DRSC), generated by the Transgenic RNAi Project (Perkins et al., 2015). These transgenes were generated using VALIUM10/20/22 vectors designed for efficient somatic and/or germ cell expression (Ni et al., 2009, 2008, 2011). FC clones were generated in mated 2- to 4-day old adult females, using a 37°C heat shock for 45 min. The flies were returned to 25°C and dissected 2 days later. For germ cell RNAi, females were dissected 7 days after the same heat shock regimen. Clones expressing dsRNAs were marked by co-expression of UAS-GFP. E-cadherin staining suggests that somatic cell knockdown with TRiP JF02769 was stronger than with TRiP HMS00693 (Compare Fig. S6E with Fig. S6C). Specific strains used are listed in Table S1.

### *Ex vivo* culture and time-lapse imaging

Our *ex vivo* culture protocol was adapted from previous work (Cetera et al., 2016; Prasad et al., 2007). Briefly, egg chambers were dissected from ovaries in a culture medium containing Schneider's *Drosophila* Medium (Thermo Fisher Scientific) supplemented with 15% v/v FBS (Thermo Fisher Scientific), 0.6× pen/strep (Thermo Fisher Scientific) and 200 µg/ml insulin (Sigma-Aldrich), then cultured on Lumox dishes (Sarstedt) in the same medium supplemented with 0.8% agarose (Sigma-Aldrich).

For each imaging session, four or five conditioned females were dissected in a nine-well glass plate (Corning 722085) in freshly prepared live imaging medium. Ovarioles were gently teased apart. Later stage egg chambers (stages 11-14) were removed from the dish, remaining egg chambers transferred in a total volume of 50 µl to a coverslip lined with double-sided film tape (The 3M Company, S-15941), and a Lumox dish was lowered over the medium. Egg chambers were cultured for ∼5 h under the microscope, with ∼10-13 optical sections obtained about every 8-10 min. In creating the migration timeline, timings and durations were compiled from overlapping sequences of multiple egg chambers developing over time. To capture centripetally migrating cells a midline section of each egg chamber was imaged, as identified from the distance from the top of the egg chamber in combination with visual identification of the widest diameter of the tissue.

### Photoconversion of tdEOS

When photoconversion was used to label a group of cells, the region of interest was selected immediately before the start of the time-lapse. Using the 60× objective of the Nikon A1R confocal laser scanning microscope, the region was exposed to pulses of a 405 nm light at a laser power of ∼8 using a Coherent OBIS 408 nm 100 mW laser, for ∼15-30 s. Following photoconversion the automated time-lapse imaging session was initiated.

### Selection and testing of stretch FC-specific Gal4 transgenes

A90-Gal4 (Yeh et al., 1995), C415-Gal4 (Manseau et al., 1997) and PG150-Gal4 (Bourbon et al., 2002) were used to label stretch FCs. For each driver, reporter expression level varied between individual stretch FCs, with a different pattern in each egg chamber (Fig. S4A-C). For A90-Gal4, weak reporter expression was detectable in the first one to two leading columnar FCs when using either cytoplasmic or nuclear localized fluorescent proteins. In contrast, fluorescence from a myristoylated fluorescent protein was detected in the stretch FCs alone (Fig. S4E-H). As maximal labeling of stretch FCs was important for determining whether they extended in tandem with FCs, we evaluated stretch FC behavior for each of the three transgenes to minimize the impact of variations in expression level. In addition, a pan-follicle cell driver combination (tj-Gal4 mef2-Gal80, ‘TJ’; Andersen and Horne-Badovinac, 2016) (Fig. S4D) was used in conjunction with photoconversion to label stretch FCs manually. A90 and TJ>>EOS provided the most reliable fluorescent labeling, with C415 and PG150 providing the least reliable (Fig. 4E,F).

### High resolution optical sections for volumetric projection

To determine whether stretch FCs extended inward in tandem with centripetal cells, a large set of optical sections were obtained at the end of time-lapse sessions. Using a 1024×1024 pixel resolution 15 frames per second resonant scanner with a large field of view, 300 to 350 optical sections were rapidly acquired at a spacing of ∼0.33-0.5 µm. Subsequently, these optical sections were used to render volumetric projections of inwardly extending stretch FCs using the Nikon NIS Elements software (v4.2). Bounding boxes with 3D scales are not shown due to level of crop and zoom. Instead, scale bars from midline *xy* optical images were used to calculate scale for volumetric projections, using the measured midline diameter of egg chamber in pixels for *xy* and *xyz* images.

### Antibodies and actin labeling

Primary antibodies used are as follows: 1:500 concentrated monoclonal rat anti-Shotgun DCAD2 (Developmental Studies Hybridoma Bank, deposited by T. Uemura, Kyoto University, Japan) and 1:200 rabbit polyclonal anti-GFP (Thermo Fisher Scientific, A-11122). The following secondary antibodies were used at a dilution of 1:500: polyclonal goat anti-rabbit Alexa 488 (Thermo Fisher Scientific, A-11008), polyclonal goat anti-mouse Alexa 568 (Thermo Fisher Scientific, A-11004) and polyclonal goat anti-rat cyaninine5 (Thermo Fisher Scientific, A-10525). For F-actin staining, Phalloidin-TRITC (Sigma-Aldrich, P1951) was used at 1:2500 during fixation and 1:500 during post-fix washes. For more information, see Table S1.

### Immunofluorescent staining

To immunofluorescently label egg chambers, conditioned ovaries were dissected in PBS (1 mM KH_2_PO_4_, 155 mM NaCl, 3 mM Na_2_HPO_4_, pH 7.4) or live imaging medium and fixed with 4% formaldehyde in PBS for 20 min, followed by 10 min washes with 1× PBS supplemented with 0.2% Triton X-100 (PBT). In experiments with labeled filamentous actin, phalloidin-TRITC was added to fixative to make a dilution of 1:2500, followed by two 20 min washes at a dilution of 1:500 in PBT (adapted from Frydman and Spradling, 2001). After washing, blocking was carried out with 8% FBS in 1× PBT for 1 h, after which egg chambers were incubated with primary antibodies added to a blocking solution of 5% FBS solution in 1× PBT for ∼12-16 h at 4°C. After washing off primary antibodies with 1× PBT (three washes, 10 min each), egg chambers were incubated with secondary antibodies added to a blocking solution of 5% FBS solution in 1× PBT for ∼2 h at room temperature. After two washes with 1× PBT for 10 min each, DAPI stain (1 µg/ml) was applied for 5 min before a final wash with 1× PBT. Egg chambers were mounted in Vectashield (Vector Labs) on slides with a #1.5 coverslip (Warner Instruments, 64-0721). During imaging of mosaic egg chambers, those that appeared damaged in brightfield or exhibited simultaneous loss of DAPI/Phalloidin/GFP/Cadherin were excluded from subsequent analysis due to the possibility of containing ‘false clones’, as described in Haack et al. (2013).

### Microscopy, milestone analysis and software

Live time-lapse images and immunofluorescent micrographs were acquired on a Nikon A1R confocal laser scanning microscope, as well as a Nikon Ni-E multiphoton laser scanning microscope operating in confocal mode with visible lasers. For microscope objective information, see Table S1.

For initial milestone analysis, measurements of basal width used midline optical sections with anterior to left. Width was measured at the space between the lateral FC:FC interfaces, as visualized with Myr::tdTomato. Apical-basal length in columnar FCs (sometimes called height) is greater for the dorsal-most FCs than the ventral-most FCs (Spradling, 1993; Duhart et al., 2017); our samples varied in their dorsal-ventral orientation, but subtraction of Milestone II length from the Milestone III length eliminated any variation from dorsal-ventral orientation at Milestone II.

For preparation of magnified images in Fig. 2 and Fig. S7 full resolution images were exported from NIS Elements and scaled down to fit. Scale bars were estimated based on image pixel data. Analysis, figure and movie preparation was performed in NIS Elements AR v4.2 and v5.30.05, GraphPad Prism 7 and 9.4.0, HandBrake 1.1.1, Adobe Photoshop CS6 and Adobe Premiere Pro CC 2015.

### Selection of FC RNAi clones and analysis

Flp-FRT-induced clones expressing hairpin RNAs for *shg* RNAi analysis occurred randomly within the follicular epithelium, and varied in size. Cortical staining of FCs was obtained from expression of Sqh::mCherry (myosin type 2 regulatory light chain, C-terminally tagged with mCherry). Clones were selected for analysis if they occurred in the anterior region of the columnar epithelium, at or near the midline optical section (± ∼20 µm), and the egg chamber was between Milestone I and Milestone III, although a few had passed Milestone III when the first optical section was collected. Clones were followed across *z*-sections as an egg chamber drifted over time.

Samples were categorized according to the location of GFP^+^ FCs as follows. GFP^+^ leaders only: counting from the most anterior FC, these clones included those where the first leading FC, the first two leading FC, or all three leading FCs were GFP^+^, but all more posterior FCs (follower and mainbody) were GFP^−^. Followers only: these samples always included GFP^−^ first leader FC, or GFP^−^ first two leader FCs, and GFP^+^ FCs at positions 4 and 5, also frequently GFP^+^ cells at position 6 and more posterior. Some also included GFP^+^ leader 3. Both GFP^+^ leaders and followers: these samples generally included GFP^+^ FCs in positions 1 through 5, and beyond. A few samples had intermingled GFP^+^ and GFP^−^ FCs.

Measurements were performed in in separate analyses for Milestone II and III, versus Milestone V and following cell displacement. Samples that had passed Milestone II were excluded from Milestone III analyses, whereas measurements were taken at the end of the time-lapse sequence for samples that did not reach Milestone III. Otherwise, the measurements of changes in basal width and apical-basal length were performed in the same way as for the initial milestone analysis (NIS Elements AR v4.2 and v5.30.05).

In both *shg* and control RNAi samples, some failed to reach Milestone III before the time-lapse sequence ended: samples that did not reach Milestone III occurred in every data set: GFP^+^ leaders only: *shg* knockdown – 4 of *n*=8 and control 1 of *n*=6. Both GFP^+^ leaders and followers: *shg* knockdown – 4 of *n*=9 and control 1 of *n*=7. GFP^+^ followers only: *shg* knockdown – 3 of *n*=7 and control 3 of *n*=9. For statistical analyses of these samples where the Milestone II-Milestone III interval was measured as greater than a specific time, we added 30 min to each calculated interval as a crude estimate for the Mann–Whitney test of significance, representing an interval of four to five frames of a time-lapse sequence.

Only four control samples reached Milestone V in this experiment (*n*=28), and only one *shg* RNAi sample (*n*= 28). The rare observation of Milestone V may reflect the effects of the previous heat shock treatment to induce flipout clones, the presence of mCherry-tagged myosin type 2 regulatory light chain in a background of wild-type proteins, the activation of RNAi in clones of cells anywhere in the sample or other background effects in this genotype.

Measurements of follower cell displacement relied on investigator identification of nuclei, defined by a weak to absent Sqh::mCherry in the central region of each FC from the beginning of imaging until Milestone V, or the end of the time-lapse series (generally 5-6 h). Nuclei were numbered one to eight from the anterior-most FC at the beginning of the time-lapse sequence, and the distance from the nucleus center to the nurse cell:FC boundary was measured. These nuclei were manually tracked over the entire period, using the presence or absence of GFP to help confirm identification. Displacement was calculated by subtracting the distance at the end from the distance at the beginning. We expected that normal progression to Milestone V would yield a positive value, whereas slow or stalled progression to Milestone V would yield a negative value, owing to FC spreading to accommodate oocyte expansion along the A-P axis (King, 1970; Spradling, 1993; Weichselberger et al., 2022). Confounding variables likely contributed to the variability in these metrics.

Known confounding variables include the following. The magnification used for our live-imaging was too high to encompass the full A-P length of the oocyte, even at the beginning. No other landmark was available to normalize the A-P width of follicle cells over time. Also, the hexagonal packing of the follicular epithelium leads to variation in location of the FC center relative to the slice in the optical section. Finally, FCs are modestly mobile within the epithelium, so that GFP^+^ and GFP^−^ cells occasionally exchanged at the edges of clones over the ∼6 min between each image. We observed this for both follower and mainbody FCs in both control and *shg* knockdown clones.

### Statistics

For most datasets, we selected a non-parametric analysis to assess statistical differences, typically the Mann–Whitney analysis. Ranking statistics could accommodate a dataset in which some measurements were out of range, as previously described (Motulsky, 1995), allowing us to include samples that had not reached Milestone III after the end of the time-lapse. To summarize, the absolute value was unnecessary for a ranking test, so an estimate was made for a reasonable time when Milestone III might have occurred (30 min after the end of the time-lapse sequence, Fig. S8). We set *P*=0.05 as our cut-off for statistical significance in all analyses. In some cases, we grouped all clones with GFP^+^ leader FCs together, to gain sufficient sample size to reliably detect significant differences. In others, we analyzed each of the three sample categories separately, to assess whether a lack of significance in the followers only subset might reflect the low power of the smaller sample sizes. Details are provided in the figure legends. Graphpad Prism 7 or 9.4.0 was used throughout for selection of the statistical analysis, calculations and graphs.

## Supplementary Material

10.1242/develop.200492_sup1 Supplementary information
