# Supplementary material for: Two phases for centripetal migration of Drosophila melanogaster follicle cells: initial ingression followed by epithelial migration
Source: Development. 2023 Mar 30;150(6):dev200492. doi: 10.1242/dev.200492 (PMC10112906; doi:10.1242/dev.200492)
Supplement: Supplementary information [file develop-150-200492-s1.pdf]

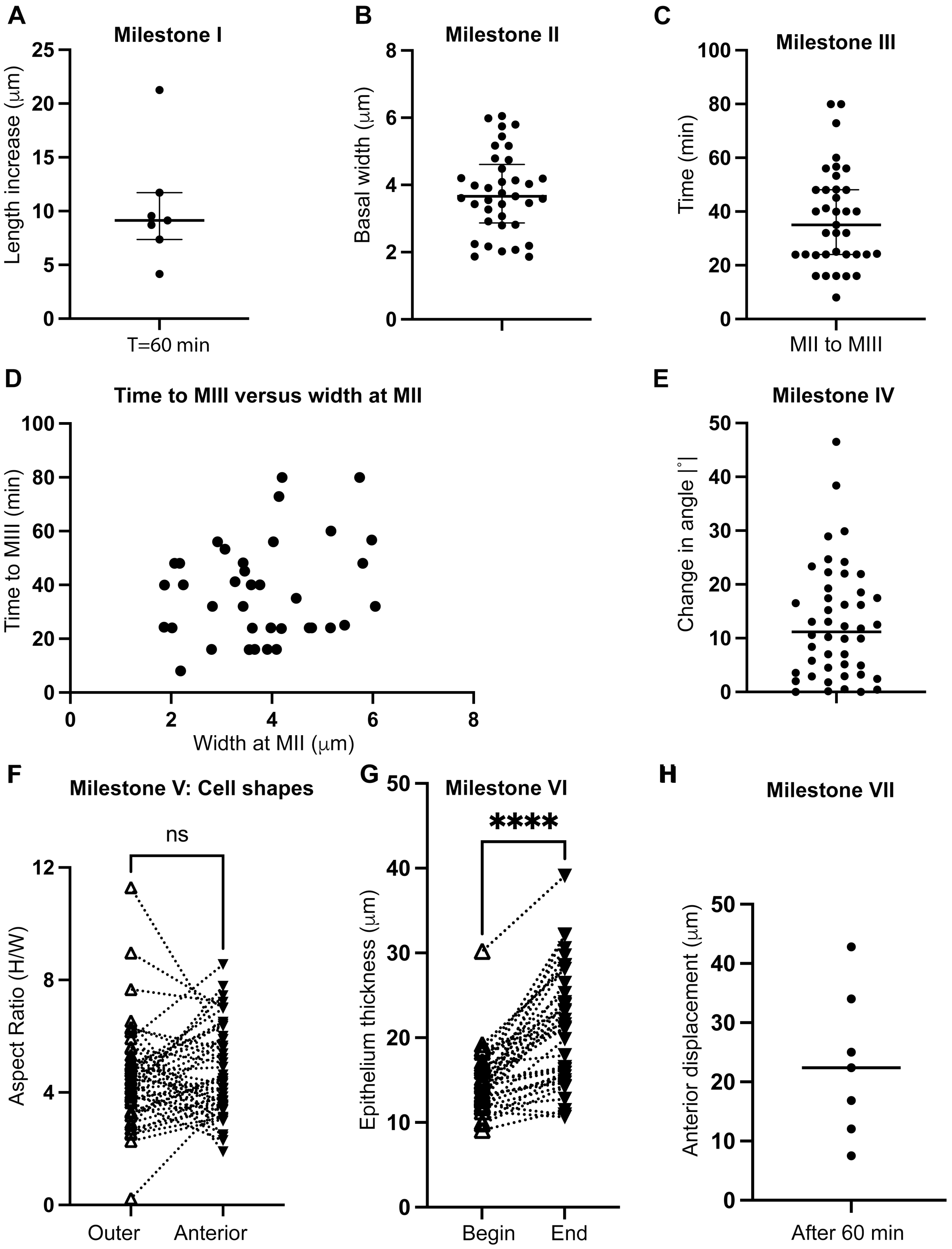
A
Milestone I
Length increase (μm)
25
20
15
10
5
0
T=60 min
B
Milestone II
Basal width (μm)
8
6
4
2
0
C
Milestone III
Time (min)
100
80
60
40
20
0
MII to MIII
D
Time to MIII versus width at MII
Time to MIII (min)
100
80
60
40
20
0
0
2
4
6
8
Width at MII (μm)
E
Milestone IV
Change in angle |°|
50
40
30
20
10
0
F
Milestone V: Cell shapes
ns
Aspect Ratio (H/W)
12
8
4
0
Outer
Anterior
G
Milestone VI
****
Epithelium thickness (μm)
50
40
30
20
10
0
Begin
End
H
Milestone VII
Anterior displacement (μm)
50
40
30
20
10
0
After 60 min

Fig. S1. Dot plots for sample measurements associated with Milestones I – VII. For each panel, median is indicated with a thick horizontal line at the Median, and thin horizontal lines indicating the upper limits of first and third quartiles. A vertical line indicates the InterQuartile Range (IQR). (A) Inward extension of leading FCs. To document the leading FC extension within leading FCs, we measured length of the FC:FC interface in a timepoint just before onset of extension, and in a later timepoint about 60 min later. The difference was calculated and normalized to 60min, for the length increase shown here (See Supplementary Methods). n= 7, Median = 9 μm, IQR = 4 μm. For results section: avg. =10.3, s.d. =1.3. (B) Basal width just before Milestone II. Basal width of first leading centripetal cell was measured just before detectable basal thinning began (visualized by intense Myr::tdTomato in Fig. 2). n= 37, Median = 3.7 μm, IQR 1.7 μm. (C) Time from Milestone II to Milestone III, when basal width is undetectable. In each sample used to measure basal width for (B), we determined the time to Milestone III. This plot represents the same samples represented in Fig 2B. n= 37, Median = 35 min, IQR = 24 min. (D) The time interval to Milestone III is not correlated with the basal width just prior to Milestone II. The time intervals from (C) were plotted on the Y axis, as a function of the starting width from (B), on the X axis. Correlation was determined with Spearman r analysis. Two-tailed P is ~0.3363, indicating no significant correlation. (E) The change in angle at Milestone IV: Angles were measure at top and bottom of egg chambers for each sample, so we used the absolute value of each angle to calculate changes in degrees (|˚|). n= 46, Median = 11˚, IQR =15.2. We used the Wilcoxon signed rank test, using the method of Prat to compare these data to “no change” =0. Two-tailed exact P = <0.0001, indicating a significant change in angle. (F) Milestone V change in migration. Cell aspect ratios were used to evaluate cell shape changes during the shift to inward migration in Milestone V. As each following cell moved around the corner at the junction between the outer epithelium, and the inner, anterior epithelium, they appeared to resume the same ratio of FC:FC interface length (Height) to width. Multiple cells were measured from samples imaged in seven experiments. Height and width were measured in an image just before each cell moved over the corner, and in a later image when it resumed a similar shape as it moved inward along the anterior epithelium (Fig. 2D). Paired outer (before) and

Development • Supplementary information

inner anterior (after) aspect ratios (Height:Width) for each cell are shown, each linked with a dotted line between each pair. n= 46 pairs, Outer epithelium (before) Median ratio = 4.3, Inner Anterior (after) Median ratio = 4.6. The Wilcoxon matched pairs test was used; two-tailed P = 0.3246, indicating no significant change in aspect ratio. (G) Milestone VI thickening of the anterior epithelium. The A – P width of the anterior epithelium was measured at the onset of Milestone VI for each sample, and again in an image about 60 min later. The second timepoints were normalized to 60 min (Paired T0 andT60 epithelium widths are shown linked with a dotted line. n= 36 pairs, Median width at T0 =14 μm, Median width at T60 =21 μm, Wilcoxon matched pairs test was used, two-tailed P =<0.0001, a significant difference in width. (H) Anterior displacement of the epithelium after onset of nurse cell dumping. We measured the location of the anterior epithelium in the X axis of an image just before onset of nurse dumping, and again about 60 min later, then normalized to 60min, for the anterior displacement shown here (See Supplementary Methods). n= 7, Median = 22 μm, IQR = 22.0 μm. Wilcoxon test was used to calculate a significant difference from zero using the method of Pratt, two tailed P =0.0156. For results presentation: avg. =23, s.d.=12.

Development • Supplementary information

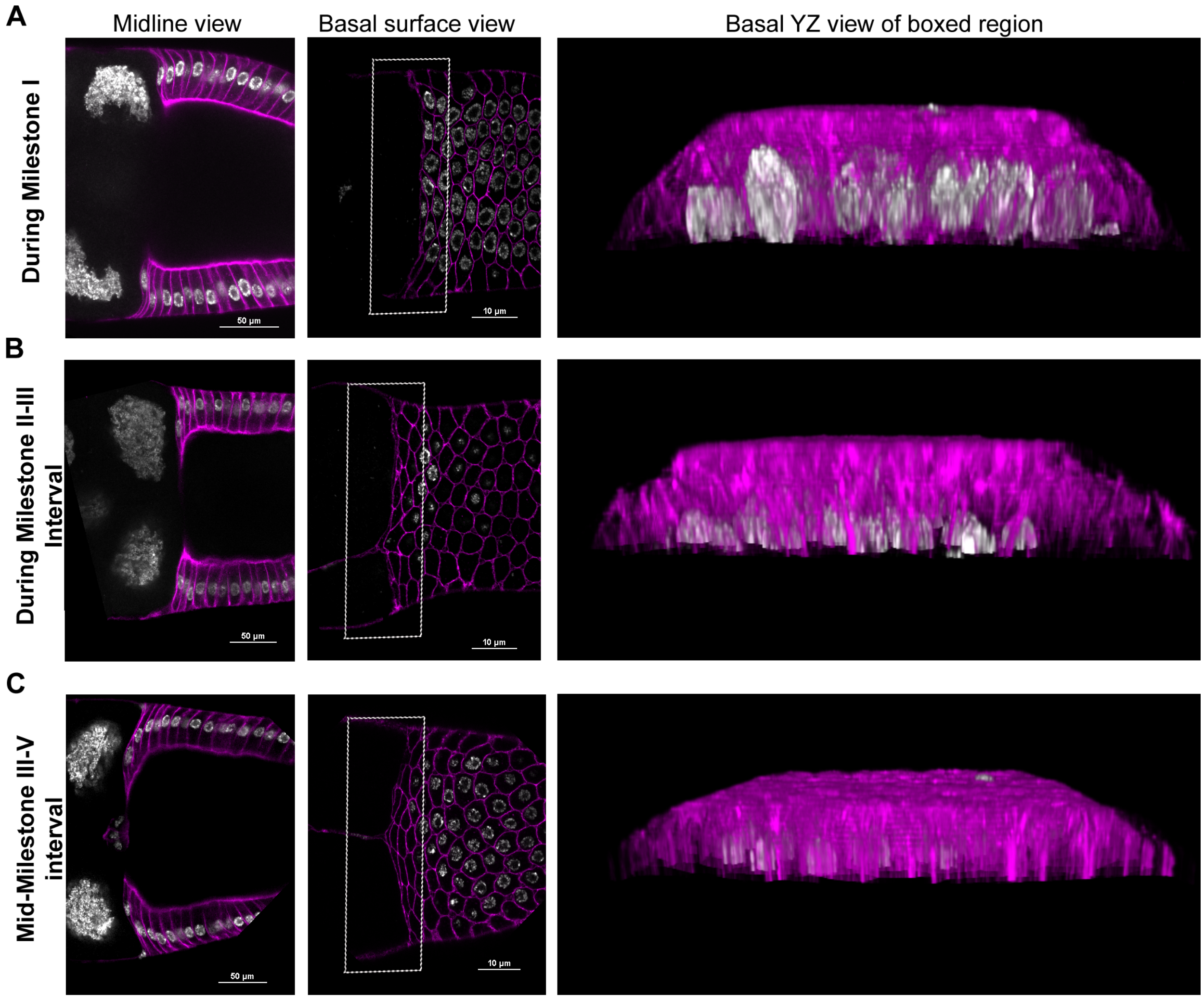

Fig. S2. Leading centripetal follicle cells shift their nuclei and cytoplasm away from the basement membrane as they move inward. (A-C) Apical lengthening of leading FCs is coincident with a shift of the nuclei and cytoplasm away from the basal surface. FC plasma membranes are marked by myristoylated-GFP (magenta, UAS-myr::GFP, driven by tj-Gal4, mef2-Gal80); nuclei labeled with DAPI (white). FC epithelial organization at the onset of centripetal migration (Top), as the first leading centripetal FC extends inward (Middle), and as the second leading centripetal FC begins to extend apically (Bottom). (A) Single, midline X-Y optical sections of egg chambers, at each. (B') Near basal, X-Y optical sections spanning the junction between the stretch FC (left) and

Development • Supplementary information

mainbody FC (right), with leading centripetal FC outlined in the dashed box. Leading centripetal FCs change shape with a greater narrowing in the X-axis than in the Y-axis; giving concomitant reduction in cross-sectional area, most evident in B”. (C) Cross-sectional, Y-Z views of the leading centripetal FC adjacent to the stretch FC, generated from volumetric projections of FC epithelium overlying the oocyte. The shift of nuclei away from the basal surface is most evident comparing the onset of leader FC apical extension to the onset of first leader FC inward movement, where nuclei are located closer to the bottom, with a greater distance between the nuclear edge to the basal surface at top. As second leading FCs start to elongate, first nuclei are no longer evident in Y-Z basal region projections. Fewer high intensity plasma membrane strands extend from top to bottom (white arrow),. (A-B) Anterior left. (C) Basal top. Scale bars in left panels 50 μm. Scale bars in middle panels 10 μm.

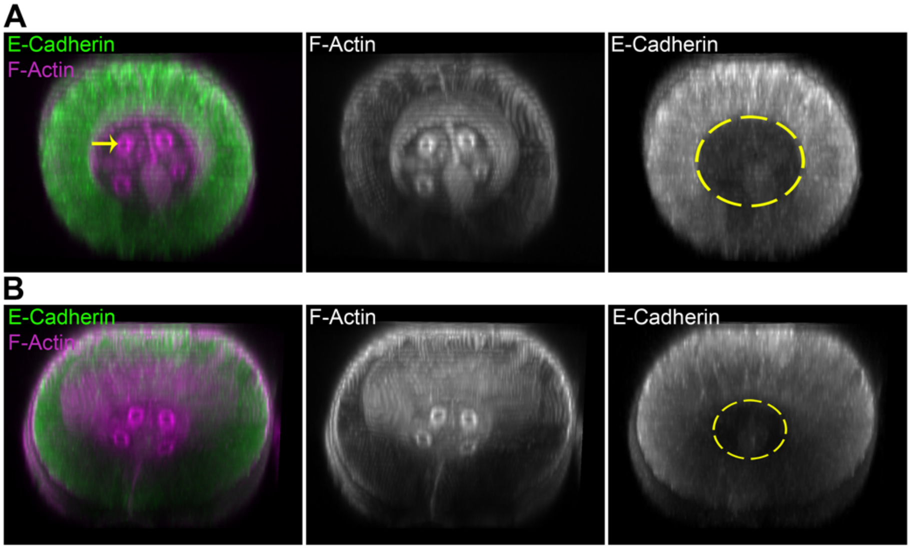

Fig. S3. Centripetal FCs have not migrated between ring canals by the end of stage 10B. Ring canals connect nurse cells to the oocyte, and are essential for transfer of nurse cell cytoplasm and organelles during stage 11, called nurse cell dumping. (A – B) Volumetric projections, generated from a fixed stage 10B egg chamber, imaged with low resolution to obtain full diameter images, then cross-sectioned digitally, looking down the A-P axis, towards the posterior. Each row shows merged fluorescence (left), F-actin (detected with phalloidin, Center), and anti-E-Cadherin (Right). (A) During early centripetal migration, ring canals are positioned centrally within the area where each posterior nurse cells contacts the oocyte (total contact area outlined by yellow dashed circle on right. Ring canals are lined with F-actin, one example indicated (yellow arrow). Border cell cluster was detected by low density phalloidin stain, just dorsal of center. (B) Shortly before the end of stage 10B, the leading edge of centripetal FCs was in close proximity to the ring canals, which were clustered together within the region of nurse cell-oocyte contact (total contact area outlined by yellow dashed circle).

Development • Supplementary information

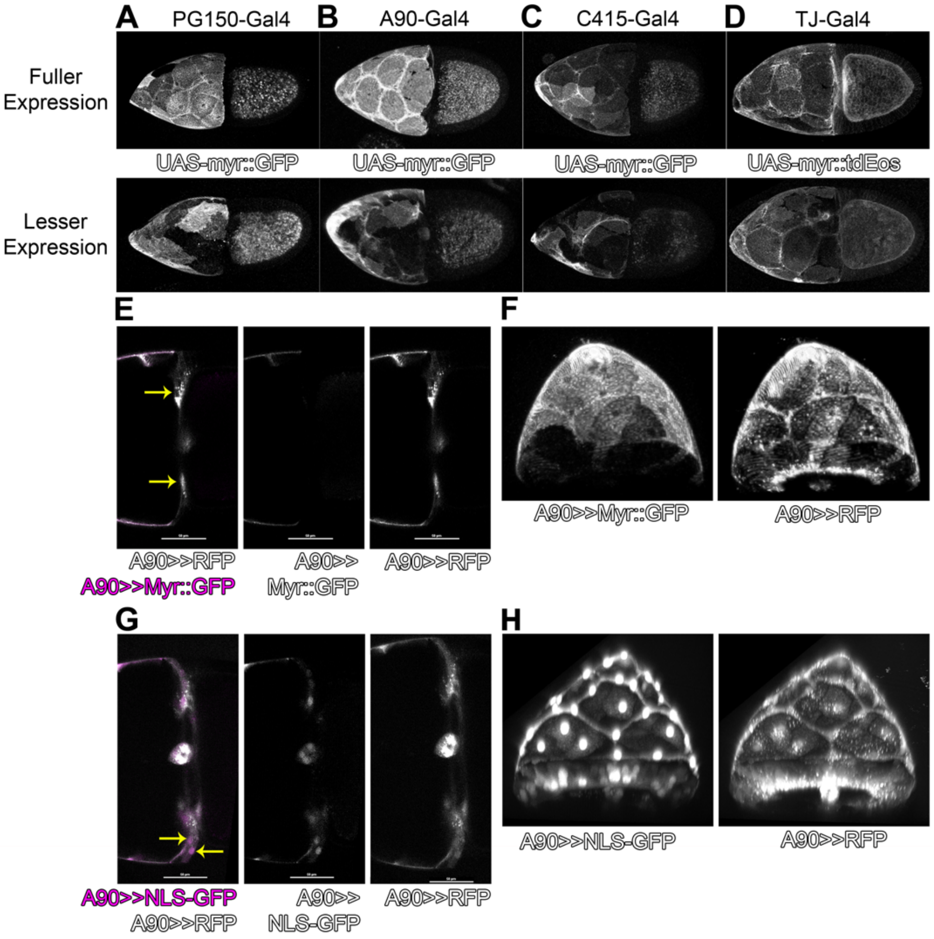

Fig. S4. Fluorescent labeling of stretch FCs is variable. (A – D) Volumetric projections of stage 10B egg chambers as viewed from the surface. Limitations of confocal imaging mean that resolution and intensity decrease with the depth along the z-axis. Thus, only z sections from the half egg chambers closest to the objective are used for volume projections. Stretch FCs are labeled with myristoylated GFP (A – C) or photoconverted myristoylated tdEOS (D) using the indicated Gal4 driver (PG150-Gal4, A90-Gal4, C415-Gal4, TJ-Gal4). Autofluorescent yolk granules within the oocyte are visible in the right portion of each image. Anterior is left. The top row shows examples where fluorescence was maximally expressed in stretch FCs. The bottom row shows examples where fluorescent labeling was weaker and variable from cell to cell. (E – H) Dual marker expression driven by Gal4-A90. Gal4-A90 chromosome carried UAS-mCD8:mChRFP (Jia et al., 2014), which was expressed together with different GFP-

Development • Supplementary information

tagged markers. Differences in the detection of fluorescence produced using A90-Gal4, were observed depending on which fluorescent UAS-responder was used. The left panel of each pair of images displays a composite of all fluorescence, while the panels to the right fluorescence from single markers alone. (E – F) A90-Gal4 drove expression of strong reporters in a few rows of columnar FCs. Cross-sectional view of an egg chamber expressing myristoylated GFP and cytoplasmic RFP. Myristoylated fluorescence appears only in the stretch FCs, whereas cytoplasmic RFP also was observed in the first few rows of centripetal cells (yellow arrows). (F) Data from E portrayed in a volumetric projection cross-sectioned digitally (for orientation see Fig. 1A) – note differences between detection of myristoylated GFP and cytoplasmic RFP within the ring of centripetally migrating FCs. (G – H) A similar effect is seen in a cross-sectional view of an egg chamber expressing nuclear-localized sfGFP (yellow arrows in G). (H) Data from G portrayed in a volumetric projection cross-sectioned digitally. Note the presence of GFP-positive nuclei in the ring of centripetally migrating cells. (E – G) Scale bars 50µm.

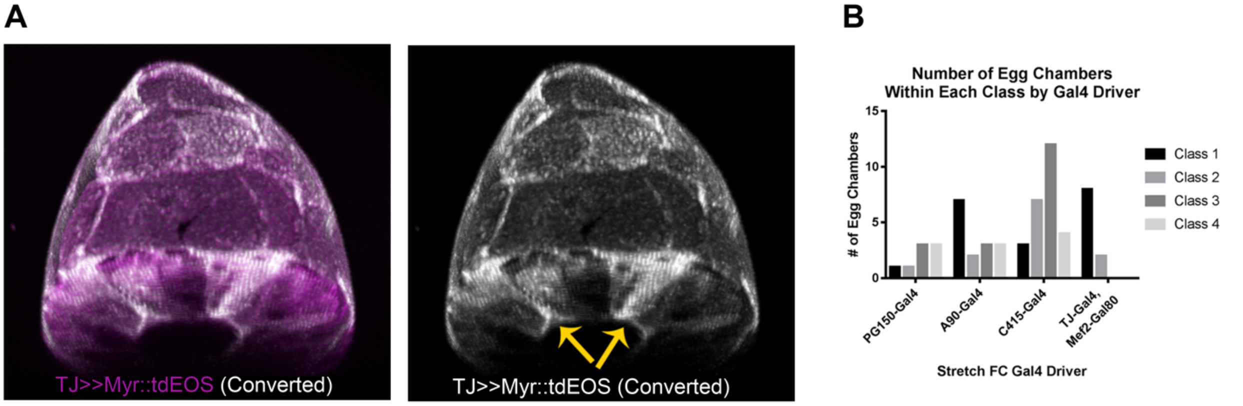

Fig. S5. Photoconversion of tdEOS detects only sporadic stretch FC extension. (A & C) Volumetric projections of stage 10B egg chambers cross-sectioned digitally, looking down the A-P axis, towards the anterior (for orientation see Fig. 1A). The left panel of each pair of images displays a composite of all fluorescence, while the panel to the right shows green or red fluorescence alone. (A) Photoconversion of stretch FCs expressing a membrane-localized tdEOS fluorophore also revealed a pattern of sporadic extension. Stretch FCs were photoconverted during late stage 10A and underwent time-lapse imaging for 5 hours before optical sections were acquired to determine if converted stretch cells extended inward (stretch FCs are indicated with yellow arrows in A, other weakly-labeled cells are centripetal cells that were inadvertently photoconverted due to their proximity to stretch FCs during conversion). (B) Graph showing how many egg chambers were in each class for each Gal4 driver.

Development • Supplementary information

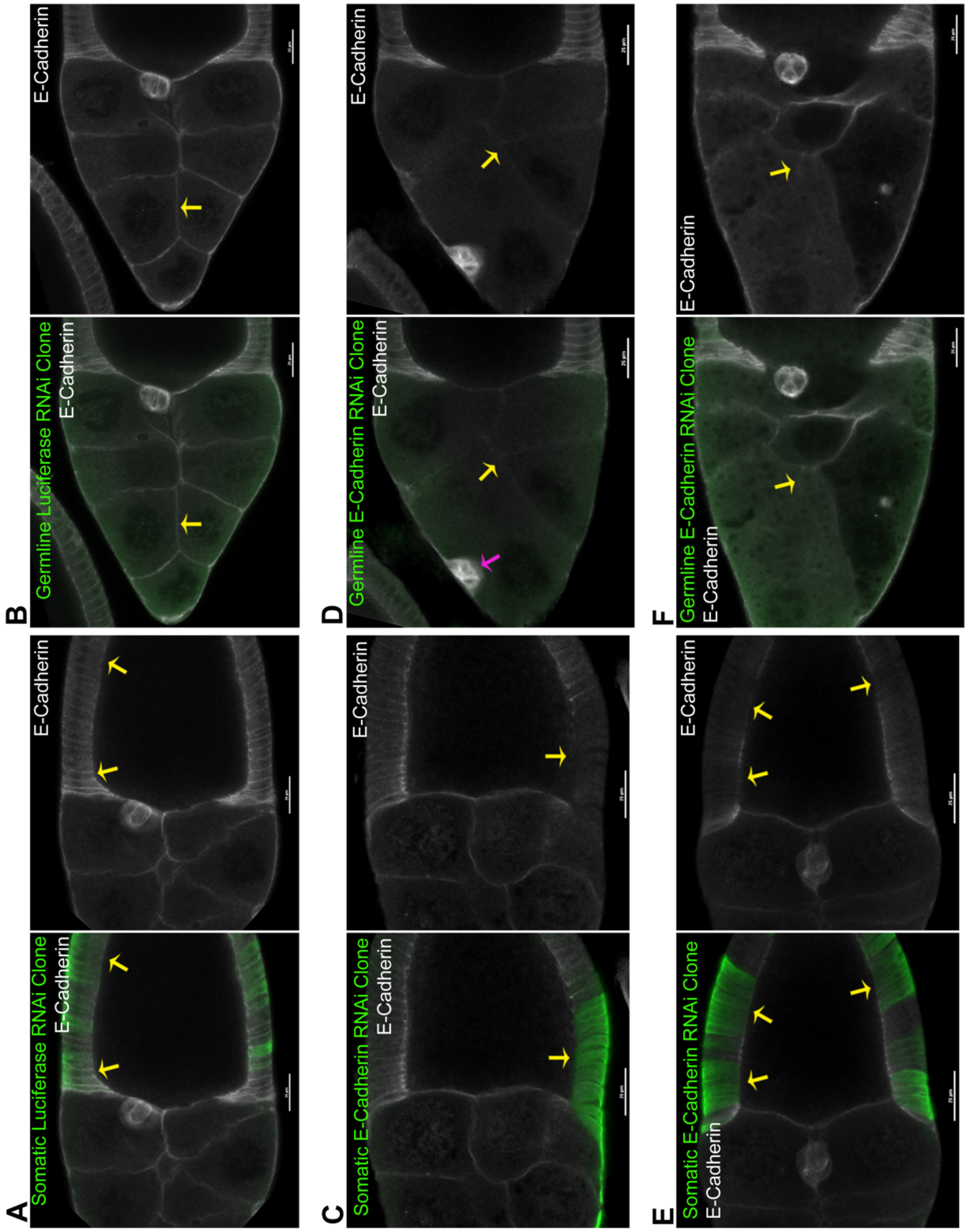

Fig. S6. Clonally expressed RNAi transgenes targeting *shotgun* reduced E-cadherin levels in FCs and nurse cells. (A – F) Cross-sectional views of egg chambers co-expressing GFP and UAS-regulated RNAi transgenes targeting *luciferase* or *shg* using the Flipout-Gal4 system. Egg chambers were fixed and

Development • Supplementary information

immunolabeled with anti-E-cadherin to detect E-cadherin protein levels in response to RNAi-mediated knockdown. Anterior is oriented to the left for all images, and dorsal is up for all panels except C and E. (A – B) Flipout-Gal4 clones co-expressing GFP and *luciferase* UAS-regulated RNAi transgenes (TRiP RNAi JF01355) exhibit wildtype levels of Shg in FCs (yellow arrows, A), and in nurse cells (yellow arrows). (C) Flip-out clones co-expressing GFP and *shg* UAS-regulated RNAi transgenes (TRiP RNAi HMS00693) exhibit reduced levels of Shg in FCs (yellow arrow) relative to controls (yellow arrows in A). (D) Flip-out clones co-expressing GFP and *shg* UAS-regulated RNAi transgenes (TRiP RNAi GL00646) exhibit reduced levels of Shg in the germ cells (yellow arrow in D) relative to controls (yellow arrow in B). Note the border cell cluster has deviated off course (magenta arrow in D), a known *shg* mutant phenotype (Niewiadomska et al., 1999). (E) Flip-out clones co-expressing GFP and *shg* UAS-regulated RNAi transgenes (TRiP RNAi JF02769) exhibit reduced levels of Shg in somatic FCs (yellow arrows in E) relative to controls (yellow arrows in A). (F) Flip-out clones co-expressing GFP and *shg* UAS-regulated RNAi transgenes (TRiP RNAi HMS00693) exhibit reduced levels of Shg in the nurse cells (yellow arrows in F) relative to controls (yellow arrow in B). Scale bars 25μm.

Development • Supplementary information

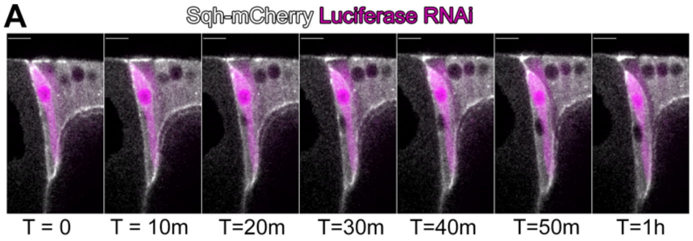

Fig. S7. Example of a GFP+, control, leader centripetal FC shape during ingression. Enlarged images related to Fig. 5, fortuitously showing a single leader FC labeled with Sqh-mCherry (white), and co-expressing a control UAS-regulated dsRNA transgene with GFP (magenta) using the Flipout-Gal4 system. Anterior is left, basal is up. Germ cells lack detectable Sqh-mCherry expression; oocyte corner is visible in lower left; nurse cell is visible on right. The thin, anterior GFP- centripetal FC neighbor may be either an extending stretch cell, or the first leading FC. The elongated leading edge of the strongly GFP+ cell overlies the leading edge of its anterior neighbor. The posterior leading FC neighbor is weakly GFP+, possibly from a neighboring FC in the z-axis. The GFP+ FC has a thin training edge, as it separates from the basal surface. Intense Sqh-mCherry accumulation is visible at the leading and trailing edges of the GFP+ leading FC, as well as at the basal surfaces of more posterior centripetal FCs. This Sqh-mCherry accumulation points toward possible dynamic actinomyosin localization, but greater temporal and spatial resolution are needed to investigate further.

Development • Supplementary information

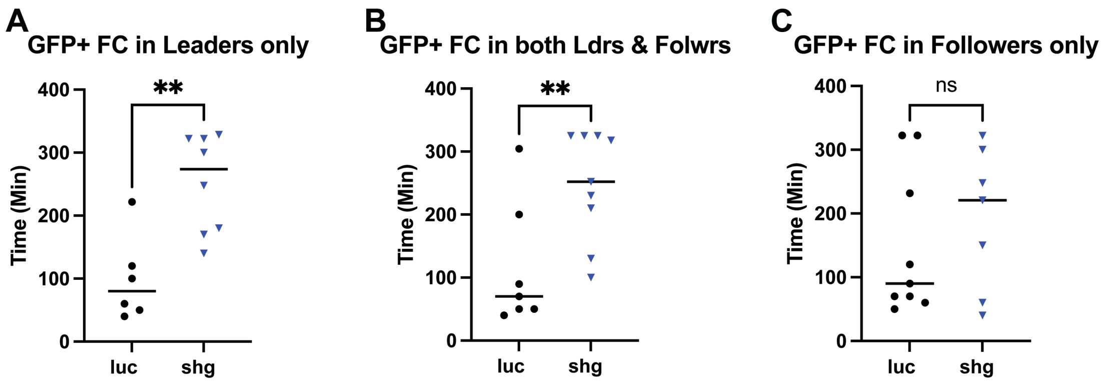

Fig. S8. Samples with *shg*-depleted leading FCs were significantly delayed in basal thinning. We determined the time interval between Milestone II and Milestone III, or for samples that did not undergo Milestone III, a proxy time for Milestone III that was 30 min. later than the end of the timelapse sequence (see Methods). **(A) Samples in the GFP$^+$ Leaders only class**. Luciferase dsRNA control Leader1 FCs (black dots) had a median time of 80.0 min, n=6. *shg* dsRNA samples (blue triangles) had a median time of 273.9 min., n=8. Two-tailed exact P=0.0047, by Mann-Whitney analysis. **(B) Samples in the Both GFP$^+$ Leaders and Followers class**. Luciferase dsRNA control Leader1 FCs (black dots) had a median time of 70.0 min, n=7. *shg* dsRNA samples (blue triangles) had a median time of 252.1 min., n=9. Two-tailed exact P=0.0074, by Mann-Whitney analysis. **(C) Samples in the GFP$^+$ Followers only class**. Luciferase dsRNA control samples (black dots) had a median time of 90.0 min, n=9. *shg* dsRNA samples (blue triangles) had a median time of 220.8 min., n=7. The median time interval for *shg*-depleted Followers only samples was longer than the median for the control, but the variance of control samples was large, so the difference was not significant. Two-tailed exact P=0.7767, by Mann-Whitney analysis. luc = Luciferase dsRNA control samples. shg = dsRNA samples.

Development • Supplementary information

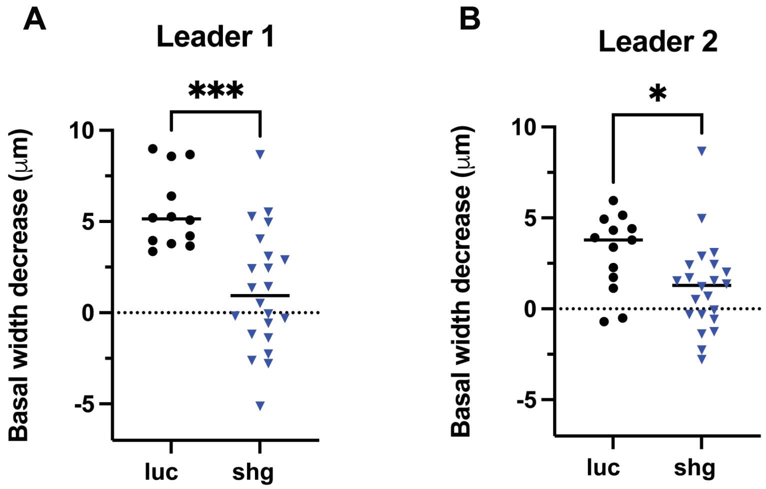

Fig. S9. Decrease in Leader FC basal width from Milestone II to Milestone III, across all samples with GFP$^+$ leader FCs. Basal width decrease was calculated for samples from the GFP+ Leaders class and the Both GFP$^+$ Leaders and Followers class. Basal width at Milestone II was subtracted from that at Milestone III, or end of timelapse sequence for samples that did not undergo Milestone III. No adjustments were made for the latter samples. **(A) Basal width decrease for first leader FC**. Luciferase dsRNA control Leader1 FCs (black dots) decreased by a median of 5.15 μm, n=12. *shg* dsRNA Leader1 FCs (blue triangles) decreased by a median of 0.94 μm, n=22. Two-tailed exact P=0.0002, by Mann-Whitney analysis. **(B) Basal width decrease for second leader FC**. Luciferase dsRNA control Leader2 FCs (black dots) decreased by a median of 3.79 μm, n=13. *shg* dsRNA Leader2 FCs (blue triangles) decreased by a median of 1.29 μm, n=22. Two-tailed exact P=0.0175, by Mann-Whitney analysis. luc = Luciferase dsRNA control samples. shg = dsRNA samples.

Development • Supplementary information

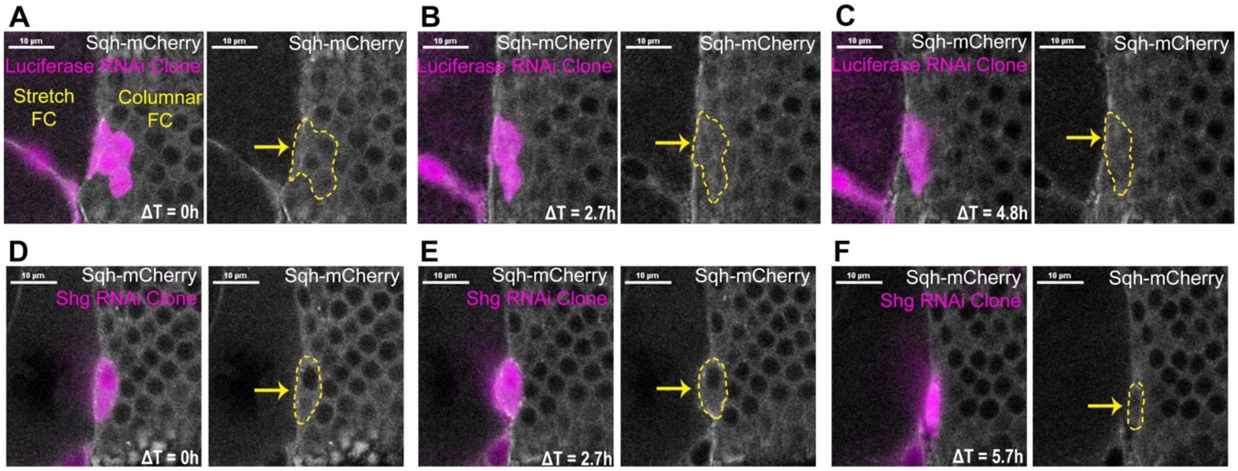

Fig. S10. E-Cadherin-depleted leading centripetal FCs undergo delayed reduction of basal cross-sectional area. (A – F) Frames from time-lapse sequences of stage 10B egg chambers undergoing centripetal migration, showing the basal-most optical section (for orientation see green region in Fig. 1A). Egg chambers are labeled with Sqh-mCherry (white) and clonally express *luciferase* or *shg* UAS-regulated RNAi transgenes (magenta) with the use of the Flipout-Gal4 system. Left panel of each pair of images shows merges fluorescence. Right panel shows Sqh-mCherry fluorescence alone. In each image, stretch FCs are located to the left, and columnar FCs to the right. (A – C) When FCs expressing UAS-regulated RNAi transgenes targeting *lucerifase* are positioned near the stretch FC/columnar FC interface (yellow arrow in A), over about 4.8 hours, the anterior-most centripetal FCs ingress inward and reduce their basal cross-sectional area near the basement membrane (outlined by yellow dotted line in all panels) at a rate similar to neighboring cells. (D – F) When cells depleted for *shg* are positioned near the stretch FC/columnar FC interface (yellow arrow in D'), over about 5.7 hours the anterior-most cells elongated inward (Fig. 5A, C), but were delayed in reducing their basal cross-sectional area near the basement membrane (outlined by yellow dotted line in all panels) relative to neighboring cells and controls. Scale bars 10µm.

Development • Supplementary information

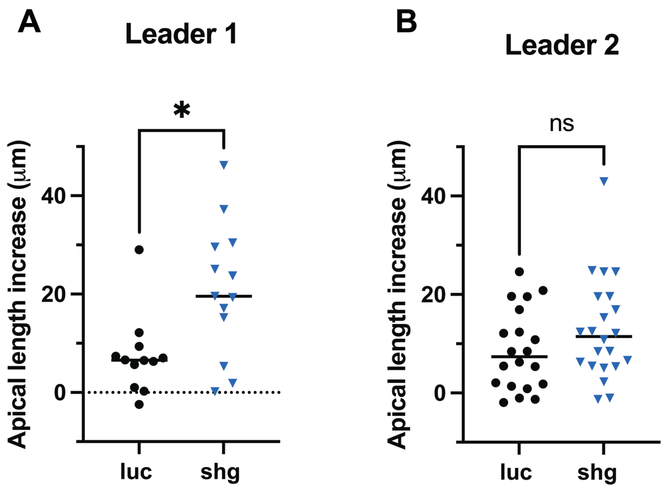

Fig. S11. Increase in Leader FC Apical-Basal length across all samples with GFP $^{+}$ leader FCs. Length measured at Milestone II was subtracted from that at Milestone III, or end of timelapse sequence for samples that did not undergo Milestone III. No adjustments were made for the latter samples. **(A) Apical-Basal length increase for first leader FC**. Luciferase dsRNA control Leader1 FCs (black dots) increased by a median of 6.58 μm, n=12. *shg* dsRNA Leader1 FCs (blue triangles) increased by a median of 19.58 μm, n=13. Two-tailed exact P=0.0188, by Mann-Whitney analysis. **(B) Apical-Basal length increase for second leader FC**. Luciferase dsRNA control Leader2 FCs (black dots) increased by a median of 7.36 μm, n=20. *shg* dsRNA Leader2 FCs (blue triangles) decreased by a median of 11.47 μm, n=22. Two-tailed exact P=0.1775, by Mann-Whitney analysis. luc = Luciferase dsRNA control samples. shg = dsRNA samples.

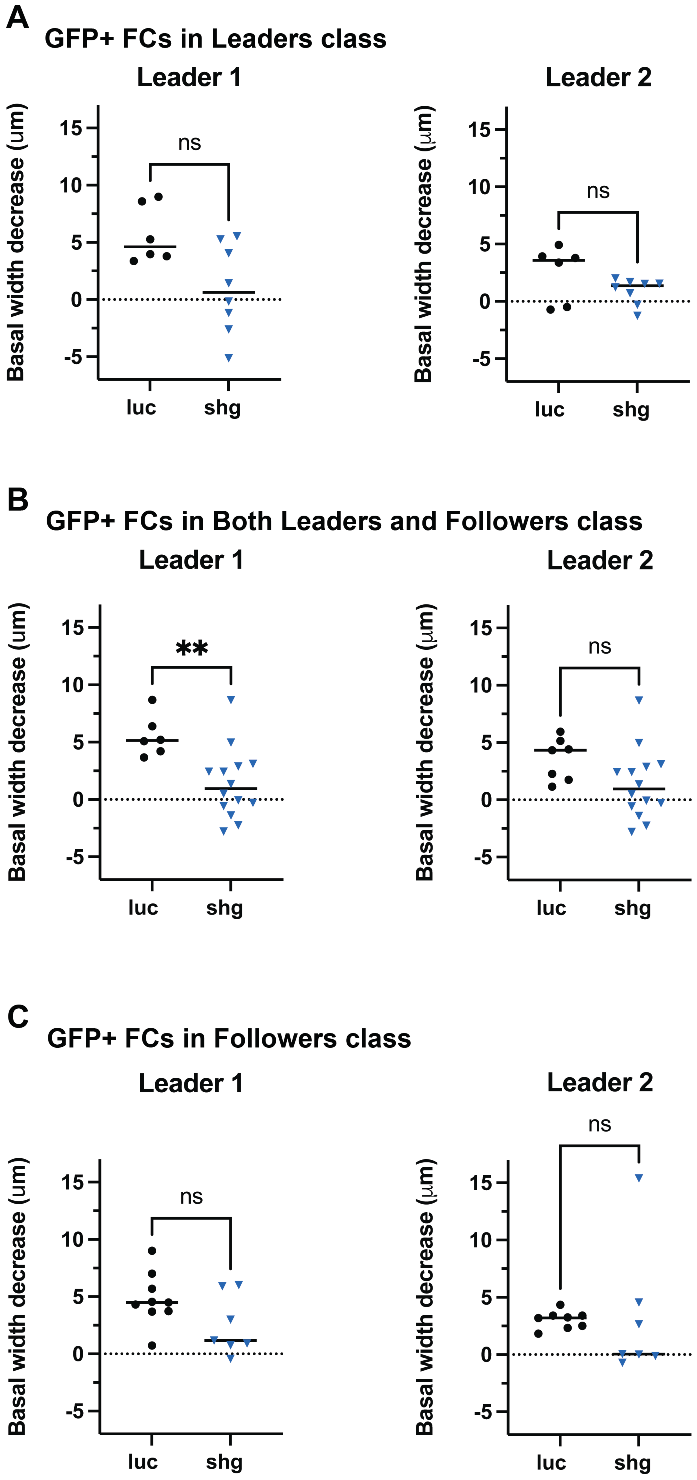

Fig. S12. Decrease in Leader FC basal width from Milestone II to Milestone III, for each sample class. Basal width decrease was calculated for the first leader FC

Development • Supplementary information

(left) and the second leader FC (right) for samples from each class. Basal width at Milestone II was subtracted from that at Milestone III, or end of timelapse sequence for samples that did not undergo Milestone III. No adjustments were made for the latter samples. **(A) Samples in the GFP$^+$ Leaders only class**. First Leader FC (left): Luciferase dsRNA control Leader1 FCs (black dots) decreased by a median of 4.61 μm, n=6. *shg* dsRNA Leader1 FCs (blue triangles) decreased by a median of 0.625 μm, n=8. Two-tailed exact P=0.1079, by Mann-Whitney analysis. Second Leader FC (right): Luciferase dsRNA control Leader2 FCs (black dots) decreased by a median of 3.59 μm, n=6. *shg* dsRNA Leader2 FCs (blue triangles) decreased by a median of 1.375 μm, n=8. Two-tailed exact P=0.2284, by Mann-Whitney analysis. **(B) Samples in the Both GFP$^+$ Leaders and Followers class**. First Leader FC (left): Luciferase dsRNA control Leader1 FCs (black dots) decreased by a median of 5.15 μm, n=6. *shg* dsRNA Leader1 FCs (blue triangles) decreased by a median of 0.940 μm, n=14. Two-tailed exact P=0.0023, by Mann-Whitney analysis. Second Leader FC (right): Luciferase dsRNA control Leader2 FCs (black dots) decreased by a median of 4.32 μm, n=7. *shg* dsRNA Leader2 FCs (blue triangles) decreased by a median of 0.940 μm, n=14. Two-tailed exact P=0.0793, by Mann-Whitney analysis. **(C) Samples in the GFP$^+$ Followers only class**. First Leader FC (left): Luciferase dsRNA control Leader1 FCs (black dots) decreased by a median of 4.49 μm, n=9. *shg* dsRNA Leader1 FCs (blue triangles) decreased by a median of 1.17 μm, n=7. Two-tailed exact P=0.1738, by Mann-Whitney analysis. Second Leader FC (right): Luciferase dsRNA control Leader2 FCs (black dots) decreased by a median of 3.22 μm, n=8. *shg* dsRNA Leader2 FCs (blue triangles) decreased by a median of 0.05 μm, n=7. Two-tailed exact P=0.3357, by Mann-Whitney analysis. luc = Luciferase dsRNA control samples. shg = dsRNA samples.

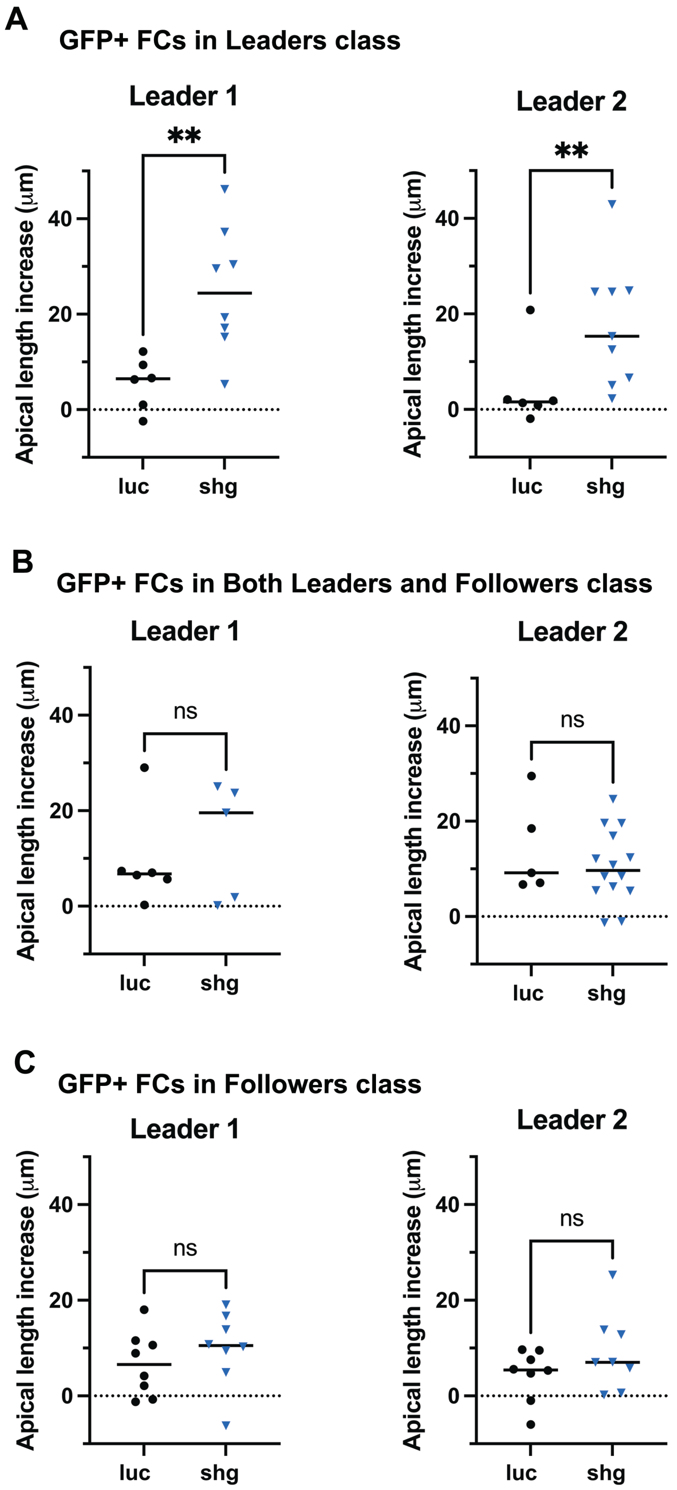

Development • Supplementary information

Fig. S13. Increase in Leader FC apical-basal length from Milestone II to Milestone III for each sample class. Length measured at Milestone II was subtracted from that at Milestone III, or end of timelapse sequence for samples that did not undergo Milestone III. No adjustments were made for the latter samples. **(A) Samples in the GFP$^+$ Leaders only class**. Apical-Basal length increase for first leader FC (left) and second leader FC (right). First Leader FC (left): Luciferase dsRNA control Leader1 FCs (black dots) increased by a median of 6.49 µm, n=6. *shg* dsRNA Leader1 FCs (blue triangles) increased by a median of 24.45 µm, n=8. Two-tailed exact P=0.0080, by Mann-Whitney analysis. Second Leader FC (right): Luciferase dsRNA control Leader2 FCs (black dots) increased by a median of 1.59 µm, n=6. *shg* dsRNA Leader1 FCs (blue triangles) increased by a median of 15.3 µm, n=9. Two-tailed exact P=0.0076, by Mann-Whitney analysis. **(B) Samples in the Both GFP$^+$ Leaders and Followers class**. Apical-Basal length increase for first leader FC (left) and second leader FC (right). First Leader FC: Luciferase dsRNA control Leader1 FCs (black dots) increased by a median of 6.8 µm, n=6. *shg* dsRNA Leader1 FCs (blue triangles) increased by a median of 19.6 µm, n=5. Two-tailed exact P=0.9307, by Mann-Whitney analysis. Second Leader FC (right): Luciferase dsRNA control Leader2 FCs (black dots) increased by a median of 9.2 µm, n=5. *shg* dsRNA Leader1 FCs (blue triangles) increased by a median of 9.7 µm, n=14. Two-tailed exact P=0.5593, by Mann-Whitney analysis. **(C) Samples in the GFP$^+$ Followers only class**. First Leader FC (left): Luciferase dsRNA control Leader1 FCs (black dots) increased by a median of 6.6 µm, n=8. *shg* dsRNA Leader1 FCs (blue triangles) increased by a median of 10.5 µm, n=8. Two-tailed exact P=0.3282, by Mann-Whitney analysis. Second Leader FC (right): Luciferase dsRNA control Leader2 FCs (black dots) increased by a median of 5.4 µm, n=8. *shg* dsRNA Leader2 FCs (blue triangles) increased by a median of 7.0 µm, n=8. Two-tailed exact P=0.2786, by Mann-Whitney analysis. luc = Luciferase dsRNA control samples. shg = dsRNA samples.

Development • Supplementary information

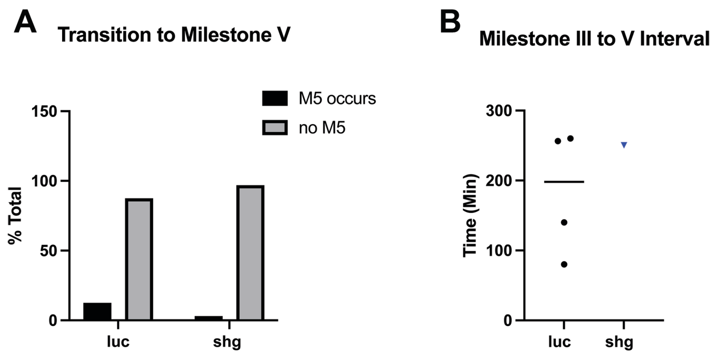

Fig. S14. Transition to Milestone 5 was recovered rarely in both control and *shg* RNAi samples. (A) Percent of samples with scored anterior clones that showed Milestone V. 14% of luciferase dsRNA mosaic egg chambers exhibited Milestone V, with at least one instance across each of the three classes, n=28. In contrast, 3% of *shg* dsRNA mosaic egg chambers exhibited Milestone V, n=32. This sample had GFP+ E-Cadherin-depleted FCs in both leader and follower FCs. Sample size was too low for descriptive statistics. **(B)** Time for transition from Milestone III to Milestone V. Sample size was too low for descriptive statistics. In our initial analysis, the median time between Milestone III and Milestone V was 90 min. (Fig. 3). luc = Luciferase dsRNA control samples. shg = *shg* dsRNA samples.

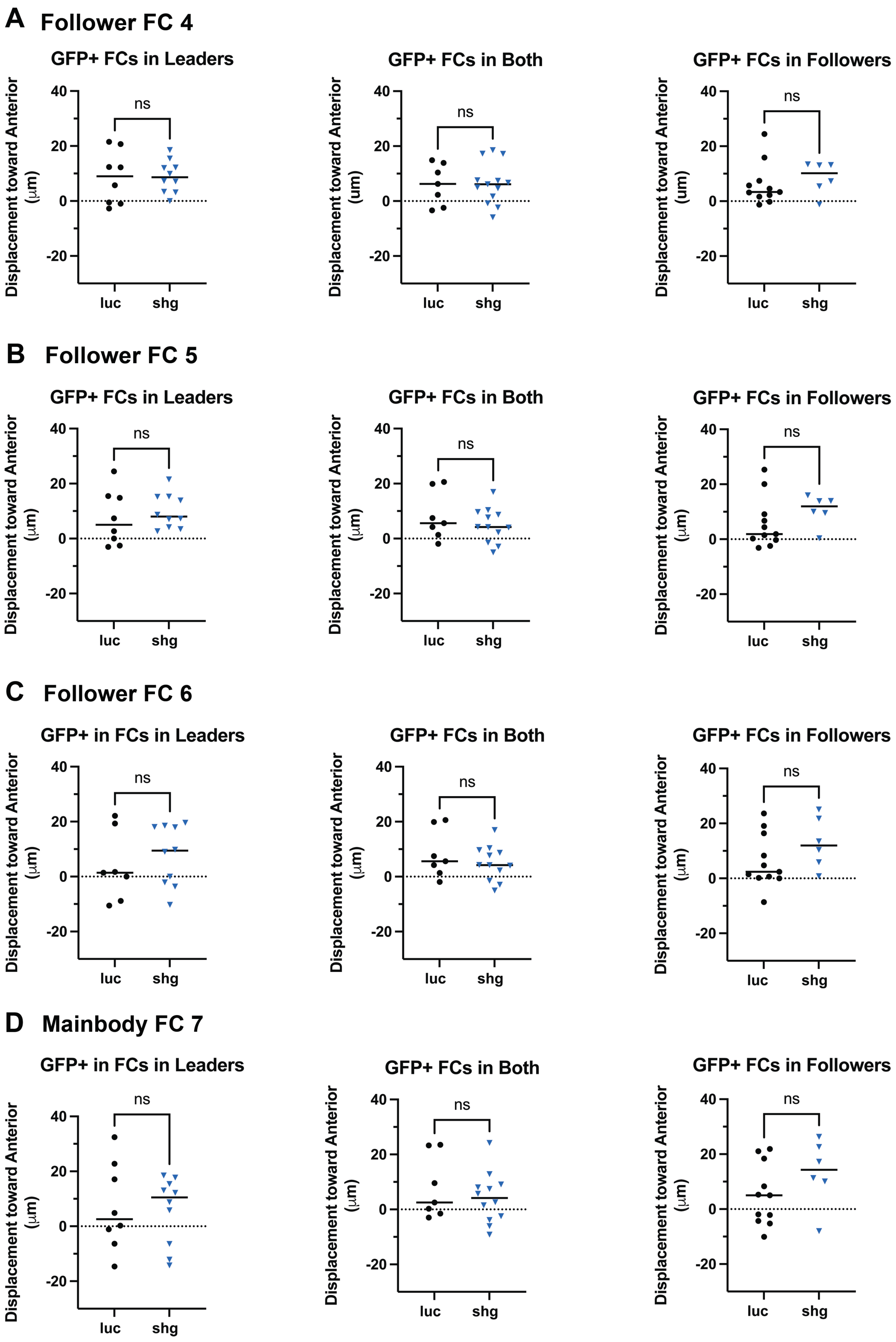

Development • Supplementary information

Fig. S15. Follower FC displacement toward anterior over the length of the timelapse sequence. To assess the movement of following FCs to the anterior during the approximate period of Milestone II to Milestone III or later, we determined the displacement of follower FCs from the beginning to the end of the timelapse sequence. As a proxy for each FC, we identified its nucleus as a region with low to absent MCherry fluorescence from Sqh::MCherry. Nuclei were numbered one through eight, from anterior to posterior. At the beginning and end of the timelapse, we measured the distance from the nurse cell:FC interface to a specific FC nucleus. Length measured at the beginning was subtracted from that at the end of timelapse sequence. We expected that follower FCs progressing to the Milestone V transition would show a positive displacement toward the nurse cell:FC interface and that stalled follower FCs would show a negative displacement, due to their slight flattening as the oocyte expands. See Methods for independent variables contributing to the variability in the data. For all panels: Luciferase dsRNA control FCs (luc) = black dots. *shg*-depleted FCs (shg) = blue inverted triangles. In all cases, significance was tested with the Mann-Whitney U test, and yielded an exact, two-tailed P value. **(A) Displacement for Follower FC4**. Nucleus 4 showed no significant difference between control GFP+ FCs and *shg*-depleted GFP+ FCs, across all sample classes. (left) GFP+ Leaders only class: luc Follower FC4 was displaced by a median of 9.0 μm, n=8. shg Follower FC4 was displaced by a median of 8.6 μm, n=10, P=0.9654. (middle) GFP+ Leaders and Followers class (Both) class: luc Follower FC4 was displaced by a median of 6.2 μm, n=7. shg Follower FC4 was displaced by a median of 5.0 μm, n=5, P=0.8763. (right) GFP+ Followers only class: luc Follower FC4 was displaced by a median of 3.4 μm, n=11. shg Follower FC4 was displaced by a median of 3.1 μm, n=4, P=0.6608. **(B) Displacement for Follower FC5**. Nucleus 5 showed no significant difference between *shg*-depleted GFP+ FCs and control GFP+ FCs, across all sample classes. (left) GFP+ Leaders only class: luc Follower FC5 was displaced by a median of 5.0 μm, n=8. shg Follower FC5 was displaced by a median of 8.0 μm, n=10, P=0.4598. (middle) GFP+ Leaders and Followers class (Both) class : luc Follower FC5 was displaced by a median of 5.6 μm,

Development • Supplementary information

n=7. shg Follower FC5 was displaced by a median of 4.3 μm, n=5, P=0.7914. (right) GFP+ Followers only class: luc Follower FC5 was displaced by a median of 1.9 μm, N11. shg Follower FC5 was displaced by a median of13.9 μm, n=4, P=0.1804. **(C) Displacement for Follower FC6**. Nucleus 6 showed no significant difference between *shg*-depleted GFP+ FCs and control GFP+ FCs, across all sample classes. (left) GFP+ Leaders only class: luc Follower FC6 was displaced by a median of 1.4 μm, n=7. shg Follower FC6 was displaced by a median of 10.39 μm, n=5P=0.2020. (middle) GFP+ Leaders and Followers class (Both) class: luc Follower FC6 was displaced by a median of 5.0 μm, n=7. shg Follower FC6 was displaced by a median of 5.7 μm, n=11, P=0.8601. (right) GFP+ Followers only class: luc Follower FC6 was displaced by a median of 2.4 μm, N11. shg Follower FC6 was displaced by a median of 10.4 μm, n=5, P=0.1804. **(D) Displacement for Mainbody FC7**. Nucleus 7 showed no significant difference between *shg*-depleted GFP+ FCs and control GFP+ FCs, across all sample classes. (left) GFP+ Leaders only class: luc Mainbody FC7 was displaced by a median of 5.0 μm, n=11. shg Mainbody FC7 was displaced by a median of 11.33 μm, n=5, P=0.2212. (middle) GFP+ Leaders and Followers class (Both) class: luc Mainbody FC7 was displaced by a median of 2.5 μm, n=7. shg Mainbody FC7 was displaced by a median of 5.7 μm, n=11, P=0.8601. (right) GFP+ Followers only class: luc Mainbody FC7 was displaced by a median of 5.0 μm, N11. shg Mainbody FC7 was displaced by a median of 11.3 μm, n=5, P=0.2212.

Development • Supplementary information

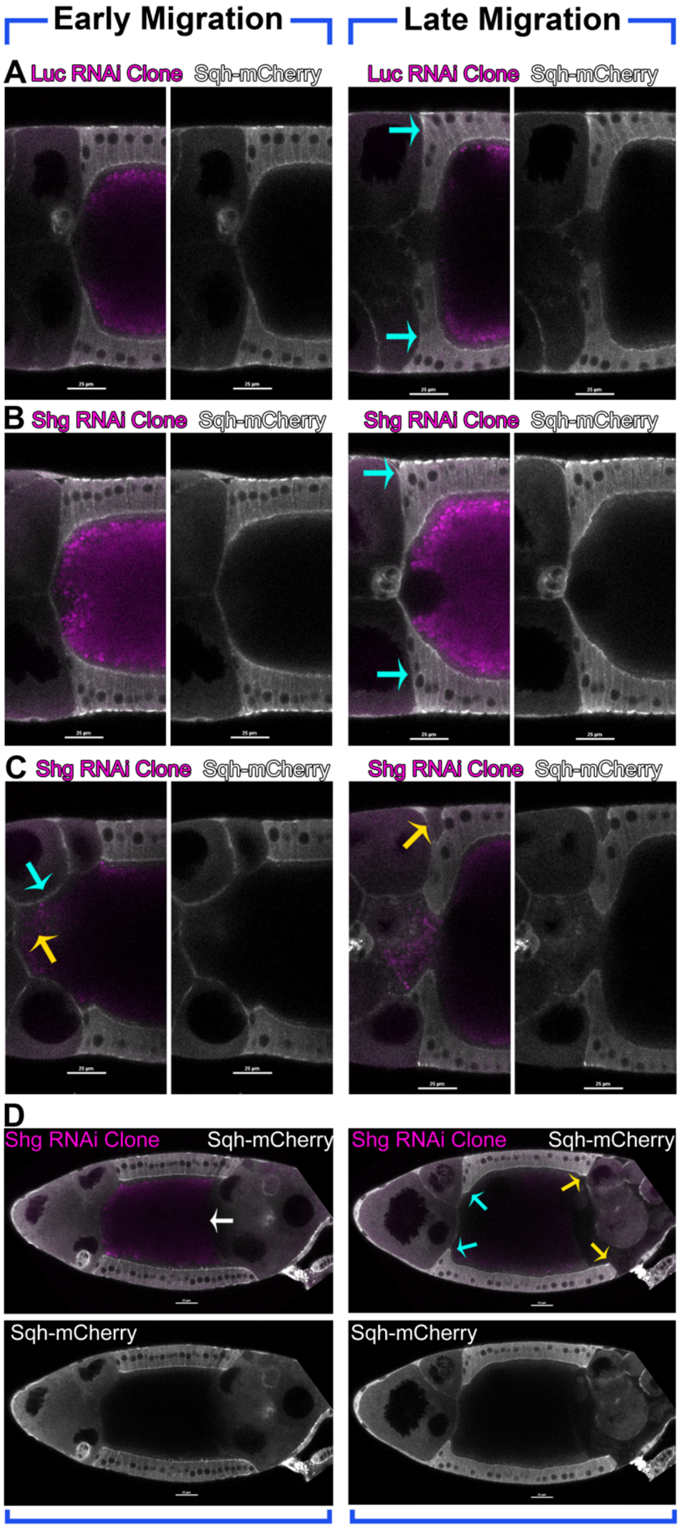

Development • Supplementary information

Fig. S16. Knockdown of *shotgun* in germ cells resulted in abnormal but completed centripetal migration. (A – D) The Flipout-Gal4 technique was used to deplete *shg* (B-D) or *luciferase* (A) by expressing UAS-regulated RNAi transgene transgenes in heatshock-induced germ cell clones. Sqh-mCherry was used to label all cells to observe migration during time-lapse imaging. Weak GFP expression (magenta) marks the germ cells expressing RNAi transgene, while strong magenta puncta within the oocyte originated from yolk granule autofluorescence. Each row of panels is a set of frames from a time-lapse series of one egg chamber, with the left pair showing an earlier point in time than the right pair. Anterior is oriented to the left, and dorsal is up. The left panel of each pair displays merged fluorescence, while the right panel Sqh-mCherry fluorescence alone. (A) Control germ cell clones expressing UAS-regulated RNAi transgene transgenes targeting *luciferase* exhibited normal timing and morphologies of Milestones during migration. (B) In experiments with weaker germ cell *shotgun* knockdown, leading FCs appeared more elongated than normal (blue arrows). (C) Egg chambers with reduced Shg levels in germ cells exhibited nurse cells that were abnormally round (blue arrow in A) as previously reported by (Oda et al., 1997). The oocyte bulged into nurse cell space (yellow arrow). In these egg chambers, the leading FCs that had ingressed appeared to be thicker in their lateral direction and appeared to adhere more to one another than to the underlying germ cells. Wildtype centripetal FCs migrated inward to form an anterior epithelium with only minor defects, including gaps between the nurse cells and FCs (yellow arrow). (D) Occasionally strong *shotgun* knockdown in the germ cells resulted in a mispositioned oocyte, a known *shotgun* mutant phenotype (Godt and Tepass, 1998; González-Reyes and St Johnston, 1998; Niewiadomska et al., 1999). Leading FCs near the nurse cell/oocyte interface initiated Milestones I and II (blue arrows) but had not progressed to Milestone III or later. Potential leading FCs that develop from failed induction of posterior FC fates (yellow arrows), were located over the posterior oocyte (white arrow), with no adjacent nurse cells. These posterior leading FCs showed little elongation (delayed or stalled Milestone I), and lacked progression to Milestone II that is seen for anterior leading FCs (blue arrows). The top panels of left and right pairs displays merged fluorescence, while the bottom panels show Sqh-mCherry alone. Scale bars 25μm.

Development • Supplementary information

Table S1. Reagents

Click here to download Table S1

Table S2. Full Genotypes Per Figure Panel / Movie

For a full list of original *Drosophila* stocks used in this study and their sources, see supplemental reagent table.

Click here to download Table S2

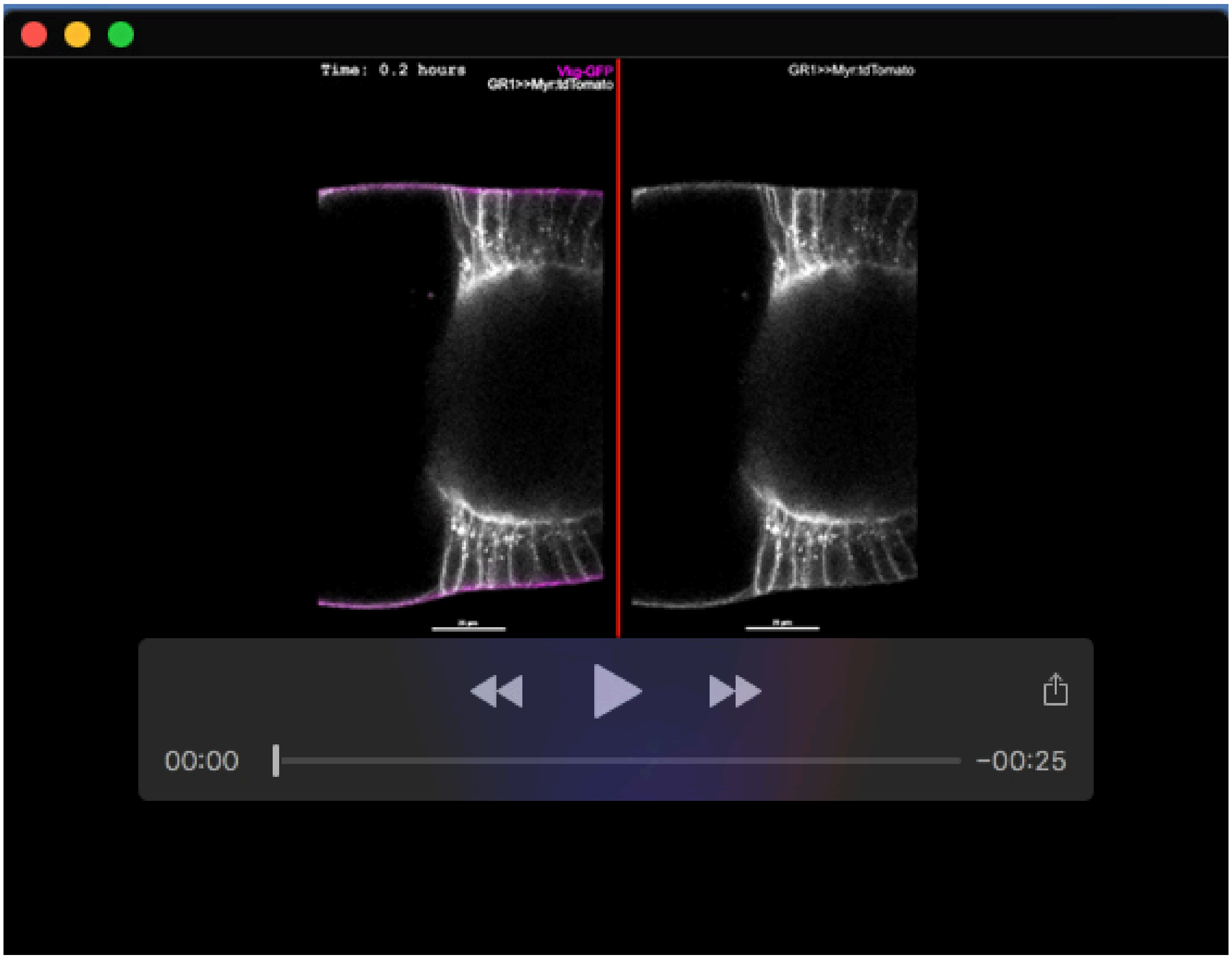

Movie 1, related to Figure 1: Leading centripetally migrating cells appear to reduce contact with the basement membrane. A series of two time-lapse Movies consisting of the data from Fig. 1F,G showing that leading centripetally migrating cells appeared to reduce contact with the basement membrane as migration proceeded. Part 1 shows a stage 10B egg chamber over the course of 2.7 hours with a membrane-localized fluorophore (Myr::tdTomato) and Vkg-GFP. Part 2 shows a stage 10B egg chamber over the course of 3.1 hours with a photoconvertible membrane-localized fluorophore (Myr::tdEOS) that has been photoconverted in the leading centripetal cells. Yellow arrows indicate leading FCs as they reduce contact with the basement membrane. Full genotypes for this Movie are available in Supplemental Table 2. Scale bars 25µm. Anterior is oriented to the left, and dorsal is up.

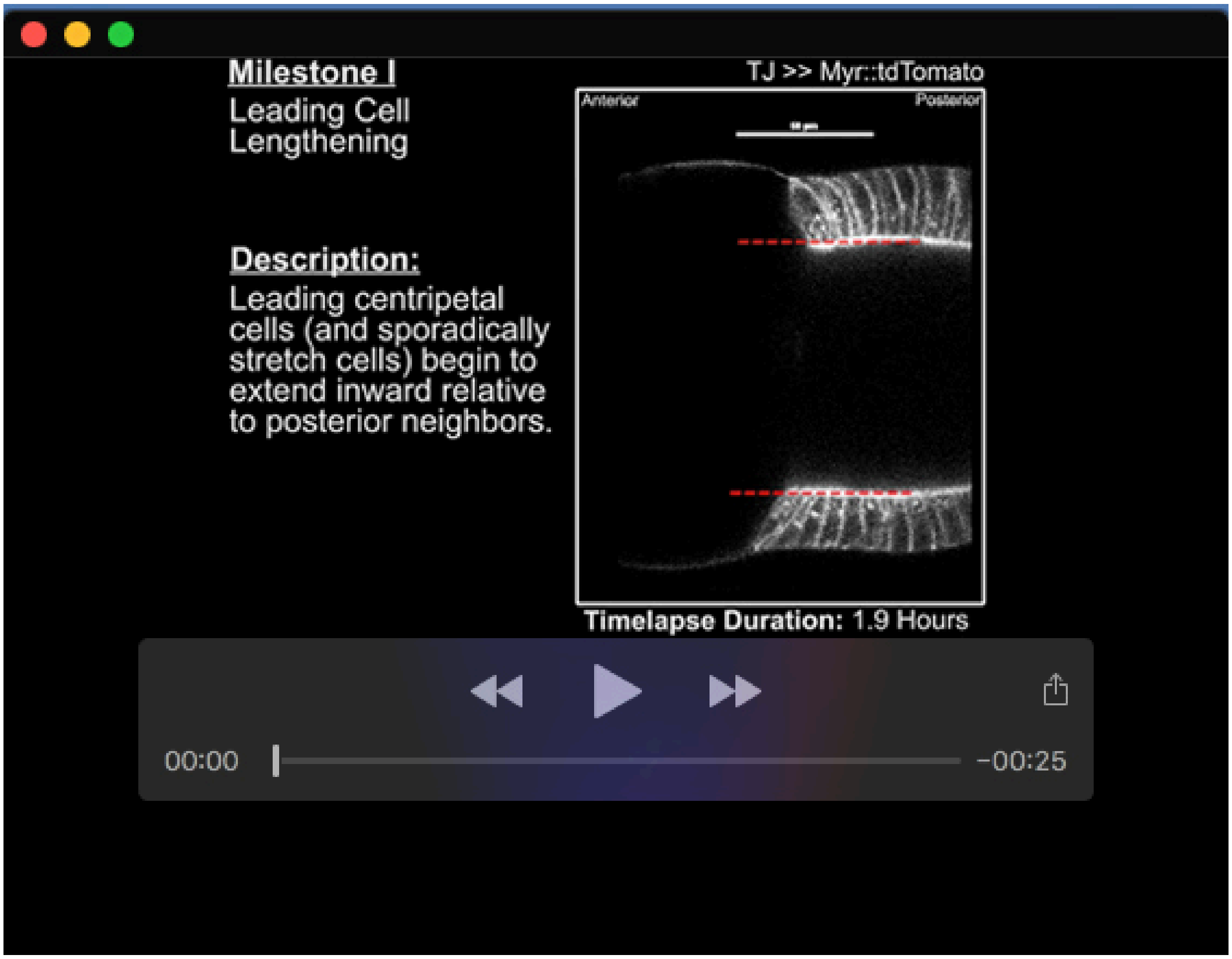

Movie 2, related to Figure 2: Annotated milestones observed during centripetal migration. A series of eight time-lapse Movies that include the data from Fig. 2A-G showing different egg chambers undergoing each of the milestones identified. Cross-sectional views are shown, labeled with a membrane localized fluorophore (TJ-Gal4 >> UAS-Myr::tdTomato). Anterior is oriented to the left, and dorsal is up. Each milestone is briefly listed along with a short description. Colored arrows indicate features that define each milestone. A five-hour time-lapse of a single stage 10B egg chamber progressing through all milestones is displayed at the end of the Movie. Full genotypes for this Movie are available in Table S2.

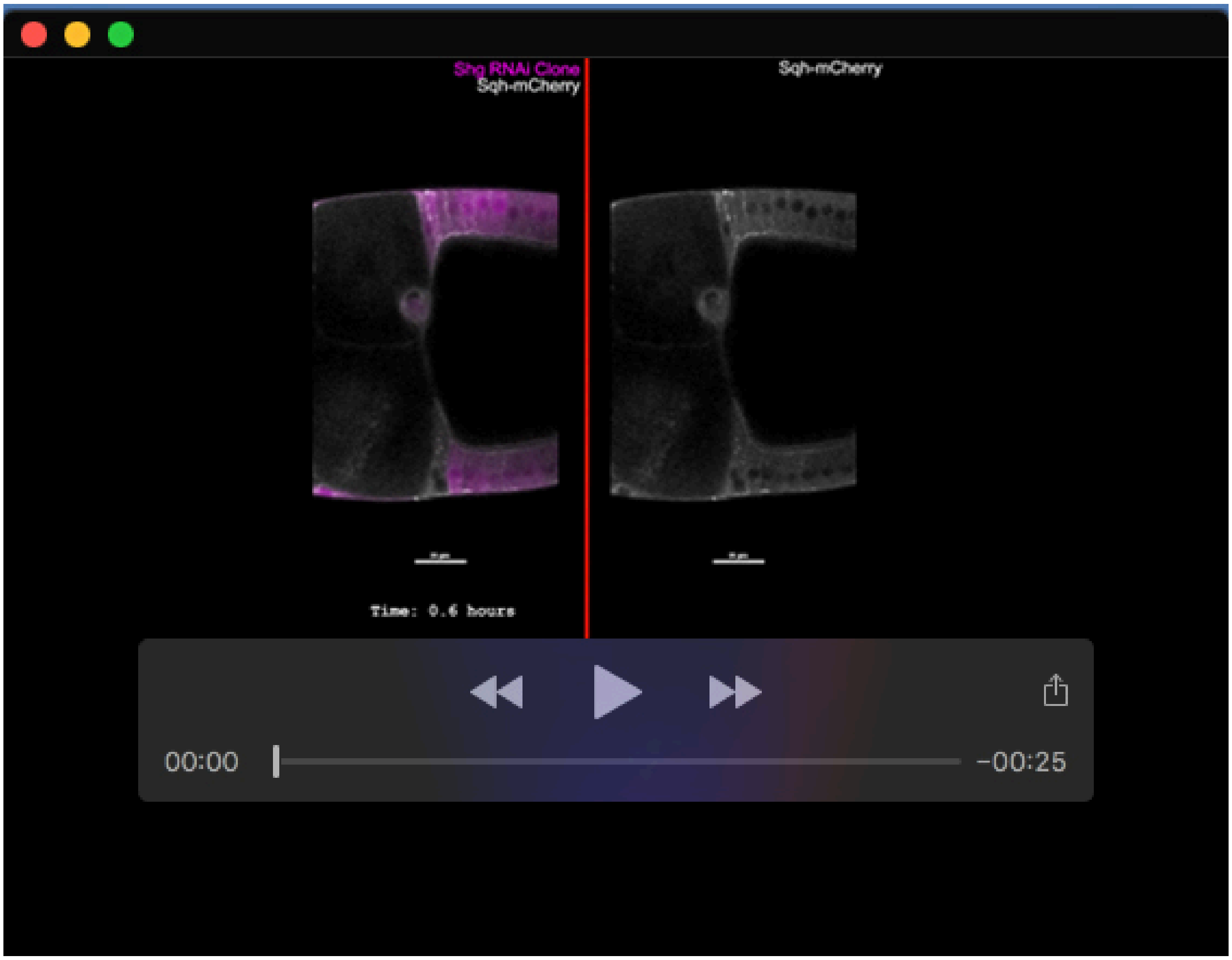

Movie 3, related to Figure 5: Knockdown of *shotgun* in follicle cells resulted in abnormal centripetal migration. A series of six time-lapse Movies consisting of the data from Fig. 5A-F showing phenotypes resulting from clonal knockdown of *shg* (parts 1 through 4) versus *luciferase* controls (part 5 and 6). Cross-sectional views are shown, with anterior oriented to the left and dorsal oriented up, except for part 6. Clones are created with the Flipout-Gal4 system and co-express UAS-regulated RNAi transgenes and GFP (magenta). Yellow arrows denote centripetal FCs of interest as indicated in Fig. 5. Phenotypes observed are as described in Fig. 5. Full genotypes for this Movie are available in Supplemental Table 2. Scale bars 25μm.

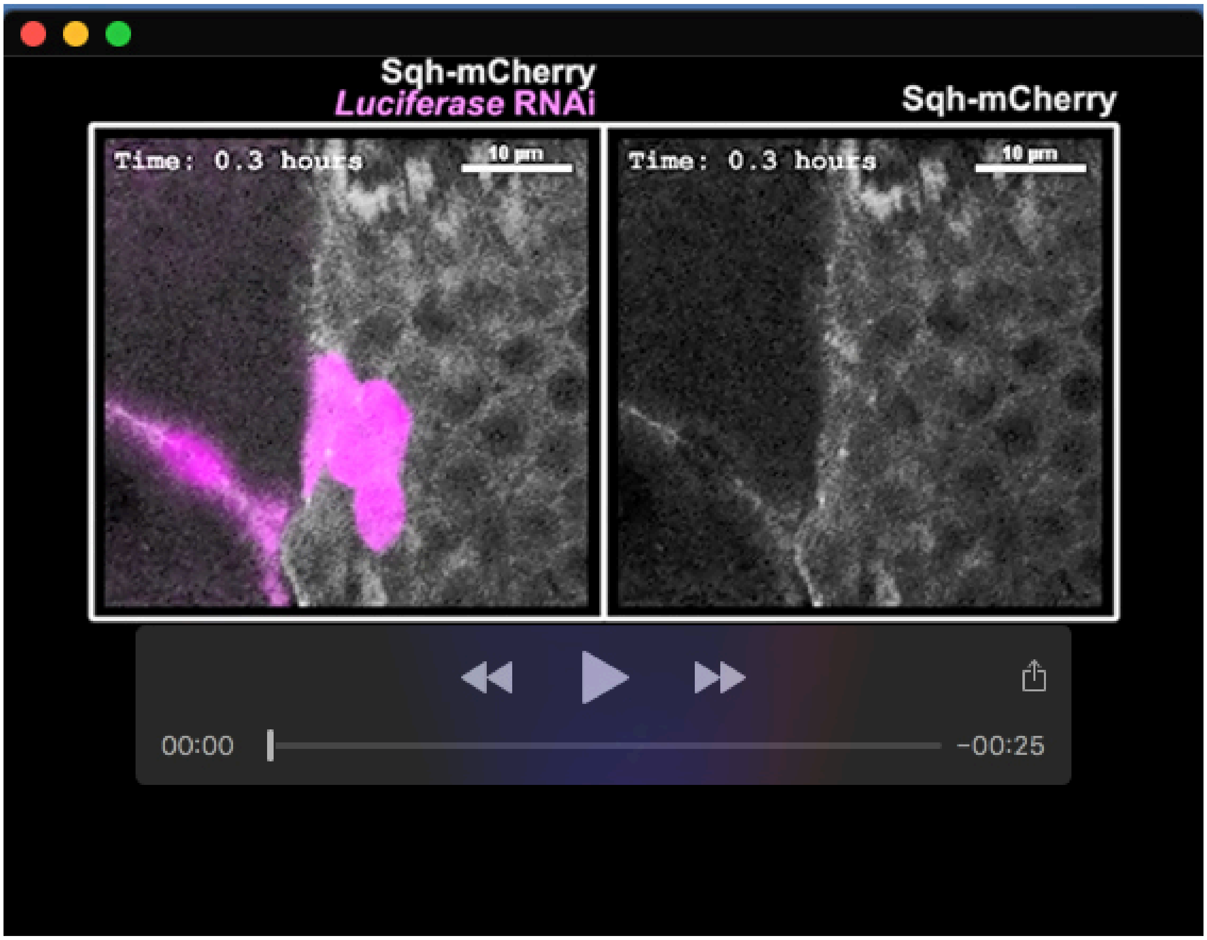

Movie 4, related to Figure 7, S8: Reduction of basal surface area is visible at the surface of stage 10B egg chambers undergoing centripetal migration. A series of two time-lapse Movies consisting of the data from Fig. S7A-F of stage 10B egg chambers undergoing centripetal migration as viewed from the surface. Egg chambers are labeled with Sqh-mCherry (white) and clonally express *luciferase* (part 1) or *shg* UAS-regulated RNAi transgenes (part 2) via the Flipout-Gal4 system (magenta). In video #1, the anterior-most centripetal FCs expressing *luciferase* UAS-regulated RNAi transgenes reduced their basal surface area at the surface of the egg chamber at a rate comparable to neighboring cells, whereas in video #2 columnar FCs expressing *shg* UAS-regulated RNAi transgenes appeared to be delayed in this process. Full genotypes for this Movie are available in Table S2. Scale bars 10µm.

Development • Supplementary information

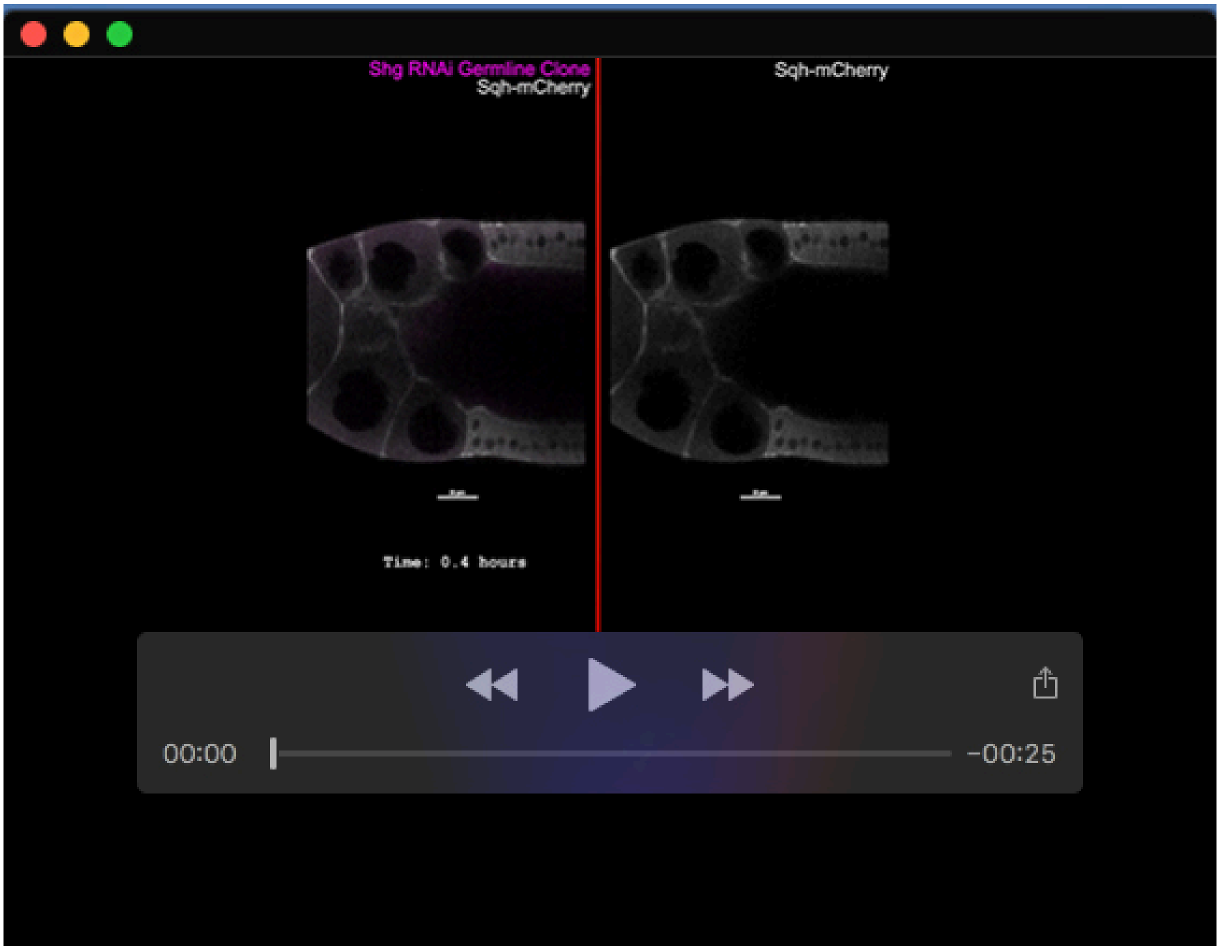

Movie 5, related to Figure S9: Knockdown of *shotgun* in germ cells resulted in abnormal but robust centripetal migration. A series of four time-lapse Movies consisting of the data from Fig. 5 S6A-D showing phenotypes associated with clonal germ cell knockdown of *shg* (parts 1 through 3) versus *luciferase* controls (part 4). Cross-sectional views are shown. Anterior is oriented to the left, and dorsal is up. Clones are created with the Flipout-Gal4 system and co-express UAS-regulated RNAi transgenes and GFP (magenta, yolk autofluorescence within the oocyte was also occasionally visible with this detection wavelength). Yellow arrows indicate centripetally migrating follicle cells. Part 3 shows an egg chamber with an oocyte that was mispositioned, resulting in an egg chamber with two anterior halves and duplicated centripetal migration events. Phenotypes observed are described in Fig. S9. Full genotypes for this Movie are available in Supplemental Table 2. Scale bars 25μm.

Development • Supplementary information

Supplemental Material References:

Godt, D. and Tepass, U. (1998). Drosophila oocyte localization is mediated by differential cadherin-based adhesion. *Nature 395*, 387-391.

González-Reyes, A. and St Johnston, D. (1998). The Drosophila AP axis is polarised by the cadherin-mediated positioning of the oocyte. *Development (Cambridge, England) 125*, 3635-3644.

Niewiadomska, P., Godt, D. and Tepass, U. (1999). DE-Cadherin is required for intercellular motility during Drosophila oogenesis. *The Journal of cell biology 144*, 533–547.

Oda, H., Uemura, T. and Takeichi, M. (1997). Phenotypic analysis of null mutants for DE-cadherin and Armadillo in Drosophila ovaries reveals distinct aspects of their functions in cell adhesion and cytoskeletal organization. *Genes to Cells* 2, 29–40.

Pfeiffer, B. D., Ngo, T. T., Hibbard, K. L., Murphy, C., Jenett, A., Truman, J. W. and Rubin, G. M. (2010). Refinement of tools for targeted gene expression in Drosophila. *Genetics 186*, 735-755.

Development • Supplementary information
