# Supplementary material for: Two phases for centripetal migration of Drosophila melanogaster follicle cells: initial ingression followed by epithelial migration
Source: Development. 2023 Mar 30;150(6):dev200492. doi: 10.1242/dev.200492 (PMC10112906; doi:10.1242/dev.200492)
Supplement: Supplementary file 1 [file develop-150-200492_review_history.pdf]

Development

Two phases for centripetal migration of *Drosophila melanogaster* follicle cells: initial ingression followed by epithelial migration

Travis T. Parsons, Sheila Mosallaei and Laurel A. Raftery
DOI: 10.1242/dev.200492

Editor: Thomas Lecuit

Review timeline

Original submission:	17 January 2022
Editorial decision:	7 March 2022
First revision received:	6 September 2022
Editorial decision:	10 October 2022
Second revision received:	11 January 2023
Accepted:	9 February 2023

Original submission

First decision letter

MS ID#: DEVELOP/2021/200492

MS TITLE: Two speeds for centripetal migration of Drosophila melanogaster follicle cells: Slow ingression followed by fast epithelial migration

AUTHORS: Travis T Parsons, Sheila Mosallaei, and Laurel A. Raftery

I have now received all the referees' reports on the above manuscript, and have reached a decision. The referees' comments are appended below, or you can access them online: please go to BenchPress and click on the 'Manuscripts with Decisions' queue in the Author Area.

As you will see, the referees express considerable interest in your work, but have some criticisms and recommend a revision of your manuscript before we can consider publication. The consensus view is that the results require proper quantification and statistical analysis to substantiate the claims. This is the main issue that must be addressed before considering the manuscript further. Obtaining higher resolution images may be difficult so I would let this to your appreciation whether or not this is possible in a reasonable time frame. Altogether the three reviewers make very useful suggestion to improve the manuscript. If you are able to revise the manuscript along the lines suggested, which may involve further experiments, I will be happy receive a revised version of the manuscript. Your revised paper will be re-reviewed by one or more of the original referees, and acceptance of your manuscript will depend on your addressing satisfactorily the reviewers' major concerns. Please also note that Development will normally permit only one round of major revision.

We are aware that you may be experiencing disruption to the normal running of your lab that make experimental revisions challenging. If it would be helpful, we encourage you to contact us to discuss your revision in greater detail. Please send us a point-by-point response indicating where you are able to address concerns raised (either experimentally or by changes to the text) and where you will not be able to do so within the normal timeframe of a revision. We will then provide further guidance. Please also note that we are happy to extend revision timeframes as necessary.

Please attend to all of the reviewers' comments and ensure that you clearly highlight all changes made in the revised manuscript. Please avoid using 'Tracked changes' in Word files as these are lost

© 2023. Published by The Company of Biologists under the terms of the Creative Commons Attribution License (https://creativecommons.org/licenses/by/4.0/).

in PDF conversion. I should be grateful if you would also provide a point-by-point response detailing how you have dealt with the points raised by the reviewers in the 'Response to Reviewers' box. If you do not agree with any of their criticisms or suggestions please explain clearly why this is so.

Reviewer 1

Advance summary and potential significance to field

In this paper, Parsons et al., present a study of centripetal follicle cell migration in the Drosophila ovary, First the authors define a timeline for wild type centripetal cell migration. In the second part, they generated shg mutant and RNAi clones to study the function of E-cadherin during centripetal cell migration. Overall, the data support the conclusions in this paper, but adding high magnification images and quantitative analysis would help readers better understand this interesting process.

Comments for the author

Major comments:
1. In general, a limitation of the manuscript in its current form is that high magnification images and quantitative analyses are lacking. For example,
A) Line 147-149, “Adjacent to the basement membrane, their basal regions progressively thinned to undetectable”, it would be helpful if the authors can label the cells in Fig 1D and 1E and add high mag images and also quantify the width of basement membrane at different time points (alternatively, providing the ratio of width at later time points compared to time 0 if the actual length varies a lot among samples).
B) Quantitative analysis should be added to Fig 3A-C. In Fig 3F, without high mag images, it is hard to discern the cell shapes.
C) High mag images should be added to Fig 5 and Fig 6.

2. The text and figures present the data in a different order. For example,
A) Line 269, the authors mentioned Supplemental Fig 7 before talking about Supplemental Fig 6.
B) Line 271, the authors mentioned Fig 5C before talking about Supplemental Fig 5B (line 279).
C) The same for Fig 6C (line 293) and Fig 6B (line 295).
D) The organization of Fig 5 should be changed to control (A, C, E) and shg-RNAi (B, D, F).

Minor comments:
1. Line 287-288, “Egg chambers…were selected for time-lapse imaging”. But Fig 6 is showing fixed images.
2. Line 298-300, “However, when only posterior following FCs were mutant, their migration appeared normal, unlike those with reduced E-Cadherin from RNAi”, what does it mean by “their migration”? Does it mean the “leading FCs”?

Reviewer 2

Advance summary and potential significance to field

This manuscript by Parsons et al makes headway into characterizing the interesting movement of centripetal cells in the Drosophila egg chamber. This movement has distinct features from those of better-characterized migration systems, which may reveal new genetic mechanisms that regulate collective cell migrations. The authors used high resolution, detailed live imaging to identify the stereotyped cell shape changes that occur and then classify them in detailed descriptions. As the title suggests, one interesting finding is that there are both slow and fast movements that must occur for the process to be completed. The authors also examine a role for neighboring stretch follicle cells in centripetal cell movement. Finally, they test the requirement for E-cadherin in both centripetal cells and germline cells and find that this gene (shg) is needed in both cell types, and it has autonomous and non-autonomous effects on movement when it is disrupted. Interestingly, there was still centripetal cell migration even when shg was reduced in nurse cells sufficiently to block border cell migration; this highlights the different molecular regulation at play in these two systems. The authors provide careful, detailed characterization of the process and define different milestones clearly, which significantly improves our understanding of this process. However, there

© 2023. Published by The Company of Biologists under the terms of the Creative Commons Attribution License (https://creativecommons.org/licenses/by/4.0/).

are still some aspects of the work that are confusing as it is currently presented; in particular, it was not always clear how the authors defined terms or made their conclusions, so additional analysis or more consistency in the descriptions would strengthen the manuscript.

Comments for the author

Major suggestions:

In the first part of the paper, the authors characterize the shape changes of leading migrating centripetal cells, but then they switch topics to describe a potential role for stretch follicle cells in the ingression. This second section is a bit confusing and seems out of place. The authors use a variety of Gal4 drivers to mark the stretch cells, which they describe in detail, but the labeling differs in terms of numbers of cells included. Thus, it wasn't entirely clear how to interpret the outcomes if there is mosaicism or variability in poorly-labeled cells. That is, if no stretch cell is detected, is it just not labeled? Most (61%) of the egg chambers had at least one visible stretch cell extended, but since one or two wouldn't touch the majority of the migrating centripetal cells the authors concluded that stretch cell ingression was not critical for centripetal cell ingression and that "centripetal migration sporadically includes extension of stretch FCs". Since the presence of an ingressed stretch cell could change the behavior of a neighboring leading centripetal cell, it is a bit hard to understand why this would occur randomly. The authors could more definitively determine if stretch cell involvement is critical by using RNAi with these Gal4 lines to try to block the movement in (and maybe there is some data for this in Figure 5), although this could be technically challenging if they end up not able to stretch at all. For presenting the work more clearly, I would recommend they completely describe the centripetal cell movements and all the milestones and move the stretch cell characterization after that.

The general description of centripetal cell migration (and the title) indicates that there are several slow ingression steps, then a fast movement of the leader cells and fast movements of follower cells. To substantiate the slow and fast descriptions, more quantification of cell speeds are needed. There is some description of this for the leader cell in milestone 5, shifting from 5 microns/2 hours to 10 microns/1.4 hours, but this is only for one example. The follower cells are described as "sliding" in milestone 6, but further characterization would help clarify the type of migration here (does the sliding mean there is no layering and no neighbor-position switching?), and quantification of speed per follower cell will reveal how this compares to the slow and fast leader cell movements.

The authors describe several outcomes due to loss E-cad (shg) in the follicle cells, which differ by location, and are much more severe with null mutant clones.
Large RNAi clones including the leader cells delayed migration, and loss in leader cells delayed basal thinning compared to normal cells on the opposite side; these phenotypes are consistent with the idea that the higher E-cad expression levels in these cells indicates a higher need for protein function. However, the authors claim that trailing mutant cells can non-autonomously affect leader cell basal thinning, but this is hard to see in Figure 5D and corresponding movie (compared to opposite side) and also in Figure 6B; since this step (milestone 3-4) has the greatest time variability (eg, Figure 4B), a bit more quantification on the length of the delays would help support this idea, as would as a model - it is not entirely clear to me why there would be a non-autonomous effect like this a few cells away. Non-autonomous effects are clearer due to germline loss of E-cad although disruption in nurse cells makes abnormal structures that the centripetal cells may not be able to move past.

Generally, it would be helpful to describe the mutant phenotypes in terms of milestones for clarity and to make the manuscript more cohesive.

Minor suggestions:
The abstract states that cells "slid", which could be more clearly described.

Abstract line 41 refers to "both populations", which seems to mean germline and somatic cells, but this is not explicitly stated.

© 2023. Published by The Company of Biologists under the terms of the Creative Commons Attribution License (https://creativecommons.org/licenses/by/4.0/).

I found the descriptions of the leading centripetal cell a bit confusing since in the first section they seem to describe one cell that elongates, but later the authors talk about 2-3 leader cells, and in the discussion they mention 2-3 rings of leader cells. Approximately how many are there? Also, in Figure 3E, the "leader" cell seems to stall and other cells move in front of it, so it wasn't clear if that still counted as a leader. An exact definition of how the authors know what is a leader cell and what is a follower cell would be helpful.

Figure 1 is very helpful, and the diagrams in B/C could be expanded to later times, perhaps in a summary figure to make the milestone results more clear. It seems like in the movies there is a larger proportion of centripetally migrating cells than the 4 green shown here.

In figure 3E and the corresponding movie, one of the leader cells (blue arrow) seems to come out of the epithelial layer and sit between moving centripetal cells and the nurse cells. is it common to have this multilayering situation? It seems relevant in describing the system because it may explain how the fast underlying cells could differ from those touching the nurse cells. The "follower" cells are described as rectangular (732), but they seem to be curved with narrow tops and bottoms and they look different from the columnar epithelial cells, so this could be explained more precisely.

At a late point in the paper the authors start referring to CMFCs (line 429), if they want to use it, they should define the term when they first describe centripetally migrating follicle cells in the introduction (page 4, line 112) or only use it in the legends.

Page 6, Line 193 in the phrase "a vertical line contiguous with the oocyte…" the word "contiguous" is confusing because it seems continuous earlier- maybe just "vertical" or "perpendicular to the outer edge of the egg chamber" would be clearer.

Reviewer 3

Advance summary and potential significance to field

This manuscript argues that centripetal migration of follicle cells occurs in two phases – 1) the cells near the nurse cell-oocyte boundary migrate slowly inward, and 2) the remaining cells slide rapidly over the anterior end of the oocyte. As part of their argument, the authors define a series of milestones for this process based on the morphology and follicle cells at different times. The first phase is shown to be independent of inward migration of the posterior-most squamous stretch cells, which only sporadically ingress. The authors went on to show that both phases require E-cadherin (shotgun in Drosophila). For the leading centripetal cells, E-cadherin is required autonomously for apical extension and non-autonomously for basal thinning, by which cells separate from the basement membrane. E-cadherin is also required in the germline to maintain its normal organization (Godt and Tepass, 1998); and disorganized germ cells affect the speed and shape of ingressing centripetal follicle cells.

Comments for the author

Overall, I thought the live imaging captured the process of centripetal cell migration with high temporal and spatial resolution. To my knowledge, this has not been done to this extent, before, and will be of high interest to the field. However, I think the authors conclusions would be greatly strengthened if they coupled their qualitative data with quantitation to support their key findings.

Main
1) Title and lines 392-394 - the authors make the conclusion that there are two different speeds/phases of migration: To make this statement, the authors should measure cell speed and determine if there is clear separation of phases. I believe the authors have the data to do this already, it is just a matter of making measurements to make the result rigorous.

2) Fig. 3, milestones: It would greatly strengthen the distinction between the different milestones if the authors quantified cell shape or speed and statistically compared sequential steps to determine that the differences are reproducible across egg chambers.

© 2023. Published by The Company of Biologists under the terms of the Creative Commons Attribution License (https://creativecommons.org/licenses/by/4.0/).

3) Fig. 5 – 7: When comparing control and RNAi/mutant embryos, would be better to have quantification and to show the spread of the data to be able to assess the significance of the effect and its reproducibility. I know they provide N's in the text, but would be much stronger to have a quantitative read out of observed differences.

4) Fig. 5-7: E-cadherin's effect could be via regulating cell-cell adhesion, but could also be through the ability of cadherin contacts to inhibit cell protrusion/locomotion (Bahm . . . Mayor, 2017). Did the authors look at how these treatments affect actin-based protrusion or could they comment more on how they interpret how these defects arise?

Minor
1. Text in figures is sometimes small and difficult to read (e.g. table contents in Fig. 2E, all scale bars)
2. It's mentioned in Line 223, but it might be useful to provide the approximate duration of centripetal migration in the introduction, or at least before the ΔT markings in Fig. 3 are introduced. Otherwise, the way in which times line up is somewhat confusing until Fig. 4 appears 3. Please clarify what the sample size is (cells or egg chambers) when sizes are provided (e.g. lines 148 153, etc.)
4. Lines 172-3 and Fig. 2: This Figure could use more explanation in the text for the reader to understand.
5. Paragraph starting in line 176: do the 36 half-egg chambers discussed include only those with >= 1 interior stretch cell? If so, should the range of "zero to >= 5" have 1 as the lower bound instead? From Fig. 2F the upper bound also looks to be 4 instead of 5.
6. Lines 226-7: Please elaborate on which results are referred to and explain more clearly how they suggest centripetal migration and nurse cell dumping are coordinated, this was unclear.
7. Lines 298-300: Does this mean the RNAi phenotype and shg-/- phenotype differ for the case in which there are WT leading cells and E-cadherin depleted following cells? If so, what could explain the difference?
-Adam Martin

First revision

Author response to reviewers' comments

We thank all the reviewers for their enthusiasm, careful reading of our manuscript, and insightful comments, and questions.

A. We have rewritten the manuscript tighten the focus, and clarify areas raised by the reviewers. In particular, we focused the analysis of E-Cadherin knockdown and mutant FCs, to just the leading and following centripetal FCs.

B. As we revised the manuscript, we realized that numerous errors had crept into the original submitted manuscript, from rushed revisions just prior to submission.
We apologize, particularly for confusing and incorrect methods descriptions for the analysis of *shg* mitotic clones in the original submission (lines 344 – 368, 406 -436 of original manuscript).

We have corrected and clarified these sections in revised submission (lines 319 – 351, 381 – 399).

C. All reviewers requested more quantification of the Milestones' defining features, and particularly for the speeds of migration during the two phases.

Response is enumerated below:

C1. Specific morphological features for each Milestone were selected for quantification:
Methods described in Supplementary Methods pages 4-6.
Dot plots of data with descriptive statistics for dataset are in Figure S1
Where relevant, statistical tests for significant differences were performed, as described in

© 2023. Published by The Company of Biologists under the terms of the Creative Commons Attribution License (https://creativecommons.org/licenses/by/4.0/).

Supplementary Methods, Fig. S1 legend, and the related results text.

C2. Quantification of speeds for each phase of the migration:

C2a. Measurement of forward progress for the leading edges of leading FCs could be tracked over about 1 hour, but was quite variable. We chose not to report these data, due to space constraints.

C2b. Measurement of forward progress for the leading edges of following FCs was more problemative. These FCs migrate anteriorly within the outer epithelium until they reach the oocyte corner. Each one moves around the corner during the span of 2 – 3 timepoints. It then rejoins the anterior epithelium to migrate inward. This movement encompassed few timepoints, which required use of a distinct NIS Elements function to determine "instantaneous speed."

C2c. We decided to focus instead on the shapes:

C2c1. The first leading FC elongates with thinning of the basal tip during Milestones 1 –III

C2c2. Following FCs temporarily shift away from a roughly quadrilateral shape as they move around the "corner" of the oocyte, and then resume a roughly quadrilateral shape as they rejoin the epithelium and move inward.

C2c3. These shapes changes are described in lines 210 – 205 of the revision.

C2c4. To emphasize the minimal changes between outside epithelium shape and shape as they move inward in the anterior epithelium, we determined the aspect ratios of multiple cells, first in the outside epithelium (before rounding the corner), and then in the inside, anterior epithelium (after rounding the corner). There is no significant difference between these aspect ratios.

C2c5. Our current imaging protocol lacks the temporal resolution to quantify the cell's shape or speed while it is "on" the corner (see Fig. 2D).

C2c6. We give more detail in the description of following FCs' movement as they go around the junction of the outer epithelium with the anterior epithelium (formed by ingressing leader FCs). We lacked temporal resolution to finely describe this movement.

2d. Movement of the 2nd and 3rd leading FCs during the Milestone III – V interval is mentioned during discussion of shg RNAi results.

Individual reviewers' comments:

-Reviewer 1:

- 1A—point 1 in Reference to prior Fig 1 and Fig 3, now Fig 1 and F2, respectively: Request for higher magnification images:

  Response:
  1. We have provided enlarged images derived from original time-lapse images, below the original panels.
  2. Technical limitations prevent capture of true higher magnification images for these, from our microscope objectives collect too little light at the tissue depth of the midline optical sections imaged for time-lapse sequences. As the panels in Figure 2 indicate, the midline is approximately 75um deep in the z-axis at the beginning of centripetal migration, and approximately 250um deep the in z-axis at the onset of nurse cell dumping (milestone 8). Most of these images were collected from live egg chambers using a water immersion objective NA 1.0.

- 1A—point 2: <u>Label the cells in Fig 1D & 1E, and quantify the decreased width of the basal region over time:</u>

  Response:
  For Fig 1 D,E, we outline the relevant cells. Quantitation of decrease width is provided in Fig. S1.

- 1B—point 1: <u>Quantitative analysis should be added to Figure 3A-C (now Fig. 2A-C):</u>
  Response:

© 2023. Published by The Company of Biologists under the terms of the Creative Commons Attribution License (https://creativecommons.org/licenses/by/4.0/).

Quantitative data are presented in Fig. S1.
• Note that we quantified basal width at Milestone II. For Milestone III, basal width is undetectable, so we listed the limits of detection for our objectives in the Supplementary Methods.
• Note that the Median width at Milestone II was 3.7 um, while the avg. limit of detection for Milestone III was 0.3 um.
• To avoid potential problems with using the limits of detection as Milestone III widths, we took a different approach to assess whether Milestones II and III were significantly different. We used the Wilcoxon test to compare Milestone II widths to a theoretic median of 0.3. Two-tailed P = <0.0001, indicating a significant difference.

- 1B—point 2: In Figure 3F, without high mag images, it is hard to discern the cell shapes:
  Response:
  The reviewer has raised a valid concern about detection of FC lateral side length.
  • Because of the weak signal this late during time-lapse imaging, we can see the width of the anterior epithelium, but few FC-FC interfaces can be distinguished.
  • We quantified the perceived width of the anterior epithelium, indicated by the dotted vertical lines in in Fig. 2E. Quantification is in Fig. S1E, showing a significant thickening.

- 1C—Higher mag images should be added to Fig 5 and Fig 6.

  Response:
  For Fig. 5, enlarged images of elongated shg-depleted FCs with opposite wildtype FCs were unwieldy. Sqh-mCherry was useful for this experiment, but lacked the fine detail provided in the wild type experiment. See lines 127 – 137 for a discussion of the limitations of our time-lapse imaging method.
  For Fig. 6, the faint F-Actin staining in follicle cells was just as difficult to see in enlarged images.

- 2—The text and figures present the data in a different order.

  Response: We have corrected this in the revised version.

Reviewer 1 Minor comments:

1. "Line 287-288, "Egg chambers…were selected for time-lapse imaging". But Fig 6 is showing fixed images."

Response:
Thank you for catching this.
This is now corrected, as part of the bigger correction presentation of the mitotic clone data.

2. "Line 298-300, "However, when only posterior following FCs were mutant, their migration appeared normal, unlike those with reduced E-Cadherin from RNAi", what does it mean by "their migration"? Does it mean the "leading FCs"?

Response:
This phrase was vague, and referred to phenotypes of those shg-/- FCs among the most-posterior following FCs. and/or more posterior mainbody FCs.
We have removed descriptions of phenotypes associated with decreased E-Cadherin in FCs more distant from the leading FCs, to clarify and focus o phenotypes associated with decreased E-Cadherin in leading FCs and/or neighboring following FCs.

-Reviewer 2:

- Reviewer 2 raised a number of questions about interpreting the outcomes with the stretch cell analysis. This reviewer suggests an RNAi experiment to block stretch cell extension using the same Gal4 drivers, and then acknowledges that the results of such an experiment might also be ambiguous.

© 2023. Published by The Company of Biologists under the terms of the Creative Commons Attribution License (https://creativecommons.org/licenses/by/4.0/).

Response:
We acknowledge Reviewer 2's concerns, and have decided to de-emphasize these results
In line 262, we simply state that "Cell-to-cell variability precluded analysis of stretch FCs' behavior."

- "For presenting the work more clearly, I would recommend they completely describe the centripetal cell movements and all the milestones, and move the stretch cell characterization after that."

  Response:
  This is an excellent suggestion.
  The stretch cell section now follows the Milestones section, starting on line 243.

- "The general description of centripetal migration ... indicates that there are several slow ingression steps, then a fast movement of the leader cells and fast movement of follower cells. ... more quantification of cell speeds are needed."

  Response:
  All reviewers requested quantification of speeds.
  We discuss our difficulties in evaluating Phase 2 speed of individual follower cells in section C of the general responses above.

  For the slow ingression steps:
  We measured leading edge speed for inward movement in the 60min period used to measure elongation at Milestone I which was captured in different samples than those that exhibited continued elongation during the original Milestone V of the submitted manuscript. The 42 samples for the original Milestone V varied between about 0.1 um/min. to about 0.6 um/min, with a similar range for the 7 samples from Milestone I.

  We decided to eliminate the original Milestone V (faster ingression speed), and chose not to report these data.
  The resubmitted manuscript now lists the shift to Phase 2 migration as Milestone V.

- "The follower cells are described as 'sliding' in milestone 6, but further characterization would help clarify the type of migration here (does the sliding [in Milestone 6] mean that there is no layering and no neighbor-position switching?), and quantification of speed per follower cell will reveal how this compares to the slow and fast leader cell movements."

  Response:
  Reviewer 2 raises an important point about our use of "sliding" to decribe follower FC movement.
  In the revised manuscript, we provide a more detailed description of the following cell morphologies as they "round the corner" to move inward in lines 204 – 211. Follower cell shapes contrast with the elongated shape of Leader FCs during Milestones I — III, including the III —IV interval – described in lines 177 – 190, with an example in Fig. S7.

  Because the follower FCs move from the outer epithelium to the inner, anterior epithelium over a span of about 3-4 images (20 -30 min or so), we focused on the morphology of the follower cells just before they round the corner, and shortly after they round the corner,.
  These roughly quadrilateral shapes are quantified as Height:Width aspect ratio, and are not significantly different (Fig. S1F).

- "...does the sliding [in Milestone 6] mean that there is no layering and no neighbor-position switching?"
  Neighbor switching and layering start to clearly appear during Milestone V, below the location of the follower "rounding the corner (see Fig. 2E). More cell rearrangements likely occur during Milestones VI and VII, when they are difficult to follow. Other

© 2023. Published by The Company of Biologists under the terms of the Creative Commons Attribution License (https://creativecommons.org/licenses/by/4.0/).

variables, such as the dorsal-ventral orientation of the egg chamber, may influence these later rearrangements.

- Reviewer 2 raises a number of concerns about our reports of non-autonomous effects on leader cell migration from loss or knock-down of E-cadherin in more posterior "trailing" cells.

Response:
We chose to remove any discussion of more distant posterior E-Cadherin depleted FCs, which gave us space to provide better descriptions of phenotypes in leader FCs and associated with follower FCs.

Reviewer 2 Minor comments:

1. The abstract states that cells "slid", which could be more clearly described.

Response:
The abstract word limit constrains our description of follower FCs' movement around the corner between the outside epithelium and the inner, anterior epithelium.
We hope the minimal description in lines 43-44 is less objectionable.

2. Abstract line 41 refers to "both populations", which seems to mean germline and somatic cells, but this is not explicitly stated.

Response:
We changes this to "both groups of centripetal FCs " in line 45 of the abstract.
We chose emphasize spatial distinctions in FC E-cadherin requirements, because the differences in germ cell E-cadherin phenotypes were difficult to encapsulate.

3. I found the descriptions of the leading centripetal cell a bit confusing since in the first section they seem to describe one cell that elongates, but later the authors talk about 2-3 leader cells, and in the discussion they mention 2-3 rings of leader cells. Approximately how many are there?

Response:
In the first section (Fig. 1D,E), we only focus on the first leading FC.
We removed any reference to "2-3 leading FCs", and only refer to 3. We now believe there are 3 rings of leading centripetal FCs, but sometimes only two are apparent in an x-y midline section, when the FC columnar epithelium is tightly packed against the stretch cells, during stage 10 to early stage 10B.
The columnar FCs are hexagonally packed, so different x-y optical sections can look slightly different.

In the x-y midlines cross sections, we generally see 3 leading FCs in an x-y A-P column of the FC epithelium.
However, the leading centripetal FCs are present around the circumference of the egg chamber, so that there is a "ring" of FCs, about 3 cells wide. A portion of this ring is visible in surface views of the epithelium, such as diagrammed in Fig. 1A. We cite several reviews that describe this organization.

4. Also, in Figure 3E, the "leader" cell seems to stall and other cells move in front of it, so it wasn't clear if that still counted as a leader. An exact definition of how the authors know what is a leader cell and what is a follower cell would be helpful.

Response:
We provide more detailed descriptions of leading and following cells in the Milestones section, lines 177-192 for leading FCs, and lines 204-211 for following FCs.

The possible presence of a stretch FC extension along the surface of a nurse cell, complicates the interpretation of a thin, extended FC in this position (now seen in Fig. 2D). In some

© 2023. Published by The Company of Biologists under the terms of the Creative Commons Attribution License (https://creativecommons.org/licenses/by/4.0/).

videos of control GFP+ RNAi cells, we see a long, thin GFP+ cell extending along a nurse cell (with portions in and out of view in different z-sections or timepoints), in which the GFP+ extending cell may be the extension of a contiguous GFP+ surface stretch cell. As discussed in the section on stretch FC extensions, now lines 243- 262,visualization of stretch cells was inadequate to resolve this issue in our experiments.

5. Figure 1 is very helpful, and the diagrams in B/C could be expanded to later times, perhaps in a summary figure to make the milestone results more clear. It seems like in the movies there is a larger proportion of centripetally migrating cells than the 4 green shown here.

Response:
This would be a great illustration, but we focused mostly on data analysis for the revision. We changed Fig. 1A-C to include green leading FCs and turquoise following FCs, but did not add diagrams of later timepoints.

6. In figure 3E and the corresponding movie, one of the leader cells (blue arrow) seems to come out of the epithelial layer and sit between moving centripetal cells and the nurse cells. is it common to have this multilayering situation? It seems relevant in describing the system because it may explain how the fast underlying cells could differ from those touching the nurse cells.

Response:
Please see our response to minor comment 4 above.
We agree that the FC extending along a nurse cell could have an advantage, especially if that cell is a stretch FC. Unpublished work by other labs studying stretch FCs may illuminate this.
However, a small number of samples that exhibit a nurse cell-adjacent extending cell, which might or might not be a stretch cell, are not sufficient to address this speculations. We hope our live-imaging protocol will be useful to those who have stretch FC tools sufficient to resolve this question.

7. The "follower" cells are described as rectangular (732), but they seem to be curved with narrow tops and bottoms and they look different from the columnar epithelial cells, so this could be explained more precisely.

Response:
We expanded the follower cell description to include a better description of their shape, and how it changes for most follower cells as they round the corner (lines 204-210). In particular, we describe them as "roughly quadrilateral", to distinguish from the "rounded" phenotypes from E-cadherin reduction in leading FCs.

We agree that occasional follower cells, in single timepoints of some samples, appear curved with narrow tops and bottoms. We interpret these as possible transitional FCs between the leading FCs and the following FCs. Their rare presence suggests that greater temporal and spatial resolution are need to interpret such cells.

8. At a late point in the paper the authors start referring to CMFCs (line 429), if they want to use it, they should define the term when they first describe centripetally migrating follicle cells in the introduction (page 4, line 112) or only use it in the legends.

Response:
Thank you for pointing this out.
We replaced this abbreviation with leading FCs where appropriate, or centripetal FCs where we refer to all centripetally migrating FCs.

9. Page 6, Line 193 in the phrase "a vertical line contiguous with the oocyte…" the word "contiguous" is confusing because it seems continuous earlier- maybe just "vertical" or "perpendicular to the outer edge of the egg chamber" would be clearer.

© 2023. Published by The Company of Biologists under the terms of the Creative Commons Attribution License (https://creativecommons.org/licenses/by/4.0/).

Response:
We revised the wording in the description of Milestone IV, lines 193-199.
To directly illustrate the changing angle, we added yellow lines in Fig. 2C and Movie 2.

Reviewer 3:

- 1—"The authors make the point that there are two phases/speeds of migration: to make this statement, the authors should measure cell speed and determine if there is a clear separation of phases."

  All reviewers had a similar request. Our response is in C2 of the general responses at the beginning.

- 2—"It will greatly strengthen the distinction between the different milestones if the authors quantified cell shape or speed and statistically compared sequential steps to determine if differences are reproducible across egg chambers."

  All reviewers had a similar request. Our response is in C1 of the general responses at the beginning.

- 3—"Fig 5 – 7: When comparing control and RNAi/mutant embryos, would be better to have quantification and show the spread of data..."

  For shg RNAi and mutant FCs, we provide more detail, separating the phenotypic descriptions by the type and numbers of FCs with altered phenotypes.
  Phenotypes are quantified temporally by the Milestones affected, and for live-imaging with RNAi, whether delays or stalled migration occur in every case, or whether individual FCs are variably affect (in the case of leading FCs).

  For those phenotypes in wild type FCs adjacent to mis-positioned nurse cells, the specific nurse cell and its distance displaced vary between each egg chambers.
  We now quantify three general knockdown nurse cell phenotypic classes:

  A) Egg chambers with a rounded knockdown germ cell, where wild type centripetal FCs migrate inward with normal timing, but either early elongated FCs, or late anterior epithelial FCs, appeared to conform to the shape of a rounded nurse cell (lines 368 – 372).

  B) Egg chambers with disorganize knockdown nurse cells, in which the oocyte bulged into the nurse cell-region of the egg chamber: we quantify the number of egg chambers with this phenotype out of the total with disorganized knockdown nurse cells, and the number of the latter, where wild-type centripetal FCs extended to abut the oocyte. This last phenotype occurs in only 7 of 18 with disorganized knockdown germ cells, but was none-the-less striking.

- 4—"Did the authors look at how these treatments affect actin-based protrusion or could they comment on how they interpret how these defects arise?"

  Response:
  Experiments to examine actin cytoskeletal remodeling and actin-based projections were not appropriate for the spatial resolution of our method. These interesting and important questions about expected protrusive activity at the leading edge of leading FCs are "next steps" for future studies that build upon the foundation established in this work.
  Intriguing Sqh-mCherry accumulation in one example of control leading FC ingression (Fig. S7 of revised manuscript), suggests that myosin activity at the basal edge prior to attachment would be fruitful to explore. Such an investigation was beyond the scope of the work reported in this manuscript. I

© 2023. Published by The Company of Biologists under the terms of the Creative Commons Attribution License (https://creativecommons.org/licenses/by/4.0/).

Reviewer 3 Minor comments:

1. Text in figures is sometimes small and difficult to read (e.g. table contents in Fig. 2E, all scale bars)

Response:
The scale bar text is repeated in each figure legend. The figure contains the scale bar output with the image from NIS Elements.
We apologize for small text in the table, now in Fig. 4. Two out of three authors routinely view figures of papers by enlarging on-screen, leading to low priority for this item during revision.

Graphics in Fig 4 will be fixed and replace, if the article is accepted.

2. It's mentioned in Line 223, but it might be useful to provide the approximate duration of centripetal migration in the introduction, or at least before the ΔT markings in Fig. 3 are introduced. Otherwise, the way in which times line up is somewhat confusing until Fig. 4 appears

Response:
We added the time frame to line 127, at the beginning of the results section.

3. Pease clarify what the sample size is (cells or egg chambers) when sizes are provided (e.g. lines 148, 153, etc.)

Response:
We have clarified this in the revised manuscript, lines 164, 261, 375, 378,
Also, sample size for quantification is shown in Figure Legends, and Supplemental Methods section for methodology for Fig. S1.

4. Lines 172-3 and Fig. 2: This Figure could use more explanation in the text for the reader to understand.

Response:
The limitations of confocal imaging preclude obtaining high quality z-axis optical sections across the diameter of a late stage 10B egg chamber, which are necessary for volume projections to view stretch FC extensions. We could not squeeze an explanation into the text in this area, or to the legend of Fig. 2.
Instead, we added a statement about this to lines 235-237 of the legend to Fig. S4 in the revised supplementary materials. Averaged images from fast resonance scanning with a high NA objective were used to generate volume projections for Figs. 4, S3, S4 of the revised manuscript, optical sections were obtained through the half egg chamber closest to the objective. The method is described in lines 611-619.

In contrast, low resolution resonance scanning optical sections (lines 220-221 in Fig. S3 legend, supplemental material(were used to only project a fuzzy full-diameter volume of a fixed egg chamber at late stage 10B for Fig. S3.

5. Paragraph starting in line 176: do the 36 half-egg chambers discussed include only those with>= 1 interior stretch cell? If so, should the range of "zero to >= 5" have 1 as the lower bound instead? From Fig. 2F,the upper bound also looks to be 4 instead of 5.

Response:
The legend for Fig 4E states these numbers correctly in lines 766 -772.
Lines 260 – 263 now clarify that 36 out of 59 half-egg chambers had one or more detectable stretch FC extensions, which means that 23 half egg chambers had zero.
Fig. 4E identifies Class 4 egg chambers as those with 5+ non-labelled exterior stretch FCs.
We apologize for missing this deceptively "minor" comment about the inconsistent dot plots in this figure, until it was too late to prepare a revised figure.

© 2023. Published by The Company of Biologists under the terms of the Creative Commons Attribution License (https://creativecommons.org/licenses/by/4.0/).

6. Lines 226-7: Please elaborate on which results are referred to and explain more clearly how they suggest centripetal migration and nurse cell dumping are coordinated, this was unclear.

Response:
We removed this statement. It was an interpretation of the average separation of leading edges in lines 228-230, just before nurse cell dumping. “Tight coordination of centripetal migration with nurse cell dumping” has been suggested by research papers and reviews on *Drosophila* oogenesis, going back to 1970, or before.

7. Lines 298-300: Does this mean the RNAi phenotype and shg-/- phenotype differ for the case in which there are WT leading cells and E-cadherin depleted following cells? If so, what could explain the difference?

Response:
Perhaps not—
Wildtype leading FCs next to knockdown following FCs, showed elongation with variably delayed basal constriction starting at the time for Milestone III, lines 306-312 of the revised manuscript.
Wild type leading FCs next to shg-/shg-following FCS were examined in fixed egg chambers, which showed a snapshot of aberrantly rounded leading FCs at an indeterminate Milestone, described in lines 335-337.
Because fixation captures a moment in the process of leading FC ingression, the fixed shg-/- samples may be a frozen image of the variably delayed basal constriction of knockdown leading FCs, so that they appear to be rounded and clustered, with a delayed leading FC caught at the basement membrane above a successfully detached mutant FC located away from the basement membrane.
This possibility, and potential alternative explanations for the shg-/- following FC phenotype are listed in lines 343-348.

Second decision letter

MS ID#: DEVELOP/2021/200492

MS TITLE: Two phases for centripetal migration of Drosophila melanogaster follicle cells: initial ingression followed by epithelial migration

AUTHORS: Travis T Parsons, Sheila Mosallaei, and Laurel A. Raftery

I have now received all the referees reports on the above manuscript, and have reached a decision. The referees' comments are appended below, or you can access them online: please go to BenchPress and click on the 'Manuscripts with Decisions' queue in the Author Area.

The overall evaluation is positive and we would like to publish a revised manuscript in Development, provided that the concerns of 2 of the referees can be satisfactorily addressed. In particular Referee 3, who likes the manuscript, requests a better assessment of variability of the phenotypes reported in Figure 5. Among the possible measurements: 1) leading centripetal cell apical extension length, 2) basal width of leading cell (Milestone II transition), and 3) nuclear position/movement of following centripetal follicle cells (i.e. measuring transition to Milestone V) would seem most appropriate.

Please attend to all of the reviewers' comments in your revised manuscript and detail them in your point-by-point response. Please send us a point-by-point response indicating your plans for addressing the referee’s comments, and we will look over this and provide further guidance.

© 2023. Published by The Company of Biologists under the terms of the Creative Commons Attribution License (https://creativecommons.org/licenses/by/4.0/).

Reviewer 1

Advance summary and potential significance to field

I appreciate the authors' effort to provide quantitative data in the revised manuscript. I think this paper should be published. But as they discussed in the paper and the response, due to the technical limitations, they simply cannot capture higher magnification of images during CFC migration. They do show enlarge images and quantified data.

Comments for the author

I think overall their observations are credible, and agree that CFC migration is worthy of further study.

Reviewer 2

Advance summary and potential significance to field

Parsons et al provide a very detailed characterization of the movement of centripetal cells in the Drosophila egg chamber. These cells may be of particular interest because they are plastic and can become secretory during their movements and they are highly amenable as a model system for further discoveries about this process through genetics and imaging. The authors provide striking high-resolution live and fixed images and detailed, organized descriptions to create a framework to divide centripetal cell collective migration into key milestones.
They go on to test the roles for E-cadherin in this process, identifying different cell types and steps in which it is required.

Comments for the author

The revised version of the manuscript is significantly improved; it is easier to follow now, and the reviewers have satisfied my concerns. I have no major comments.

A few minor suggestions:
The summary statement says the collective migration of follicle cells is needed to "complete" the epithelium, but it isn't obvious what it is meant by this -another word may be better here.

In the introduction (line 118-9), the authors state "E-Cadherin appeared dispensable in following FCs, as long as leading FCs migrated normally" which is true but seems a bit misleading since it seems to be non-autonmously required in the following FCs - the authors may want to reword this.

Reviewer 3

Advance summary and potential significance to field

Overall, I thought the live imaging captured the process of centripetal cell migration with high temporal and spatial resolution. To my knowledge, this has not been done to this extent, before, and will be of high interest to the field. However, I have serious issues with the lack of rigor/statistics in key experiments that support the authors' findings.

Comments for the author

The authors have not satisfactorily addressed the reviewer comments regarding quantification. One of the major comments by all reviewers was the lack of quantification. While I appreciate their improving the quantitative description of the different phases of migration in wild type, there is still a lack of quantitative rigor in supporting the main experiment in the manuscript - i.e. the experiment testing E-Cadherin function.
There is no objective quantification and, thus, no statistical analysis comparing mutant and wild-type. The authors report the number of egg chambers imaged/analyzed, which is not quantitative analysis, nor is that a rigorous way to compare control and experimental samples. I can't support

© 2023. Published by The Company of Biologists under the terms of the Creative Commons Attribution License (https://creativecommons.org/licenses/by/4.0/).

publication of a manuscript where there is such an omission, regarding a pivotal part of the conclusions.

Second revision

Author response to reviewers' comments

We thank the reviewers and the editor for their comments and suggestions, which have greatly improved the manuscript.

We performed additional data analyses on the *shg*-depleted FCs compared to the controls, as follows:

1. Time interval between Milestone II and Milestone III was significantly longer for the *shg*-depleted leader FCs 1 and 2, **new Fig. S8**.
2. GFP+ Leader FCs 1 and 2 showed a significantly smaller decrease in basal width, measured either from Milestone II to Milestone III, or from Milestone II to the end of the time-lapse sequence, when all such samples were pooled, **new Fig. S9**, and in some classes of samples, when these were compared separately, **new Fig. S12**.
3. GFP+ leader FC 1 show a significantly longer apical length increase, measured either from Milestone II to Milestone III, or from Milestone II to the end of the time-lapse sequence, when all such samples were pooled, **new Fig. S11**, and in some classes of samples, when these were compared separately, in the **new Fig. S13**.
4. Four instances of Milestone V were observed in the control samples, and one in a *shg*-depleted GFP+ sample. We measured the times for each transition from Milestone III to Milestone V, reported in a dot plot in the **new S14**.
5. I measured the anterior displacement of nuclei representing following FCs 4-6 and mainbody FC 7, from Milestone III to Milestone V, or from Milestone III to the end of the time-lapse sequence, for FCs in each of our three classes of clones, data presented in the **new Fig S15**. This analysis was subject to variables outside of the FCs, as discussed in an expanded section on image analysis in the Materials and Methods.
6. I consulted with a physicist who designs optics and analyses performance of optical instruments, for suggestions of alternative methods that might be less subject to extrinsic variation, but the approaches suggested had variation from the different external variable tradeoffs. A higher-powered analysis of the requirement for E-Cadherin in Phase 2 migration of following cells will require a different method of clone induction/marking, and a much larger sample size.

Data analyses 2, 3 and 5 are the metrics suggested in the decision letter of 10/10/2022.

Overall, our data strongly support autonomous requirements for E-Cadherin in the leading FCs that result in delayed basal thinning, accompanied by a significantly longer interval between Milestones II and III. Apical extension continues over this prolonged transition, giving significantly longer FCs 1 and 2 at Milestone III or the end of the time-lapse sequence. This suggests that basal thinning is more sensitive to reductions in E-Cadherin levels that apical lengthening. Non-autonomous effects were seen in the *shg* mutant analysis with mitotic clones, but sample sizes were too small to tease these out in the RNAi analysis.

Our deep dive into the Milestone III to Milestone V transition uncovered the rare occurrence of Milestone V in the control samples. The sample size was smaller than that used for the initial Milestone analysis, but there may be other effects from the Sqh::MCherry, the heat-shock induced expression of dsRNA in clones of FCs, or other factors in the genetic background for these experiments.

Altogether, we have added discussion of new metrics from items 1-5, and removed most interpretation of changes in the Milestone V transition and following FCs movements, from the main text. Major changes affection the results section:

© 2023. Published by The Company of Biologists under the terms of the Creative Commons Attribution License (https://creativecommons.org/licenses/by/4.0/).

1. lines 266-295: per the change requested after our 1/8 submission, we moved the section “Extrinsic factors influence milestones” to the main text, and revised to meet the new word limit
2. lines 320-330: Added an explanation of the classes of samples based on GFP+ leaders and/or followers
3. lines 334-357: results of analyses for Milestone II – Milestone III metrics
4. lines 358-365: results of analyses for Milestone V and transition to Milestone V
5. lines 388-390: mention the observation of 3 examples of rounded cells in RNAi samples
6. lines 394-398 and 492-498: focused conclusions on strongest data
7. lines 706-767: new methods section on selection of RNAi clones and image analysis
8. lines 768-782: new methods section on statistics

Minor changes:

9. lines 28-31: Per Reviewer 2’s request, we have lengthened the Summary Statement to better reflect the nuances of anterior FC epithelial morphogenesis and function.
10. lines 117-118: Reviewer 2 thought this statement was misleading, based on observations of non-autonomous effects of *shg* mutant or E-cadherin-depleted follower FCs on ingression of GFP- leader FCs. We made this change to reflect the *shg* mutant follower FC effect. The statistical analysis for the GFP+ follower FC only class in Figs. S12, S13 showed no significant difference between the *shg*-depleted leader FCs, and the control leader FCs. Larger sample sizes will be needed to assess whether a significant non-autonomous effect is seen for *shg*-depleted follower FCs, in spite of the greater variance in RNAi datasets .

Altogether, these analyses support the main conclusions of our work, as stated in the title, abstract, and discussion. While we previously over-interpreted our data to suggest *shg* requirements in the transition to Milestone V, this was a minor aspect of the total work.

Third decision letter

MS ID#: DEVELOP/2021/200492

MS TITLE: Two phases for centripetal migration of Drosophila melanogaster follicle cells: initial ingression followed by epithelial migration

AUTHORS: Travis T Parsons, Sheila Mosallaei, and Laurel A. Raftery
ARTICLE TYPE: Research Article

I am happy to tell you that your manuscript has been accepted for publication in Development, pending our standard ethics checks.

Reviewer 3

Advance summary and potential significance to field

This manuscript argues that centripetal migration of follicle cells occurs in two phases – 1) the cells near the nurse cell-oocyte boundary migrate slowly inward and 2) the remaining cells slide rapidly over the anterior end of the oocyte. As part of their argument, the authors define a series of milestones for this process based on the morphology and follicle cells at different times. The first phase is shown to be independent of inward migration of the posterior-most squamous stretch cells, which only sporadically ingress. The authors went on to show that both phases require E-cadherin (shotgun in Drosophila). For the leading centripetal cells, E-cadherin is required autonomously for apical extension and non-autonomously for basal thinning, by which cells separate from the basement membrane. E-cadherin is also required in the germline to maintain its normal organization (Godt and Tepass, 1998); and disorganized germ cells affect the speed and shape of ingressing centripetal follicle cells. The authors have now provided quantitative measurements for many of the steps and to compare wild-type and knock-down embryos.

© 2023. Published by The Company of Biologists under the terms of the Creative Commons Attribution License (https://creativecommons.org/licenses/by/4.0/).

Comments for the author

Minor comment:
Line 320: 'IWe' should be corrected.

© 2023. Published by The Company of Biologists under the terms of the Creative Commons Attribution License (https://creativecommons.org/licenses/by/4.0/).
